# Pathogenic mechanisms of cardiovascular damage in COVID-19

**DOI:** 10.1186/s10020-024-00855-2

**Published:** 2024-06-19

**Authors:** Hong-Hua Shao, Rui-Xing Yin

**Affiliations:** 1 Department of Infectious Diseases, HIV/AIDS Clinical Treatment Center of Guangxi (Nanning), The Fourth People’s Hospital of Nanning, No. 1 Erli, Changgang Road, Nanning, Guangxi, 530023 People’s Republic of China; 2 Department of Cardiology, Institute of Cardiovascular Diseases, The First Affiliated Hospital, Guangxi Medical University, 6 Shuangyong Road, Nanning, Guangxi, 530021 People’s Republic of China

**Keywords:** COVID-19, SARS-CoV-2, Cardiovascular damage, Myocardial injury, Myocarditis, Hypertension, Arrhythmia

## Abstract

**Background:**

COVID-19 is a new infectious disease caused by the severe acute respiratory syndrome coronavirus 2 (SARS CoV-2). Since the outbreak in December 2019, it has caused an unprecedented world pandemic, leading to a global human health crisis. Although SARS CoV-2 mainly affects the lungs, causing interstitial pneumonia and severe acute respiratory distress syndrome, a number of patients often have extensive clinical manifestations, such as gastrointestinal symptoms, cardiovascular damage and renal dysfunction.

**Purpose:**

This review article discusses the pathogenic mechanisms of cardiovascular damage in COVID-19 patients and provides some useful suggestions for future clinical diagnosis, treatment and prevention.

**Methods:**

An English-language literature search was conducted in PubMed and Web of Science databases up to 12th April, 2024 for the terms “COVID-19”, “SARS CoV-2”, “cardiovascular damage”, “myocardial injury”, “myocarditis”, “hypertension”, “arrhythmia”, “heart failure” and “coronary heart disease”, especially update articles in 2023 and 2024. Salient medical literatures regarding the cardiovascular damage of COVID-19 were selected, extracted and synthesized.

**Results:**

The most common cardiovascular damage was myocarditis and pericarditis, hypertension, arrhythmia, myocardial injury and heart failure, coronary heart disease, stress cardiomyopathy, ischemic stroke, blood coagulation abnormalities, and dyslipidemia. Two important pathogenic mechanisms of the cardiovascular damage may be direct viral cytotoxicity as well as indirect hyperimmune responses of the body to SARS CoV-2 infection.

**Conclusions:**

Cardiovascular damage in COVID-19 patients is common and portends a worse prognosis. Although the underlying pathophysiological mechanisms of cardiovascular damage related to COVID-19 are not completely clear, two important pathogenic mechanisms of cardiovascular damage may be the direct damage of the SARSCoV-2 infection and the indirect hyperimmune responses.

**Supplementary Information:**

The online version contains supplementary material available at 10.1186/s10020-024-00855-2.

## Introduction

COVID-19 is a novel identified acute infectious disease caused by severe acute respiratory syndrome coronavirus 2 (SARS-CoV-2). Since the outbreak in December 2019, it has caused an unprecedented world pandemic, leading to a global human health crisis (Li et al. 2020; Wang et al. 2020a). As of December 24, 2023, a total of 773,119,173 persons have been infected with COVID-19 worldwide. Of them, 6,990,067 people died, with an overall mortality rate of 1% (Choi et al. 2024; Hatch et al. 2024) to 4% (Shu et al. 2023). At present, the prevalence of COVID-19 has slightly improved with viral mutation and population vaccination, but the number of infections and deaths is still rising. Although COVID-19 has been initially associated with respiratory system, it has become rapidly clear that it may affect multiple important organs including the heart (Tomasoni et al. 2020; Del Vecchio et al. 2024). COVID-19 may directly exacerbate pre-existing heart disease and frequently induce new cardiovascular complications (Burger et al. 2021). There is a two-way relationship between COVID-19 and cardiovascular diseases. Pre-existing cardiovascular risk factors, such as hypertension, diabetes, and chronic cardiovascular diseases, are easy to cause serious COVID-19. On the contrary, COVID-19 can lead to cardiovascular complications (Ozcan et al. 2023). The mechanisms involving these cardiovascular complications of COVID-19 may include direct myocardial injury, systemic inflammation and cytokine storm, downregulation of angiotensin-converting enzyme 2 (ACE2, EC 3.4.17.23) receptors, mismatch of myocardial oxygen demand-supply, atherosclerotic plaque rupture and coronary thrombosis, electrolyte imbalances, diffused endothelial damage, coagulation abnormalities characterized by hypercoagulation and microthrombosis, and adverse effects of COVID-19 therapies (Parvu et al. 2022; Zhao et al. 2023; Ozcan et al. 2023; Tangos et al. 2024). According to previous reports, 19–28% of COVID-19 patients experience cardiac damage (Shi et al. 2020; Guo et al. 2020b; Bonow et al. 2020), 33% of COVID-19 deaths can also be attributed to heart disease (Zheng et al. 2020; Ruan et al. 2020). The occurrence of cardiovascular complications may also affect the severity and increase mortality of COVID-19 (Santoso et al. 2021; Tian et al. 2020). Although the clinical manifestations and laboratory test results in COVID-19 patients have been reported widely, the data regarding the pathogenetic mechanisms of cardiovascular damage in COVID-19 patients remain scant. Therefore, this review article addresses the cardiovascular damage caused by SARS-CoV-2 infection and its pathogenetic mechanisms. It may provide some valuable reference materials for clinicans to future diagnose, treat and prevent.

## Search methodology

A literature search was performed through systematical search for current finding mainly from PubMed and Web of Science databases from 12th December, 2019 to 12th April, 2024. The literature search was restricted to publications in English or translated into English. The search strategy of medical subject headings and keywords combined with entry terms was utilized to search all related literatures. The keywords were selected based on the previous publications, and all the terms used were based on the criterion that they were present in the titles, abstracts, and keywords of the articles. The following defined words were used as search strategy to obtain relevant information: “COVID-19”, “SARS CoV-2”, “cardiovascular damage”, “myocardial injury”, “myocarditis”, “hypertension”, “arrhythmia”, “heart failure” and “coronary heart disease”, especially update articles in 2023 and 2024. The inclusion of references was based on documents displayed information on cardiovascular damage and myocardial injury associated with COVID-19. Salient medical literatures regarding the cardiovascular damage of COVID-19 were selected, extracted and synthesized.

## Structure and biological properties of SARS-CoV-2

**Fig. 1:**
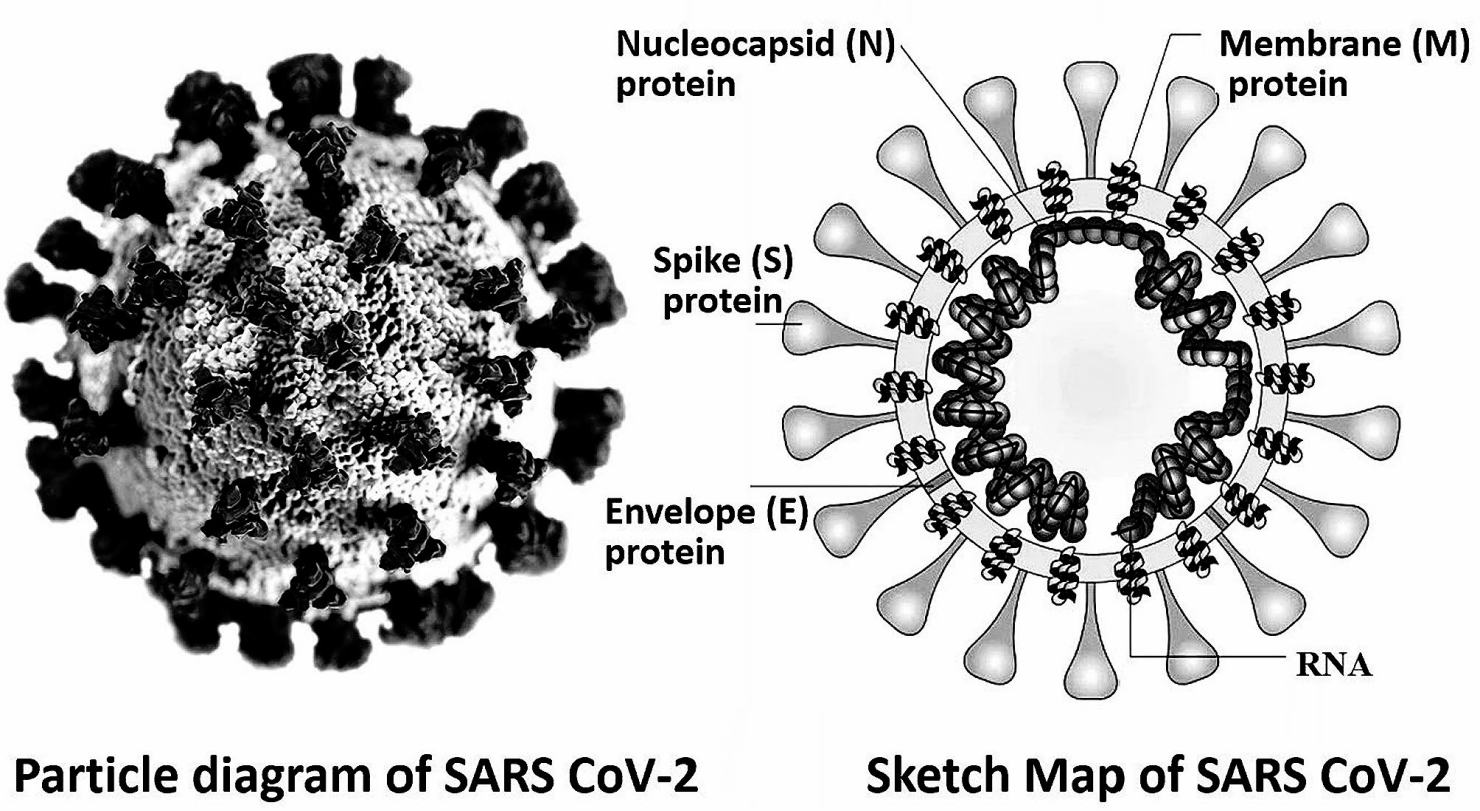
Structural properties of the SARS-CoV-2. Severe acute respiratory syndrome coronavirus 2 (SARS-CoV-2) consists of five components: an RNA gene chain and four proteins. The outermost layer is spike (S) protein, with a molecular weight of 180,000-200,000, which is composed of 1,200-1,500 amino acids and contains 21–35 N-glycosylation sites. Several S proteins form a special spiky corolla structure on the virus surface in the form of trimer (about 600 kDa). The main function of S protein is to bind to the ACE2 receptor on the surface of human cells. It is one of the largest class I fusion proteins known, which enables virus particles to fuse into cells for replication and produce more next-generation virus particles. The envelope (E) protein and membrane (M) glycoprotein below the spike are components of the envelope of virus particles, which protect the RNA gene chain inside the virus. M protein is also involved in the assembly and release of next generation virus particles, which plays an important role in the structural stability and functional expression of S, E and N proteins. Inside the virus is a spiral nucleoprotein core composed of RNA gene chain and nucleocapsid protein (N). N protein plays an important role in virus replication. RNA gene chain is an RNA chain compounded in N protein, which is composed of 29,891 nucleotides (about 30,000 nucleotides) in series. Nucleotide G and nucleotide C account for about 40%. The main function of RNA gene chain is to preserve the genetic code of the virus so that the next generation of virus particles can be replicated

**Fig. 2:**
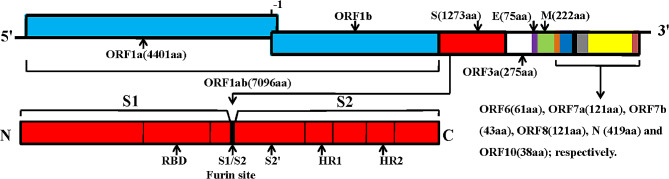
Names and distributions of 12 open reading frames from 5’ to 3’ ends of SARS CoV-2 gene and the number of translated amino acids. The number in brackets is the number of amino acids in translation. The S protein contains S1 and S2 subunits and a single transmembrane (TM) anchor. S protein binds to the angiotensin converting enzyme 2 (ACE2) receptor on the cell surface through the receptor binding domain (RBD), which is an essential step in membrane fusion. Activation of S requires Furin protease like protease to cleave S1/S2 and undergo conformational changes from pre fusion to post fusion. At present, several pre fusion conformations of S protein are known, among which three RBDs show different directions, namely “up” or “down”. The receptor binding sites are exposed only when RBD adopts the “up” conformation

The newly discovered SARS CoV-2 is the seventh human coronavirus. It belongs to β Genus Mesosak β Coronavirus subgenus (Guzik et al. 2020). The viral particle is often polymorphic, with a diameter of 50–200 nm, and it is a single-stranded positive-sense RNA virus with genomes ranging from 26.2 to 31.7 kb RNA. SARS CoV-2 has four major structural proteins: spike (S), nucleocapsid (N), membrane (M), and envelope (E) proteins, all required to produce the viral particle (Huang et al. 2020; Schoeman and Fielding 2019; Chan et al. 2020a; Fig. 1). SARS CoV-2 can bind to the surface-bound peptidase ACE2 or CD26 receptors leading to tissue infection and viral replication (Hoffmann et al. 2020; Lim et al. 2020; Chappell 2023). When SARS-CoV-2 binds to ACE2, internalization of the SARS-CoV-2-ACE2 complex reduced ACE2 activity, and subsequent activation of the rennin-angiotensin-aldosterone system [RAAS; higher angiotensin (Ang) II/Ang-(1–7) ratio] that may exacerbate the acute inflammatory events in COVID-19 patients and contribute to the effects of long COVID-19 (Chappell 2023). In addition, it can activate metalloproteinase 17 (ADAM17), which induces ACE2 membrane shedding, exacerbates the accumulation of Ang II, and diminishes the cardioprotective effects of ACE2 (Gheblawi et al. 2020; Zhao et al. 2023). Moreover, COVID-19 patients present with an array of autoantibodies to various components of the RAAS including the peptide Ang II, the enzyme ACE2, and the AT1, AT2 and Mas receptors (Chappell 2023). The density of ACE2 receptors is very high on cell surface of many tissues and organs including human type II alveolar epithelial cells, macrophages and other cell types, esophageal epithelial cells and layered epithelial cells, absorptive intestinal epithelial cells of ileum and colon. In addirion, ACE2 is involved in the regulation of several cardiovascular and immune pathways (Zheng et al. 2020; Hooper et al. 2020). Increased expression of plasma soluble ACE2 was found in patients with cardiovascular diseases such as myocardial infarction, atrial fibrillation, valvular disease, and heart failure, reflecting a higher basal ACE2 expression and increased susceptibility in these conditions (García-Escobar et al. 2022; Silva et al. 2022). Currently, gene expression studies showed that human ventricular myocardium contains all the requisite mediators of SARS-CoV-2 binding and entry. In the heart, ACE2 is expressed more in cardiomyocytes and pericytes than that in endothelial cells and fibroblasts (Chung et al. 2021; Shu et al. 2023). In addition, pericytes, which support the microvasculature throughout the myocardium, appear particularly susceptible with robust expression of ACE2 (Chung et al. 2021). Moreover, S protein has a strong binding affinity with ACE2 receptor, and its binding free energy is − 50.6 kcal/mol (Xu et al. 2020a). Gastrointestinal tract may also be a potential pathway of SARS CoV-2 infection (Zhang et al. 2020). S protein includes S1 and S2 subunits (Fig. 2). The S1 subunit has the N-terminal domain (S1-NTD) and C-terminal domain (S1-CTD). These regions are the sites binding to various cellular receptors, and the binding domains of these receptors contain carbohydrates or proteins. For example, the receptor binding domain (RBD) of S1 can bind to the ACE2 receptor on the cell surface. This process requires viral S protein activation through the transmembrane serine protease 2 (TMPRSS2). The S2 subunit of the trimer has two heptapeptide repeat (HR) sequences of HR1 and HR2, which mediate the fusion of the virus with the infected cell membrane. In addition, the protease Furin is also needed to promote entrance of the virus into the cell (Walls et al. 2020). There is also a conserved Furin recognition site on the S1/S2 cleavage site of SARS-CoV-2, which is directly related to the high pathogenicity of the virus. The second protease cleavage site called S2’ is located near the N-terminus of S2. The genome size of SARS-CoV-2 is 29,891 nucleotides, including the untranslated region (UTR) gene at both ends and a complete open reading frame (ORF) gene, which can encode 9,860 amino acids (Ren et al. 2020; Liu et al. 2020a). The genome of SARS CoV-2 has a cap structure at the 5’ end and a polyadenylate poly (A) tail at the 3’ end. From the 5’ end to the 3’ end, there are 12 ORFs respectively: ORF1a, ORF1b, S, ORF3a, E, M, ORF6, ORF7a, ORF7b, ORF8, N and ORF10 (Walls et al. 2020; Chan et al. 2020a). Among them, there are two large reading frames ORF1a and ORF1b in the first 2/3 of the genome, which can encode two replicase related multi protein precursors pp1a and pp1ab. SARS-CoV-2 needs to transpose a nucleotide (–1) from the end of ORF1a to the 5’ end to synthesize 1b through the mechanism of ribosome reading frame displacement, and finally translate into the complete multi protein precursor pp1ab. The genome of SARS CoV-2 is highly variable at two core locations (silent variation in ORF1ab gene and polymorphism in ORF8 amino acid). The mutation in ORF8 will lead to two variants (ORF8-L and ORF8-S), and induce structural abnormalities of the protein (Ceraolo and Giorgi 2020). For example, a single N501T mutation may significantly strengthen the binding affinity of SARS CoV-2 RBD and human ACE2. In addition, S and N proteins are also prone to mutation (Benvenuto et al. 2020).

## Epidemic characteristics of COVID-19

The transmission route of COVID-19 may be bat-human or bat-intermediate host-human. Pigs, ferrets, cats and primates may be their intermediate hosts (Wan et al. 2020; Lake 2020; Andersen et al. 2020; Lu et al. 2020b). After the cross species transmission of wild animals to humans, there is also the transmission between people. Therefore, infected patients and asymptomatic infected people are the main source of infection of COVID-19 (Andersen et al. 2020; Rothe et al. 2020). Cholesterol in the host cell plasma membrane plays an important role in the SARS-CoV-2 entry into cells (Chidambaram et al. 2022b). The transmission of SARS CoV-2 increases proportionally with rising levels of cholesterol in the cell membrane. This is due to the fact that cholesterol increases the number of viral entry spots and the concentration of ACE2 receptor, crucial for viral penetration (Kowalska et al. 2022). The infectiousness is very high within 5 days after onset of COVID-19. The important epidemic feature is aggregation, such as community or family aggregation transmission (Lian et al. 2020). The incubation period is generally 1 to 14 days, mostly 3 to 7 days, and very few cases can reach 24 days (Chan et al. 2020b; Chen et al. 2020c; Jin et al. 2020). The basic infection number (RO) value of SARS CoV-2 at the initial outbreak stage is greater than 1:2.2 to 2.9 (Li et al. 2020; Callaway et al. 2020; Zhao et al. 2020), indicating that it is highly infectious (Jiang et al. 2020). The viral load of infected persons was more than 1 billion RNA copies per ml of sputum. Severe COVID-19 patients often had higher viral load and longer viral transmission period than the mild patients. Respiratory droplets and close contact are two main routes of transmission. Contact with viral contaminated items such as saliva, nasal mucus, feces and urine can also cause infection (Holshue et al. 2020). It is unclear whether there is feces-mouth or contaminated food-mouth transmission. When exposed to high concentrations of aerosols for a long time in a relatively closed environment, it is possible to propagate through aerosols. Therefore, attention should be paid to contact transmission or aerosol transmission caused by environmental pollution. There are also reports of the possibility of mother to child vertical transmission. Contact transmission is caused by droplets containing SARS CoV-2 or other excreta of patients contaminating the surface of objects. The hands are usually the last link of infection after human contact. After hands are contaminated, they are infected by touching the face, nasal mucosa and oral mucosa. Aerosol transmission refers to small droplets floating in the air, which can be transmitted over a long distance through inhalation of respiratory tract infection. The symptoms of pediatric cases are relatively mild, and some children and newborns may experience atypical symptoms (Shen and Yang 2020; Vasichkina et al. 2023). The aged, people with chronic basic diseases (hypertension, diabetes, cardiovascular diseases, etc.), late pregnancy and perinatal women, and obese individuals are seriously ill after infection (Zheng et al. 2020; Chan et al. 2020b; Wu et al. 2020; Zhou et al. 2020a; Tang et al. 2020).

## Pathogenic mechanisms of cardiovascular damage in COVID-19

**Fig. 3:**
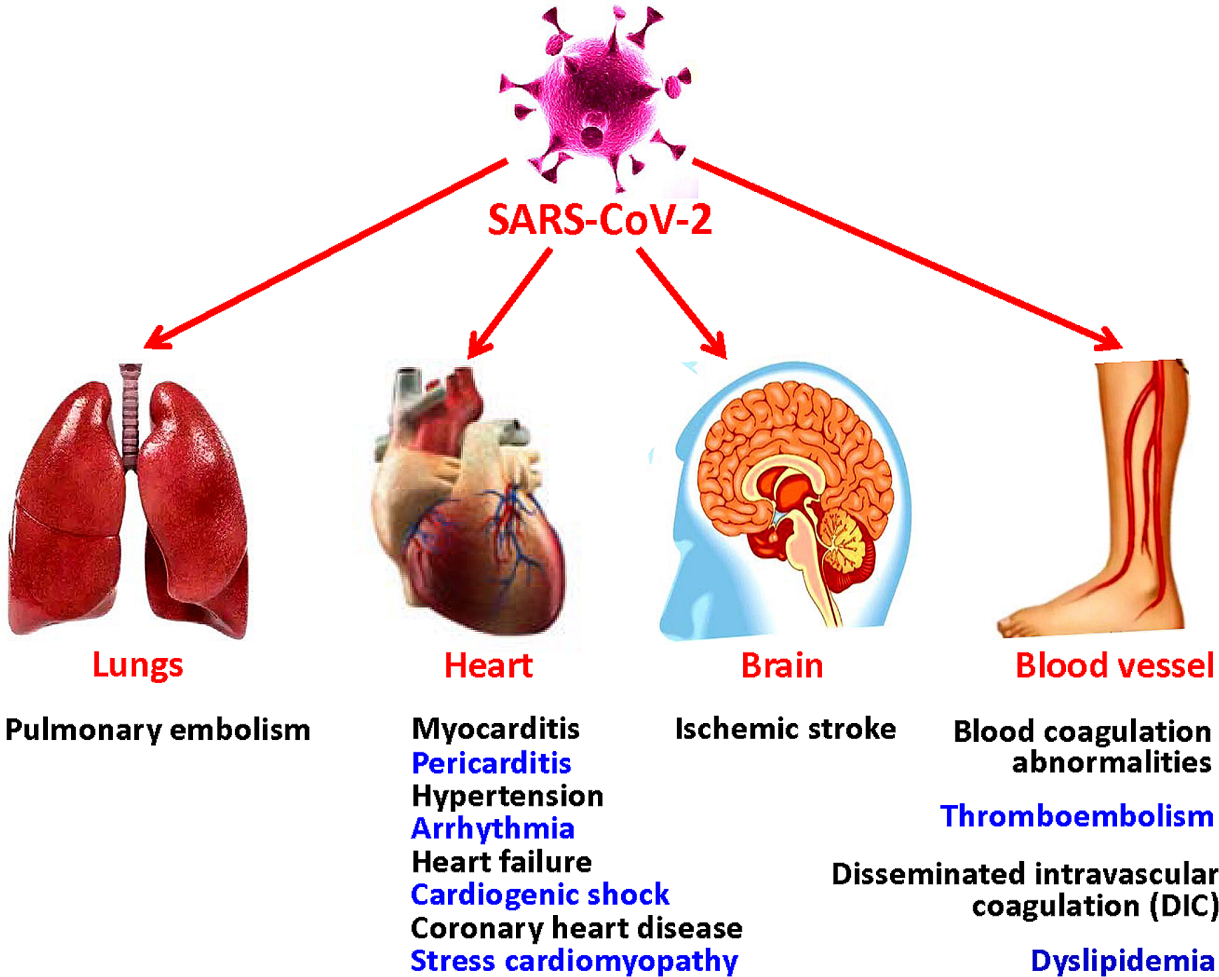
Cardiovascular damage of the SARS-CoV-2 infection

COVID-19 not only impacts the respiratory system but also exhibits extrapulmonary involvement, resulting in systemic disease (Ozcan et al. 2023). Therefore, the clinical presentations of COVID-19 are multifaceted and vary widely, mainly include the general features of respiratory infection and the special manifestations of extrapulmonary complications such as cardiovascular, cerebrovascular, gastrointestinal, musculoskeletal, endocrine, and renal systems. Many patients with COVID-19 can develop different types of cardiovascular complications during hospitalization (Pellicori et al. 2021), which may be a part of post-acute infection sequelae (Tsampasian et al. 2024). Approximately 62% of the COVID-19 hospitalized patients had acute myocardial injuries (Shu et al. 2023). Myocardial involvement may be a feature of long COVID syndrome from the early months of the COVID-19 pandemic (Tsampasian et al. 2024). About 45% of COVID-19 survivors experienced persistent symptoms at 4 months post the acute infection (O’Mahoney et al. 2022; Tsampasian et al. 2024; Adu-Amankwaah 2024). Systematic review confirms that chest pain, palpitations, dyspnoea, and syncope are the most commonly symptoms among patients with long COVID syndrome (Tsampasian et al. 2024). The patients with underlying cardiac disease or the presence of cardiovascular disease risk factors are more likely to experience serious outcomes (Tian et al. 2020). The clinical manifestations of cardiovascular damage in COVID-19 patients include myocarditis and pericarditis, hypertension, arrhythmia, myocardial injury and heart failure, coronary heart disease (CHD), stress cardiomyopathy, ischemic stroke, blood coagulation abnormalities, and dyslipidemia (Adu-Amankwaah et al. 2021; Zhao et al. 2023; Tsampasian et al. 2024; Fig. 3). Long COVID-19 can affect almost all organs of the body, and can lead to more than 200 different clinical manifestations (Gyöngyösi et al. 2023). There are strong evidences demonstrating that pre-existing obesity, heart failure, and ischemic heart disease are significant risk factors for the development of long COVID syndrome. However, there is conflicting data in literature about other cardiovascular diseases such as hypertension, cholesterol, atrial fibrillation, and diabetes mellitus (Tsampasian et al. 2024).

There may be racial and ethnic disparities in COVID-19 outcomes. Patients from ethnic minority groups are disproportionately affected by COVID-19 (Magesh et al. 2021). A previous meta-analysis showed that the risk of SARS CoV-2 infection was higher in the individuals of Black and Asian ethnicities compared to White individuals. Moreover, Asians may have higher risk of intensive therapy unit admission and death (Sze et al. 2020). Higher mortality rates of COVID-19 were also found in Black and Latinx populations (Gross et al. 2020; Yancy 2020), and in predominantly Black compared with White populated counties (Yancy 2020; Scannell et al. 2020). In an analysis of 7,868 hospitalized patients with COVID-19, Hispanics, non-Hispanic Black, Asian, and non-Hispanic White were 33%, 25.5%, 6.3%, and 35.2%; respectively. However, Asian patients had the highest cardiorespiratory severity score (Rodriguez et al. 2021). In the UK, COVID-19-related disease severity and mortality were higher in Black, Asian, mixed and other ethnic groups than in the White ethnic group majority (Siddiq et al. 2023). A meta-analysis comprising 4,318,929 patients from 68 studies also showed that White race, Hispanic/Latino, African American, multiracial and another race or ethnicity, Asian American, American Indian or Alaska Native, Pacific Islander account for 24.0%, 19.7%, 8.6%, 6.2%, 2.4%, 0.2%, and 0.2%; respectively. African American and Hispanic individuals were the most likely to test positive for COVID-19. Asian American individuals had the highest risk of intensive care unit (ICU) admission (Magesh et al. 2021). Compared with White majority populations, Irizar et al. found an increased risk of testing positive for infection for people from Black, South Asian, Mixed, and other ethnic groups. Black, Hispanic, and South Asian people were more likely to be seropositive. Among population-based studies, Black and Hispanic ethnic groups and Indigenous peoples had an increased risk of hospitalization. Black, Hispanic, South Asian, East Asian and Mixed ethnic groups and Indigenous peoples had an increased risk of ICU admission. Mortality risk was increased for Hispanic, Mixed, and Indigenous groups. Smaller differences were seen for prognosis following infection. Following hospitalisation, South Asian, East Asian, Black and Mixed ethnic groups had an increased risk of ICU admission, and mortality risk was greater in Mixed ethnic groups (Irizar et al. 2023).

The progression and outcome of COVID-19 may have sex differences (Widmann et al. 2024; Tangos et al. 2024). Several studies have revealed that the morbidity, severity, and mortality of COVID-19 were higher in men than in women (Chen et al. 2020d; Haitao et al. 2020; Alwani et al. 2021; Wehbe et al. 2021; Marik et al. 2021; Torres et al. 2023; Woodruff et al. 2023; Chappell 2023). The prevalence of COVID-19 in combination with cardiovascular disease was also higher in male than in female (Gebhard et al. 2020). The mortality in patients with acute myocardial infarction and COVID-19 (Yeo et al. 2023) or heart failure and COVID-19 (Isath et al. 2023) was higher in men than in women. In a systematic review and meta-analysis, there were also different in the sex, racial, and ethnic representation among COVID-19 prevention and treatment trials (Xiao et al. 2023). These findings suggest that men may have higher susceptibility to and more severe adverse clinical outcomes from SARS-CoV-2 infection than women. The reasons for sex differences in COVID-19 may be attributed to the difference in the expression levels of ACE2 and TMPRSS2, sex hormones, and immune and inflammatory responses (Viveiros et al. 2021; Thomas et al. 2021; Zhao et al. 2023). Plasma ACE2 concentrations and TMPRSS2 expression were higher in males than in females, TMPRSS2 is regulated by androgens (Okwan-Duodu et al. 2021), which may lead to an increased initial viral load (Viveiros et al. 2022). ACE2 and TMPRSS2 are key factors in promoting SARS-CoV-2 entry into cells (Hoffmann et al. 2020). Females can express higher amounts of toll-like receptor (TLR) 7, which recognizes single-stranded RNA and promotes interferon (IFN) production, playing an important role in the immune response to SARS-CoV-2 infection (Bienvenu et al. 2020; Wehbe et al. 2021; Zhao et al. 2023). Estradiol enhances the antiviral response by increasing the number of neutrophils and natural killer cells and decreasing pro-inflammatory cytokines, whereas androgens have immunosuppressive effects (Viveiros et al. 2021; Bechmann et al. 2022; Brandi 2022). Androgen receptors are transcription promoters for TMPRSS2 and can facilitate SARS-COV2 entry. Genetic variants in the androgen receptor were correlated with androgen sensitivity (Mohamed et al. 2021). However, Bugiardini et al. found that women in general wards were at increased risk of acute heart failure and in-hospital mortality for COVID-19 compared with men. For patients receiving ICU care, fatal complications including acute heart failure and mortality appeared to be independent of sex (Bugiardini et al. 2023).

Age is an uncontrollable risk factor for increased mortality in COVID-19 patients. The susceptibility is higher as well as the clinical outcomes is more severe in older than in younger people (Bonanad et al. 2020; Zhou et al. 2020a; Torres et al. 2023; Tangos et al. 2024). Several previous studies also demonstrated that the risk of cardiovascular complications was greatly increased in the elderly COVID-19 patients. The risk of cardiovascular disease in COVID-19 patients was increased with age (Pellicori et al. 2021). The expression of ACE2 in SARS-CoV-2 infected cells was reduced, especially in elderly people. Thus, elderly people with down-regulated ACE2 expression have more severe adverse outcomes when infected with SARS-CoV-2 (AlGhatrif et al. 2020). These findings suggest that different individuals and populations have different tolerances and reactions to the COVID-19 (Liu et al. 2021b; Tobler et al. 2022). However, Giugni et al. found that younger age was associated with cardiovascular pathological phenotype of severe COVID-19 at autopsy. They showed that younger age was associated with cardiovascular abnormalities such as acute heart ischemia, myocarditis and lung angiomatosis, whereas older age was associated with pulmonary findings including exudative diffuse alveolar damage, proliferative diffuse alveolar damage lung squamous metaplasia and lung viral atypia (Giugni et al. 2024).

### Myocarditis and pericarditis

Myocarditis and pericarditis are the most common in COVID-19 patients. They may also be potential post-acute cardiac sequelae of SARS-CoV-2 infection, arising from adaptive immune responses (Tuvali et al. 2022; Smer et al. 2023). SARS-CoV-2 infection may lead to acute viral myocarditis and pericarditis (Ho et al. 2020; Siripanthong et al. 2020; Patone et al. 2022; Castiello et al. 2022; Sawalha et al. 2021; Liu et al. 2021a; Daniels et al. 2021; Caforio et al. 2022; Gnecchi et al. 2020; Yokoo et al. 2020; Chiu et al. 2020; Warchoł et al. 2020; Fairweather et al. 2023), or even fulminant myocarditis (Chen et al. 2020a; Hu et al. 2021; Zeng et al. 2020; Kitsou et al. 2024). The incidence of myocarditis/pericarditis after COVID-19 was at least 15 times higher than that before COVID-19. The prevalence of myocarditis before COVID-19 was 1 to 10 cases/100,000 people, while that after COVID-19 was 150 to 4,000 cases/100,000 individuals (Fairweather et al. 2023). In a total of 176,137 hospitalizations with confirmed SARS-CoV-2 infection in Germany, 226 (0.01%) had myocarditis (incidence: 1.28 per 1000 hospitalization-cases). Independent risk factors for myocarditis in COVID-19 were age < 70 years, male, pneumonia, and multisystemic inflammatory responses to SARS-CoV-2 infection. Myocarditis was independently associated with increased case-fatality (Keller et al. 2023). COVID-19-related myocarditis may only manifest as palpitations or mild chest discomfort, which may not be differentiated with other causes of most patients. In the majority of cases, fever, shortness of breath, cough and chest pain were the most common presenting symptoms (Ho et al. 2020; Chimenti et al. 2022). In some patients, however, myocarditis could lead to fulminant disease. The cardiac and inflammatory biomarkers were elevated in most patients. Temporary ECG changes such as non-specific ST-segment and T-wave changes and ventricular tachycardia are common and may be helpful to determine the presence and severity of myocardial injury (Ho et al. 2020). Myocarditis may develop into conduction block, tachyarrhythmia and impaired left ventricular function. When myocardial injury is detected in the absence of an acute coronary syndrome, myocarditis should be highly suspected. Left ventricular dysfunction and hypokinesis were common. A diagnosis can usually make if cardiac magnetic resonance imaging (MRI) detects a typical signal of acute myocardial injury. When cardiac MRI is not feasible, cardiac computed tomographic angiography with delayed myocardial imaging may be beneficial for excluding significant CHD and identifying myocardial inflammatory patterns (Siripanthong et al. 2020). Endocardial myocardial biopsy (EMB) has been considered as the gold standard for the diagnosis of myocarditis for a long time, which can directly show myocardial necrosis and mononuclear cell infiltration. In some cases, EMB can also do virus isolation or nucleic acid test and autoimmune function test to find evidence of the cause of myocarditis. However, in the COVID-19 cases, this evidence is rare at present, and is mainly based on some case reports. During the current COVID-19 pandemic and related medical crisis, EMB may be inappropriate as a diagnostic tool. Some scholars believe that fulminant myocarditis may be an important clinical manifestation of the COVID-19 (Chen et al. 2020a; Hu et al. 2021). The pathophysiological mechanism of myocarditis in COVID-19 patients may be a combined role of direct viral injury (by ACE2 receptor) and the host’s hyperimmune response (Ho et al. 2020; Siripanthong et al. 2020). SARS-CoV-2 particles in myocardial tissue have been detected by reverse transcription-polymerase chain reaction (RT-PCR) in some cases (Yao et al. 2020). Animal model of viral myocarditis suggests that the intrinsic immune response is activated by the release of proinflammatory cytokines in heart injury. Colzani et al. demonstrated that inflammatory cytokines including interleukin (IL)-6, IL-1β, IL-10, tumour necrosis factor alpha (TNF-α), C-reactive protein (CRP), and neutrophil to lymphocyte ratio with a specific reduction of CD4^+^ and CD8^+^ cells were at least in part responsible for the cardiovascular damage seen in COVID-19 and characterise the downstream activated pathways in human cardiomyocytes (Colzani et al. 2024). ORF8, a unique accessory protein specific to SARS-CoV-2 has been shown to be a modulator of cytokine responses during SARS-CoV-2 infection. It has the capability to induce inflammatory responses (Móvio et al. 2024). Proteins released by cell lysis may exhibit similar characteristics to viral antigens and play a role by main histocompatibility complexes (MHCs). Myosin heavy chain, a kind of myocardial sarcomere protein, seems to be a main example of “molecular mimicry”. CD4^+^ T helper cells (Th) and cytotoxic CD8^+^ T cells mediate their responses by activating the inflammatory cascade and cytolysis. Macrophages can migrate to the injured site. In the final stage, there is recovery or low-level chronic inflammation and the occurrence of left ventricular failure (Blyszczuk 2019). It is worth noting that myocarditis occurs 10 to 15 days after the onset of COVID-19 symptoms. Based on the above observation results and experimental background, the core problem of potential treatment plans should be the degree of myocardial injury caused by virus replication, immune mediation or other mechanisms. Since acute myocardial injury begins 2 weeks after the onset of COVID-19 symptoms (Tajbakhsh et al. 2021), adaptive T-cell-mediated immunity or dysregulated innate effects or pathways may play a key role on the occurrence of myocarditis. In severe cases, the increase of highly proinflammatory CCR6^+^ Th17 in CD4^+^ T cells was the prominent inflammatory mediator of myocarditis. In this way, most scholars believe that a delay of myocardial inflammation is consistent with at least two pathogenic mechanisms: first, that the “cytokine storm” induces a subclinical autoimmune myocarditis; second, myocardial injury and/or molecular mimicry cause a new autoimmune response. To date, the targeted treatment plan for myocarditis is still difficult to implement. As for myocarditis in other cases, the strategy of widely supporting treatment is still the key measure. A recent case report showed that the early use of steroids and intravenous use of immunoglobulin, neuraminidase inhibitors and active mechanical life support had a top effect (Chen et al. 2020a; Kitsou et al. 2024).

### Hypertension

Hypertension is a common cardiovascular complication in patients with COVID-19 (Harrison et al. 2021; Pellicori et al. 2021). The prevalence of hypertension among hospitalized COVID-19 patients was 15–40% in different studies (Guan et al. 2020; Beaney et al. 2019; Mauer et al. 2022; Vosko et al. 2023). A previous analysis from China showed that hypertension was present in 13.4% of subjects with non-severe disease and in 23.7% of subjects with severe disease. A higher prevalence of hypertension was also observed in those with a poor composite outcome (Guan et al. 2020). The prevalence of hypertension was 12.8% in the whole COVID-19 patients and 39.7% in the deceased (Epidemiology Working Group for NCIP Epidemic Response, Chinese Center for Disease Control and Prevention 2020). In addition, the odds ratio (OR) of mortality in COVID-19 patients with hypertension was significantly increased by 3.05 (95% CI 1.57–5.92) (Zhou et al. 2020a). Although these evidences are insufficient to indicate an increased susceptibility of hypertensive patients to SARS-CoV-2 (Kreutz et al. 2020), the prognosis is significantly poorer, the course of the disease is more severe, and the mortality rate is higher in the elderly than in young patients (Guan et al. 2020; Su et al. 2022). The possible association between hypertension and COVID-19 may attribute to the role of ACE2 (Harrison et al. 2021). ACE2 is a key component of the RAAS, which is closely related to the pathophysiology of hypertension (Shukla and Banerjee 2021; Vaduganathan et al. 2020). Inhibition of RAAS by ACE inhibitors (ACEIs) or angiotensin receptor blockers (ARBs) can lead to a compensatory increase of ACE2 levels in tissues, indicating that these drugs may be harmful to patients exposed to SARS-CoV-2 (Danser et al. 2020). However, there is no clear evidence to suggest that ACEIs or ARBs lead to upregulation of ACE2 in human body (Danser et al. 2020). Therefore, there is no justification for stopping ACEIs or ARBs in patients at risk of COVID-19 (Sommerstein et al. 2020). As a matter of fact, the application of ACEIs/ARBs may be a double-edged sword in COVID-19 patients. On the one hand, it may increase the risk of SARS-CoV-2 infection. On the other hand, it may decrease the severity of lung damage caused by SARS-CoV-2 infection. In addition, SARS-CoV-2 infection may affect the balance between Ang II and Ang 1–7, while ACEI**s**/ARBs can block the RAAS and protect the heart and other organs, which are susceptible to damage caused by the RAAS activation (Guo et al. 2020a). The action of immune system is another mechanism linking hypertension and COVID-19. The function of immune system is dysregulated in hypertension and SARS-CoV-2 infection (Drummond et al. 2019; Loperena et al. 2018). Further dysregulation of the immune system was observed in patients with poor control of blood pressure. Blood lymphocyte counts were associated with human hypertension (Siedlinski et al. 2020), and CD8^+^ T cell dysfunction was also observed in hypertensive patients (Youn et al. 2013). CD8^+^ T cells cannot effectively fight against viral infection and lead to pathological overproduction of cytokines, which may be related to SARS-CoV-2 infection. Conversely, the dysregulated immune system in hypertension can be restored after better control of blood pressure by using ACEIs or ARBs.

### Arrhythmia

Arrhythmia is common in COVID-19 patients. It can be a new-onset arrhythmia or an aggravation of a previously existing arrhythmia which indicates myocardial involvement. Liu et al. reported that palpitation was one of their presenting symptoms in 7.3% of the COVID-19 patients (Liu et al. 2020b). Wang et al. revealed that 16.7% of 138 hospitalized COVID-19 patients with arrhythmia, which was higher in ICU patients (44.4%) than in non-ICU patients (6.9%; Wang et al. 2020a). The prevalence of malignant arrhythmia such as hemodynamically unstable ventricular tachycardia or ventricular fibrillation was also higher in patients with high troponin than those with normal troponin levels (11.5% vs. 5.2%, *P* < 0.001; Guo et al. 2020b). The main types of arrhythmia in patients with COVID-19 included atrial fibrillation, atrioventricular block, ventricular tachycardia (pleomorphism, torsade de pointes) and ventricular fibrillation (Varney et al. 2022; Kochav et al. 2020). Bhatla et al. reported that there were 9 cases of cardiac arrest, 25 cases of atrial fibrillation, 9 cases of clinically significant slow arrhythmia, and 10 cases of non persistent ventricular tachycardia among a total of 700 patients (45% male; 71% African American) with COVID-19 (Bhatla et al. 2020). Among 241 COVID-19 patients in a tertiary hospital in Brazil, the prevalence of arrhythmia was 8.7%, and the most common arrhythmia was atrial tachyarrhythmia (76.2%). The mortality rate was higher in patients with arrhythmia than without arrhythmia (52.4% vs. 24.1%, *P* = 0.005). A high risk of arrhythmia was observed in patients with heart failure (hazard ratio 11.9, 95% CI 3.6–39.5, *P* < 0.001), and 3.3% of the patients experienced cardiac arrest and died during hospitalization (Pimentel et al. 2021). This indicates that the mortality rate of cardiac arrest in COVID-19 patients was very high. Interestingly, in a previous report from China, some patients mainly manifested as cardiovascular symptoms, such as palpitations and chest tightness, rather than respiratory symptoms during the initial epidemic period (Zheng et al. 2020). The potential mechanisms of arrhythmias in COVID-19 patients have not been fully elucidated. All of SARS-CoV-2 infection-related metabolic dysfunction, myocardial inflammation, and activation of the sympathetic nervous system were associated with cardiac arrhythmia (Su et al. 2022). It is established that the immune system is implicated in the pathogenesis of arrhythmias. Auto-immune and inflammatory cardiac channelopathies may promote arrhythmias via auto-antibodies and cytokines respectively (Lazzerini et al. 2019). Inflammatory cytokines, such as TNF-α, IL-1, and IL-6 can be arrhythmogenic and this phenomenon is observed after a systemic inflammatory response to a pathogen, including SARS-CoV-2 (Tsampasian et al. 2024). The concentration of TNF-α, IL-1, and IL-6 in patients with long COVID was substantially elevated for prolonged periods (Phetsouphanh et al. 2022; Schultheiß et al. 2022; Karbalaeimahdi et al. 2023; Melhorn et al. 2023). Thus, the possible risk factors include hypoxia, myocarditis, abnormal host immune response, myocardial ischemia, myocardial strain, electrolyte disorder, intravascular volume imbalance, metabolic disarray, sympathetic nervous system activation, hypotension, and drug side effects such as COVID-19 drug therapy and other drug interactions. It is worth noting that some drugs for COVID-19 therapies can prolong the QT interval and may have arrhythmogenic effects (Manolis et al. 2020; Kochi et al. 2020; Dherange et al. 2020; Yu et al. 2024).

### Myocardial injury and heart failure

Myocardial injury in COVID-19 patients is commonly associated with disease severity. A number of previous studies showed that the serum concentrations of lactate dehydrogenase (LDH), creatine kinase (CK) and its isoenzyme CK-MB, and high-sensitivity cardiac troponin (hs-cTn) were increased in almost all hospitalized COVID-19 patients (Zheng et al. 2020; Huang et al. 2020; Lippi et al. 2020), or had evidence of new electrocardiographic or echocardiographic abnormalities (Huang et al. 2020; Zhou et al. 2020a). Approximately 10% of COVID-19 patients had heart failure, with incidence ranging from 25 to 35% in hospitalized patients (Shu et al. 2023). Heart failure was also observed in 52% of non survivors and 12% of survivors. A previous report from Wuhan, China showed that five of the first 41 patients (12%) with COVID-19 had the evidence of myocardial injury, such as elevated high-sensitivity cardiac troponin I (cTnI, > 28 pg/mL) levels (Zheng et al. 2020; Huang et al. 2020; Lippi et al. 2020), and 7.2–17% of COVID-19 inpatients had acute myocardial injury (Wang et al. 2020a; Zhou et al. 2020a). In another analysis of 68 death causes from Wuhan, 36 cases (53%) were respiratory failure, 5 cases (7%) were myocardial injury and circulatory failure, 22 cases (33%) were both respiratory and circulatory failures, and 5 cases (7%) were unknown cause (Ruan et al. 2020). In addition, the level of N-terminal pro B-type natriuretic peptide (NT proBNP) in COVID-19 patients was also increased in 27.5% of cases. In a previous report of 138 COVID-19 inpatients from Wuhan, patients treated in ICU had higher levels of biomarkers (CK-MB and hs-cTnI) of myocardial injury than those do not need ICU care (Wang et al. 2020a). In the study conducted by Zhou et al. univariate analysis showed that cTnI level was closely related to increased mortality, but this correlation could not be detected in a multivariate model (Zhou et al. 2020a). When the cohorts were analyzed according to the need for ICU care, the similar correlation between elevated cTnI and the severity of the disease was also found (Wang et al. 2020a; Huang et al. 2020). Moreover, recovered COVID-19 patients showed an increased risk of incident heart failure in the same follow-up period. COVID-19 survivors had an additional 90% risk of developing heart failure after COVID-19 infection in the long-term period. This risk was directly related with age and previous history of hypertension especially in the early post-acute phase of the infection (Zuin et al. 2023b).

Cardiogenic shock is a critical manifestation of myocardial injury in COVID-19 patients (Shu et al. 2023). The incidence of cardiogenic shock in COVID-19 was about 0.7%. Compared to patients without shock, those with cardiogenic shock had a higher incidence of previous myocardial infarction, coronary revascularization, and heart failure, as well as abnormal chest imaging and elevated troponin, D-dimer, CRP, and natriuretic peptides on admission. The incidence of in-hospital mortality, cardiac arrest, myocardial infarction, or stroke were higher in patients with cardiogenic shock than in those without cardiogenic shock (77% vs. 13%; Varshney et al. 2021). At present, it is believed that large-scale cytokine storm induced by viral infection is the major cause of cardiogenic shock. Moderate doses of steroids can significantly improve the shock and multiple organ dysfunction of the patients.

At present, the exact mechanism of myocardial injury in COVID-19 patients is not fully understood. The proposed mechanisms of myocardial injury include direct damage effects of SARS-CoV-2, systemic inflammation, endothelial dysfunction, platelet activation, sympathetic activation, myocardial interstitial fibrosis, IFN mediated immune response, exaggerated cytokine response by types 1 and 2 helper T cells, vasoconstriction, hypercoagulation, hypoxemia, and COVID-19 therapy-related drugs such as corticosteroids or rivabirina (Wang et al. 2020a; Shi et al. 2020; Guo et al. 2020b; Zhou et al. 2020a; Chen et al. 2020b; Babapoor-Farrokhran et al. 2020; Xu et al. 2020b; Varga et al. 2020; Klok et al. 2020). Due to the high expression of ACE2 in the cardiovascular system, especially in the failed human heart (Chen et al. 2020b), SARS CoV-2 may directly infect myocardial cells, which seems to explain the higher viral infection and mortality rates in heart failure patients. The high inflammatory state and cytokine release induced by virus infection may lead to vascular and myocardial inflammation, plaque instability, hypercoagulability, and even directly inhibit the myocardium. In this case, the “cytokine storm” caused by immune imbalance may be a key modulator (Zheng et al. 2020). The concentration of plasma IL-6 in COVID-19 patients with myocardial injury was increased (Chen et al. 2020a), and there were many abnormal cytokines in COVID-19 patients. Both TNF-α and IL-6 are known to be implicated in the pathophysiology of myocardial infarction, inflammation, and heart failure (Schumacher and Naga Prasad 2018; Hanna and Frangogiannis 2020). Other complications such as sepsis and disseminated intravascular coagulation (DIC) may also lead to myocardial injury. In addition, patients with long COVID have been shown to have auto-antibodies specifically against components of the cardiovascular system, including anti-cardiolipin and anti-apolipoprotein A1 antibodies, both of which are linked with cardiovascular events and worse outcomes (Dobrowolska et al. 2023).

Endothelial cell dysfunction is closely associated with the development of many cardiovascular diseases, such as atherosclerosis, CHD, hypertension and heart failure. Numerous studies have shown that SARS CoV-2 infection can cause serious cardiovascular system complications. The patients with pre-existing cardiac disease have a higher incidence of adverse events and mortality after SARS CoV-2 infection. Endothelial damage may be an important part of the pathogenesis (Rossouw et al. 2022). Histological and pathological findings of patients who died from COVID-19 suggested the presence of endothelial inflammation, degradation of endothelial cells, and viral structures in endothelial cells were observed in multiple organs throughout the body (Fodor et al. 2021). The pathological mechanism of endothelial cell structure and dysfunction caused by SARS-CoV-2 is complex. First, the virus itself attacks and damages endothelial cells. S protein disrupts endothelial cell integrity, and N protein induces a pro-inflammatory cell phenotype that triggers the release of inflammatory factors and cytokines by binding to TLR2 in endothelial cells, triggering the NF-κB and MAPK signaling pathways (Qian et al. 2021). Both of these proteins drive viral-mediated endothelial injury. In addition, S protein can activate the alternative pathway of complement to increase endothelial cytotoxicity and activate pyrin domain-containing 3 (NLRP3) present in vascular endothelial cells, leading to endothelial cell dysfunction (Rossouw et al. 2022). SARS-CoV-2 can also indirectly damage endothelial cells through oxidative stress (Fodor et al. 2021). SARS-CoV-2 can induce activation of nicotinamide adenine dinucleotide phosphate (NADPH)-oxidase and promote superoxide (O_2_^−^) production, which leads to mitochondrial damage (Fodor et al. 2021). Damaged mitochondria in COVID-19 patients can promote β-oxidation of fatty acids in vascular endothelial cells to increase oxidative stress (Montiel et al. 2022). In addition, oxidative stress can promote the oxidation of thiols in SARS-CoV-2 and SARS-CoV-2 proteins to disulfides, increasing viral binding to ACE2 and thus aggravating the infection (Hati and Bhattacharyya 2020). Therefore, the reduction of oxidative stress is an essential component for the intervention and control of the recent and long-term complications of SARS-CoV-2 infection. Severe SARS-CoV-2 infection can increase expression of adhesion molecules and platelet aggregation, leading to endothelial cell damage (Rossouw et al. 2022). It was found that some patients with SARS-CoV-2 infection have different types of antibodies in their sera that activate endothelial cells to increase the expression of surface adhesion molecules such as intercellular adhesion molecule-1, E-selectin, and vascular cell adhesion molecule-1, increasing the incidence of adverse thrombotic events (Shi et al. 2022). Meanwhile, TLR7 on the platelet surface during SARS-CoV-2 infection binds to the single-stranded RNA of SAR2-CoV-2, accelerating endothelial damage and leading to increased thrombotic susceptibility (Rossouw et al. 2022). Endothelial cell injury can cause excessive platelet stress, and the interaction between the two disrupts the pre-existing homeostatic balance of the vasculature, thereby causing microvascular occlusion, cellular oxidative stress, and the release of pro-thrombotic/pro-coagulant factors (Rossouw et al. 2022). In addition, endothelial cell dysfunction can be secondary to inflammatory responses and increased vascular permeability, leading to the development of myocardial edema and myocarditis (Prasad et al. 2021; Rossouw et al. 2022).

Currently, the treatment measures for myocardial injury caused by SARS CoV-2 include steroids, immunoglobulin, hydroxychloroquine and other antiviral drugs, as well as various life support therapies (Chen et al. 2020a). Although it is uncertain whether these treatments can successfully limit myocardial injury, the detection of cardiac injury indicators in hospitalized patients with COVID-19 may help to determine the risk of complications.

### Coronary heart disease

SARS CoV-2 infection has been associated with a higher incidence of acute myocardial infarction and related complications (Nanavaty et al. 2024). COVID-19 is a risk factor for acute myocardial infarction, which may represent a part of the clinical picture of COVID-19 (Katsoularis et al. 2021). ST-segment elevation myocardial infarction (STEMI) is one of the fatal complications following COVID-19 (Gharibzadeh et al. 2023). According to available data, 2.5–15% of COVID-19 patients had CHD (Wang et al. 2020a; Chen et al. 2020c; Guan et al. 2020). In a previous analysis of 191 patients, 15 (8%) of COVID-19 patients had CHD (Zhou et al. 2020a). Recent studies and meta-analysis revealed that COVID-19 patients with CHD were associated with poor prognosis (Liang et al. 2021; Gharibzadeh et al. 2023; Majeed et al. 2023; Baytuğan et al. 2024; Dogan et al. 2024). Majeed et al. revealed that STEMI patients with COVID-19 had higher inpatient mortality, increased length of stay and higher cost of hospitalization when compared to STEMI patients without COVID-19. STEMI patients with COVID-19 also received significantly less invasive cardiac procedures such as coronary angiograms, percutaneous coronary intervention, and coronary artery bypass grafting and were more likely to receive systemic thrombolytic therapy when compared to STEMI patients without COVID-19 (Majeed et al. 2023). The mortality and adverse consequences of STEMI patients with COVID-19 were also far higher than in the general population (Gharibzadeh et al. 2023). STEMI patients with concomitant COVID-19 or with a history of SARS CoV-2 infection were associated with increased major adverse cardiac events (Dogan et al. 2024; Baytuğan et al. 2024). These results suggest that CHD is a risk factor for poor prognosis of COVID-19 patients. Currently, the mechanisms of SARS CoV-2 infection induced acute myocardial infarction, especially non-ST-T elevation myocardial infarction (NSTEMI) include: (1) SARS CoV-2 infection can cause systemic inflammatory response syndrome, cytokine storm and immune response that increase the risk of plaque rupture and thrombus formation, resulting in either an ST-elevation or non-ST-elevation myocardial infarction (Kang et al. 2020; Musher et al. 2019; Cole et al. 2018). (2) SARS CoV-2 infection can also reduce the oxygen delivery to myocardium via hypoxemia and vasoconstriction, as well as the hemodynamic effects of sepsis with increased myocardial oxygen demand. This supply-demand mismatch may lead to sustained myocardial ischemia in patients with underlying CHD (Kang et al. 2020). (3) DIC was found in 0.6% of survivors and 71.4% of non-survivors with COVID-19 (Tang et al. 2020). DIC was associated with coronary artery (epicardial and microvascular) thrombosis, focal myocardial necrosis, and severe cardiac dysfunction (Wang et al. 2020c; Hakobyan et al. 2023). (4) Myocardial injury of COVID-19 may also occur through non-ischaemic mechanisms, such as acute and fulminant myocarditis and stress cardiomyopathy (Zeng et al. 2020; Fried et al. 2020; Sala et al. 2020; Arentz et al. 2020; Inciardi et al. 2020). (5) SARS-CoV-2 can cause the immune system to strongly release various cytokines and chemokines (IL-1, IL-6, T helper 1 cytokine IFN-γ, and TNF-α; cytokine release syndrome or cytokine storm). These proinflammatory cytokines may immediately inhibit myocardial function by activating the neural sphingomyelinase pathway (Ruan et al. 2020; Huang et al. 2020; Mehta et al. 2020; Mann 2015). Besides, cytokine storm may lead to extensive endothelial dysfunction, serious microvascular dysfunction, and induce non obstructive acute myocardial infarction (Chen et al. 2020b).

### Stress cardiomyopathy

Stress cardiomyopathy (Takotsubo syndrome) is a life-threatening transient left ventricular dysfunction triggered by either physical or emotional stressors (Singh et al. 2023). It has become a well-known complication of SARS-CoV-2 infection (Davis et al. 2023). During COVID-19 pandemic, emotional and physical distress indued by strict social distancing rules, self-isolation, quarantine, economic and social stress, fear of virus infection may be the cause to trigger the takotsubo syndrome (Burger et al. 2021). A significant increase in the incidence rate of takotsubo syndrome has been found dring COVID-19 pandemic (Hajra et al. 2023). Hajra et al. found that the in-hospital outcomes in patients with stress cardiomyopathy and concurrent SARS-CoV-2 infection with those without SARS-CoV-2 infection were significantly different. In a total of 41,290 hospitalizations for stress cardiomyopathy (1665 patients with COVID-19), the incidence of complications, including acute kidney injury, acute kidney injury requiring dialysis, coagulopathy, sepsis, cardiogenic shock, cases with prolonged intubation of > 24 h, requirement of vasopressor and inpatient mortality, were significantly higher in patients with COVID-19. Concomitant COVID-19 infection was independently associated with worse outcomes and increased mortality in hospitalized patients with stress cardiomyopathy (Hajra et al. 2023). COVID-19-related stress cardiomyopathy may manifest as cardiogenic shock (Shu et al. 2023). In a total of 1,659,040 patients, there were 1,665 COVID-19 patients with stress cardiomyopathy (0.1%) and 1,657,375 COVID-19 patients without stress cardiomyopathy (99.9%). COVID-19 patients with stress cardiomyopathy had significantly increased in-hospital mortality compared to COVID-19 patients without stress cardiomyopathy (32.8% vs. 14.6%, *P* = 0.01) along with significantly increased mechanical ventilation and vasopressor support, hospitalization charge, acute kidney injury requiring hemodialysis, cardiogenic shock, and cardiac arrest. These results emphasize the need for more research to reduce worse outcomes of patients with COVID-19-related stress cardiomyopathy (Davis et al. 2023).

### Ischemic stroke

There is increasing evidence that COVID-19 is associated with ischemic stroke (Finsterer et al. 2022; Ozcan et al. 2023; De Michele et al. 2023). An increasing number of COVID-19 patients with ischemic stroke have been reported (Luo et al. 2022; Zuin et al. 2023a). A systematic review and meta-analysis including 26,691 participants and 280 patients with ischemic stroke and COVID-19 showed that the pooled prevalence of ischemic stroke in COVID-19 was 2% (95% CI 1–2%; *P* < 0.01). The pooled proportions of hypertension, hyperlipidemia and diabetes in COVID-19-related ischemic stroke was 66%, 48% and 40% (*P* < 0.01 for all), respectively (Luo et al. 2022). Another systematic review and meta-analysis including 23,559,428 patients (1,595,984 COVID-19 patients) revealed that ischemic stroke occurred in 4.40 out of 1000 patients survived to COVID-19 compared to 3.25 out of 1000 controls (Over a mean follow-up of 9.2 months). Recovered COVID-19 patients presented a higher risk of ischemic stroke compared to people who did not have COVID-19. COVID-19 patients hospitalized at the time of the infection have a subsequent higher risk of stroke during the follow-up compared to those non-hospitalized (Zuin et al. 2023a). Moreover, a recent study demonstrated that COVID-19 extends the infarct volume during acute ischemic stroke (De Michele et al. 2023). COVID-19-related ischemic stroke may occur in all age groups and predominantly in males. The anterior circulation is more frequently affected than the posterior circulation (Finsterer et al. 2022). The mechanisms underlying ischemic stroke are thought to be driven by multiple pathophysiological factors, including hypercoagulation, microthrombosis, and endothelial dysfunction (De Michele et al. 2023). Immune-mediated thrombosis, the RAAS and the effect of SARS-CoV-2 in cardiac and brain tissue may also contribute to the pathogenesis of ischemic stroke in patients with COVID-19 (Sagris et al. 2021). In addition, certain studies have suggested that COVID-19 induces acute ischemic stroke by promoting hypercoagulability. These patients often had an abnormal coagulation, namely, elevated levels of D-dimer and fibrinogen, and a low platelet count. Nevertheless, the exact mechanisms through which COVID-19 leads to a hypercoagulable state in infected patients remain unclear (Zhang et al. 2021).

### Blood coagulation abnormalities

COVID-19 is also associated with thromboembolic disease, and increases the risk of venous and arterial thromboembolism events (Tomasoni et al. 2020; Zhou et al. 2020a; Tang et al. 2020; Ali and Spinler 2021; Danzi et al. 2020; Wichmann et al. 2020; Wichmann 2020; Gąsecka et al. 2021; Kyriakoulis et al. 2021; Heinrich et al. 2022; Marvi et al. 2022; Tsaplin et al. 2021; Farkouh et al. 2022; Epelbaum 2020; Takasu et al. 2022; Hendren et al. 2020). Both DIC and pulmonary embolism were very common in COVID-19 patients. DIC was found in 71.4% of non survivors among COVID-19 patients (Tang et al. 2020). A large number of pulmonary embolisms were also reported in COVID-19 patients (Danzi et al. 2020; Hobohm et al. 2023). In Germany, the fatality rate among patients with both COVID-19 and pulmonary embolism was substantially higher than that in those with only one of these diseases, suggesting a life-threatening additive prognostic impact of the COVID-19-pulmonary embolism combination (Hobohm et al. 2023). The incidence of deep venous thrombosis in COVID-19 patients was 22.7% by ultrasound examination (Shi and Fu 2020) and 27% in ICU patients (Klok et al. 2020). A previous autopsy report by German scholars showed that 7 of the 12 patients (58%) had deep vein thrombosis, which was not suspected before death. The direct cause of death in four patients was pulmonary embolism. Reticular infiltration of the lungs with severe bilateral dense consolidation was found by autopsy computed tomography scan, while 8 patients had histomorphologically diffuse alveolar injury. High concentration of SARS-CoV-2 RNA was detected in the lungs, and high viral RNA titers in the liver, kidney, or heart were also determined in 6 of 10 and 5 of 12 patients (Wichmann et al. 2020; Wichmann 2020). Gąsecka et al. revealed that 1 of 3 inpatients with severe COVID-19 had macrovascular thromboembolic complications, including venous thromboembolism, myocardial injury/infarction, and stroke. Meanwhile, the autopsy series showed consistent patterns of multiple organ damage and microvascular damage (Gąsecka et al. 2021). Microthrombosis mainly occurs in the pulmonary vascular system, but it may also occur in other organs (Kyriakoulis et al. 2021). Heinrich et al. also showed that venous thromboembolism in critical COVID-19 patients was 17% of ante mortem and 38% of postmortem. Incidence rate of postmortem venous thromboembolism was higher in COVID-19 (43%) than in age- and sex-matched non-COVID-19 (0%) cohorts (*P* = 0.001; Heinrich et al. 2022). In a recent study, Marvi et al. found that the maximum amplitude of thromboelastogram was related to the occurrence of venous thromboembolism in critical COVID-19 patients. For every 1 mm increase in enrollment and peak maximum amplitude, the risk of venous thromboembolism was reduced by 8% and 14%, respectively. Lower enrollment platelet counts and fibrinogen levels were also associated with an increased risk of venous thromboembolism. Platelet counts and fibrinogen levels were positively associated with maximum amplitude. The association between diminished maximum amplitude, platelet counts, fibrinogen and venous thromboembolism may suggest a relative consumptive coagulopathy in critical COVID-19 patients (Marvi et al. 2022). Recently, the study by Tsaplin et al. found that there was a significant correlation between the Caprini score and the risk of venous thromboembolism in COVID-19 patients. All of eight models (eight different versions) including specific COVID-19 scores showed equally high predictability, and use of the original Caprini score was appropriate for COVID-19 patients (Tsaplin et al. 2021). These results indicate that coagulation abnormality induced by COVID-19 plays an important role in the high incidence of thromboembolic events. Clinical experience showed that coagulopathy associated with COVID-19 had obvious characteristics, including markedly elevated D-dimers concentration (Gąsecka et al. 2021), which greatly predicted the adverse consequences of COVID-19. A retrospective and multicenter cohort study showed that elevated levels of D-dimer (> 1 µg/L) were closely related to hospitalization mortality rate (Zhou et al. 2020a). Heinrich et al. showed that the change of anticoagulation practice was related to the statistically significant prolongation of survival time (HR = 2.55, 95%CI = 1.41–4.61, *P* = 0.01) and reduced the occurrence of venous thromboembolism (54% vs. 25%; *P* = 0.02; Heinrich et al. 2022). Some studies also support that anticoagulant therapy may play a role in patients who do not need ICU support (Gąsecka et al. 2021; Kyriakoulis et al. 2021; Farkouh et al. 2022). However, due to the lack of guidance on determining the strength and duration of anticoagulation, decisions should be made based on specific circumstances (Gąsecka et al. 2021). At present, the pathophysiolocal mechanisms of blood coagulation abnormalities in COVID-19 patients have not been fully explored. The independent risk factors for venous thromboembolism include increasing age, males, long interval from symptom onset to admission, low fibrinogen, increased activity of factor V, high D-dimer levels on admission, and D-dimer increment ≥ 1.5 times (Wu et al. 2021; Shu et al. 2023). Endothelial injury that induces tissue factor and platelet activation, low fibrinolysis, and pro-inflammatory cytokines that promote microvascular damage have been implicated in the thrombotic process, and involved in the development of venous thromboembolism in COVID-19 patients (Shu et al. 2023). The high inflammatory load associated with COVID-19 seems to be associated with coexisting coagulopathy (Kyriakoulis et al. 2021). Inflammation may occur in endothelial cells. Significant inflammation with endotheliitis can also lead to DIC, thrombosis of small vessels or great vessels with tissue necrosis or infarction (Tomasoni et al. 2020; Hendren et al. 2020).

### Dyslipidemia

SARS-COV-2 infection is linked with the development of cardio-metabolic disorders, including dyslipidemia (Al-Kuraishy et al. 2023). Cholesterol may play a central role of the SARS-COV-2 infection. An elevated cholesterol concentration has been suspected to increase the susceptibility for SARS-COV-2 infection. Conversely, higher high-density lipoprotein cholesterol (HDL-C) levels seem to have protective action (Julius et al. 2022). Higher antecedent serum HDL-C levels were associated with a lower SARS-CoV-2 infection risk (Chidambaram et al. 2022a). Lower HDL-C levels correspond with a higher susceptibility to SARS-CoV-2 infection in general, while higher HDL-C levels were related to a lower risk of SARS-CoV-2 infection (Kowalska et al. 2022). Elevated triglyceride (TG) levels in COVID-19 patients may be considered an indicator of uncontrolled inflammation and an increased risk of death because TG levels were significantly higher in non-surviving severe patients than in surviving mild patients (Kowalska et al. 2022). However, the changes in serum lipid levels are inconsistent in COVID-19 patients. The levels of total cholesterol (TC) were significantly lower in COVID-19 patients than in healthy controls (Chidambaram et al. 2022b; Wang et al. 2020b). The concentrations of TG were lower (Wang et al. 2020b) or higher (Julius et al. 2022; Kowalska et al. 2022) in COVID-19 patients than in healthy controls, but did not differ based on COVID-19 severity or mortality (Chidambaram et al. 2022b). Serum HDL-C (Chidambaram et al. 2022b; Julius et al. 2022; Kowalska et al. 2022; Wang et al. 2020b) and LDL-C (Chidambaram et al. 2022a; Kowalska et al. 2022; Wang et al. 2020b) levels were significantly lower in COVID-19 patients than in healthy controls. Lipoprotein (a) may be increased during SARS CoV-2 infection and is most probably responsible for thromboembolic events. This lipoprotein can induce a progression of atherosclerotic lesion formation (Julius et al. 2022). A decrease in apolipoprotein A1 was associated with increased clinical severity in COVID-19 (Mietus-Snyder et al. 2022). In addition, other lipid particles, including total, large, and small HDL particles, as well as HDL functional cholesterol efflux capacity, were related to the severity of COVID-19 among pediatric patients (Mietus-Snyder et al. 2022). Severe COVID-19 patients had lower TC, LDL-C, and HDL-C at admission compared to patients with non-severe disease. Deceased patients had lower TC, LDL-C and HDL-C at admission (Chidambaram et al. 2022b). Wang et al. also found lower levels of HDL-C in severe COVID-19 patients than in non-severe patients. Moreover, patients with low HDL-C at admission showed a higher risk of developing severe events compared with those with high HDL-C (Wang et al. 2020b). A direct correlation was found between a decrease in serum cholesterol, HDL-C, LDL-C and TG concentrations and the severity of the disease. These laboratory findings may serve as potential markers for patient outcomes (Kowalska et al. 2022). The pathogenic mechanisms of dyslipidemia in COVID-19 patients are not fully elucidated. The possible mechanisms are as follows: (1) Cholesterol is necessary for SARS-CoV-2 to enter the host cells, and membrane cholesterol increases the number of viral entry sites on the host cell membrane and the number of ACE2 receptors in the membrane fusion site (Tang et al. 2021). SARS-CoV-2 binds HDL-C, and then this complex is attached to the co-localized receptors, facilitating viral entry (Al-Kuraishy et al. 2023). (2) SARS-CoV-2 infection may induce the development of dysfunctional HDL-C through different mechanisms, including induction of inflammatory and oxidative stress with activation of inflammatory signaling pathways. In turn, the induction of dysfunctional HDL-C induces the activation of inflammatory signaling pathways and oxidative stress, increasing COVID-19 severity (Al-Kuraishy et al. 2023). (3) Cholesterol in blood interacts with S protein to promote the entry of spike cells, wherein the scavenger receptor class B type 1 plays an important role (Tang et al. 2021). (4) *APOE* binding to ACE2 attenuates the interaction of ACE2 with S protein, inhibits SARS-CoV-2 pseudovirus infection, and attenuates the inflammatory response. However, the inhibitory effect of *APOE4* was lower due to different conformational structures (Zhang et al. 2022). (5) Patients carrying the *APOEε4* gene have a higher susceptibility to SARS-CoV-2 and increased serum inflammatory factors (Zhang et al. 2022). Because of the cardiovascular protective effects and the additional anti-inflammatory effects of lipid-lowering drugs, it is currently recommended to continue lipid-lowering therapy for COVID-19 patients (Tang et al. 2021; Julius et al. 2022). Statins, the 3-hydroxy-3-methylglutaryl coenzyme A reductase (HMG-CoA) inhibitors, have cholesterol-lowering, anti-inflammatory, antithrombotic, and antioxidant effects. Kouhpeikar et al. demonstrated that statins decreased the composite outcomes of mortality, ICU admissions, and intubations among COVID-19 patients (Kouhpeikar et al. 2022). Statin treatment also lowered inflammatory markers such as CRP levels and neutrophil counts. These findings suggest a potential antiinflammatory role of statins in mitigating the composite adverse outcomes associated with COVID-19 (Ozcan et al. 2023). Preclinical and clinical studies indicated a potential therapeutic role of apolipoproteins and agents targeting them in COVID-19. One of the potential mechanistic hallmarks underlying the benefits of apolipoproteins is suggested to be protection against COVID-19-induced endothelial dysfunction (Ozcan et al. 2023). Some scholars, however, believe that lipid-lowering treatment should be carried out with caution, as plasma LDL-C levels may have a dual impact on COVID-19 patients, similar to a double-edged sword (Ozcan et al. 2023).

## Therapy strategies of COVID-19

There are currently no specific treatment methods for the SARS-CoV-2 infection, although many are under investigation. Early diagnosis of SARS CoV-2 infection is crucial for the recommendation of appropriate treatment strategy and to address associated cardiovascular complications. To minimize these complications in COVID-19 patients, the COVID-19 patients require routine monitoring of cardiac parameters with echocardiography, telemetry to assess QT interval and electrocardiograph to identify the occurrence of cardiovascular complications (Samidurai and Das 2020). In addition, treatment of COVID-19 should be personalized according to host characteristics, degree of severity and available treatment options (Lui and Guaraldi 2023). The proposed therapy approaches are summarized as follows (Lu 2020a; Morse et al. 2020).

### Supportive therapies

Oxygen therapy is the choice for patients with severe respiratory infections, respiratory distress, hypoxemia or shock. Respiratory support should be given to patients with hypoxic respiratory failure and acute respiratory distress syndrome. Extracorporeal membrane oxygenation (ECMO) should be considered for the patients with refractory hypoxemia that is difficult to be corrected by protective lung ventilation (Jin et al. 2020). Cardiogenic shock due to fulminant myocarditis, acute myocardial infarction and others can implant an intra-aortic balloon pump (IABP; Jokšić-Mazinjanin et al. 2023; Mirza et al. 2023).

### Antiplatlet and anticoagulant therapies

All P2Y_12_ receptor inhibitors can reduce platelet-leukocyte aggregates and platelet-derived pro-inflammatory cytokines. Evidences suggested that prehospital use of aspirin was associated with lower risk of developing acute respiratory distress syndrome and mortality in pneumonia patients. Ticagrelor has potent anti-inflammatory properties via dual inhibition of platelet P2Y_12_ receptor and equilibrative nucleoside transporter 1, which inhibits cellular adenosine uptake. Ticagrelor also has encouraging clinical benefit in the management of pneumonia by preventing the complications of sepsis and reducing lung injury (Su et al. 2020). Zhou et al. suggested among COVID-19 patients who are currently on antiplatelet therapy, maintaining P2Y_12_ inhibitor monotherapy such as ticagrelor may be scientifically reasonable for the patients with percutaneous coronary intervention performed ≥ 3 months (Zhou et al. 2020b; Su et al. 2020). Blood coagulation abnormalities such as venous and arterial thromboembolism, pulmonary embolism, DIC, and microvascular thrombosis/occlusion are known to occur in the majority of COVID-19 patients. Therefore, anticoagulant therapy (unfractionated heparin or low molecular weight heparin) in some COVID-19 patients is necessary. Anticoagulant therapy with direct oral anticoagulation was proven to be effective in reducing the mortality risk in patients with myocardial injury in patients after non-cardiac surgery, but the effect on the myocardial damage in COVID-19 is still unexplored (Nuzzi et al. 2022). In a study including 449 severe COVID-19 patients, anticoagulant therapy using low molecular weight heparin was associated with lower mortality in the subpopulation meeting sepsis-induced coagulopathy criteria or with markedly elevated D-dimer. These findings suggest that all hospitalized COVID-19 patients should receive thromboprophylaxis, or full therapeutic-intensity anticoagulation if indication is present (Bikdeli et al. 2020; Barrett et al. 2020).

### Immunotherapy

Immunotherapy includes immunesuppression and immunomodulation. There are many kinds of drugs such as glucocorticoids (prednisone, hydrocortisone and methylprednisolone); IL-6 receptor inhibitors [tocilizumab (an antihuman IL-6 receptor monoclonal antibody), sarilumab and siltuximab]; convalescent serum or plasma (viral neutralization, specific immunoglobulin G antibody); IFN-β (Has immunomodulatory properties); cyclosporine, azathioprine; and immunomodulation (intravenous immunoglobulins, monoclonal antibodies targeting IL-6 or IL-6 receptor). Vaccination has been shown to reduce the risk of cardiac injury (Parodi et al. 2023) and prevent long COVID syndrome (Antonelli et al. 2022). A meta-analysis has already shown that vaccinated individuals have 40% less risk to develop long COVID compared to unvaccinated people (Tsampasian et al. 2023). Another systematic review and meta-analysis of six studies and 629,093 patients showed that patients with two-dose vaccination had 36% and 40% less risk of long COVID compared to those with no or one-dose vaccination (Watanabe et al. 2023). Convalescent plasma therapy (plasma containing the antibody from recovered patients infected with COVID-19) and monoclonal antibody therapy have been evaluated with some moderate success. The results showed that convalescent plasma therapy was effective, and the level of neutralizing increased as high as 1:640 times in patients with SARS-CoV-2 infection. Monoclonal antibody can target the specific epitope on the S protein of SARS-CoV-2 and block the virus entry in to the host cells (Hoffmann et al. 2020; Samidurai and Das 2020).

### Antiretroviral therapy

There are many antiretroviral drugs, including atazanavir (inhibited SARS-CoV-2 replication and proinflammatory cytokines), liponovir/ritonavir (a combination drug also called Kaletra, to bind SARS-CoV-2 3 C-like proteinase and consequently suppress its replication), remdesivir (reducing viral replication and binding to the active site on RNA polymerase of SARS-CoV-2), ivermectin (causes an influx of Cl ions through the cell membrane, leading to hyperpolarization of ion channels and muscle paralysis), lopinavir, oseltamivir, arbidol, favipiravir (competitive inhibition of the RNA-dependent RNA polymerase), ribavirin (an RNA-dependent RNA polymerase inhibitor), nirmatrelvir (inhibits viral replication by targeting the chymotrypsin-like cysteine protease enzyme), molnupiravir, camostat mesylate (serine protease inhibitor, inhibit SARS-CoV entry into cells), chloroquine (antimalarial drug), hydroxychloroquine (rheumatoid arthritis and systemic lupus erythematosus treatment), and azithromycin (Tsampasian et al. 2024; Adu-Amankwaah 2024). Antivirals that are recommended for the acute COVID-19 infection in patients with high-risk features have also been shown to be beneficial (Xie et al. 2023b, d; Wan et al. 2023; Butler et al. 2023). A recent retrospective cohort study including 281,793 participants, showed that nirmatrelvir reduced the risk of long COVID syndrome by 26% and the risk of post-acute death and hospitalization by 47% and 24%, respectively (Xie et al. 2023c; Tsampasian et al. 2024). Large cohort studies also demonstrated that the use of nirmatrelvir and molnupiravir during the acute illness significantly reduced the incidence of long COVID syndrome and the post-acute COVID-19 sequalae (Fung et al. 2023; Xie et al. 2023a).

### Cell-based therapies

Some clinical studies support the notion that cell-based therapies of heart disease can attenuate inflammation, which may be attractive in COVID-19 (Marbán 2018). These active cells or stem cells include skeletal myoblasts, bone marrow mononuclear cells, mesenchymal stem cells (MSCs, are somatic progenitor cells that possess immunomodulatory properties), mesenchymal precursor cells, CD34^+^ cells, cardiopoietic cells, and cardiosphere-derived cells (CDCs, are stromal progenitor cells; Leng et al. 2020; Liang et al. 2020). Recently, stem cell therapies with secreted extracellular vesicles offer a potential therapeutic benefit in COVID-19 patients by attenuating inflammation with regeneration of the damaged lungs. Mesenchymal stem cells (MSCs)-derived extracellular vesicles-based therapy could be the most promising reparative strategy in people with COVID-19, because of its high proliferation rate, low invasive nature, and the immunomodulatory, antioxidant and anti-inflammatory properties of MSCs (Samidurai and Das 2020).

### Application of ACEIs/ARBs

Theoretically, ACE2 levels are increased following treatment with ACE inhibitors (ACEIs) and ARBs, which yield the concerns that using these medications might increase the severity of COVID-19, especially in patients with existing cardiovascular diseases. However, the experimental and clinical data showed conflicting results. A meta-analysis revealed that continuous administration of ACEI/ARB, compared with discontinuation, significantly reduced in-hospital mortality among hypertension patients with COVID-19 infection. Meta-regression analyses indicated a clear association between the use of antihypertensive agents and reduced mortality in these patients (Liu et al. 2024). Another study also showed that antihypertensive therapy with ACEIs/ARBs might reduce the incidence of exacerbation and in-hospital mortality (Zhang et al. 2023). However, ACEI/ARB drugs may put COVID-19 patients at high risk for moderate to severe forms of COVID-19 and higher length of hospital stay. Although, it is notable that these drugs did not significantly affect specific adverse outcomes of COVID-19, such as the need for admission to the ICU, length of ICU stay, ventilation, and mortality (Najafi et al. 2023). Currently, there is no evidence either from clinical or animal studies showing that the use of ACEI/ARB can increase cardiovascular complications. Therefore, it is recommended to continue the use of antihypertensive agents for patients with hypertension during SARS-CoV-2 infection (Su et al. 2020; Liu et al. 2024), especially in patients aged 80 years or older with hypertension (Zhang et al. 2023).

### Chinese traditional medicine

Since the outbreak of COVID-19 in China, traditional Chinese medicine (TCM) has made an important contribution to the prevention and control of the epidemic (Kang et al. 2022). The measures taken by TCM to treat and prevent COVID-19 include: (1) Fumigation with moxa in the room. (2) Wearing perfumed Chinese herb bag (including 8 kinds of herbs: clove, fineleaf schizonepeta herb, perilla frutescens, atractylodes lancea, cinnamon, biond magnolia flower, asarum sieboldii, and elettaria cardamomum, 2 g for each). They were crushed into powder and put into the bag, for external use. The herb powder was updated every 10 days. (3) Prescription of Chinese herbs for feet bath (including 3 kinds of herbs: vulgaris 10 g, carthamus 10 g, and dried ginger 6 g). The herbs were soaked into boiling water. When the temperature of the medicinal liquid is suitable, the feet were bathed into the medical liquid for about 20 min. (4) Prescription of Chinese herbs for prophylaxis (Including 8 Chinese herbal medicines: astragalus mongholicus 12 g, roasted rhizoma atractylodis macrocephalae 10 g, saposhnikovia divaricata 10 g, cyrtomium fortunei 10 g, honeysuckle 10 g, dried tangerine or orange peel 6 g, eupatorium 10 g, and licorice 10 g). Adults should decoct once a day for 5 days as a course of treatment, and the dosage for children should be halved. (5) Medical tea (Including 5 Chinese herbal medicines: perilla leaf 6 g, agastache leaf 6 g, dried tangerine or orange peel 9 g, stewed amomum tsao-ko 6 g, and 3 slices of ginger). The herbs were soaked into boiling water, and the medical liquid was consumed as tea. (6) Chinese patent medicines: *Huoxiang Zhengqi Capsule* or *Huoxiang Zhengqi Shui* (in half dose; Jin et al. 2020), *ShuFengJieDu Capsules* and *Lianhuaqingwen Capsule* may be the drug treatment options for COVID-19 (Lu 2020a). For the COVID-19 sequelae during the recovery period, *Jinshuibao tablets* and *Shengmaiyin oral liquid* significantly improved the cardiopulmonary function of recovering COVID-19 patients. *Shumian capsules* significantly improved patients’ sleep disorders. *Xiangsha Liujun pills* and *Ludangshen oral liquid* significantly improved digestive function (An et al. 2021). A multicenter observational study showed that TCM treatment for the post-COVID-19 patients may be a key to further promote rehabilitation and resolution of residual symptoms. Its therapeutic effects include the clinical symptom and lung function improvement, and recovery of a balanced body constitution (Zhong et al. 2022). A previous meta-analysis demonstrated that TCM could decrease the proportion of patients progressing to severe cases by 55% and the mortality rate of severe or critical patients by 49%. Moreover, TCM could relieve clinical symptoms, curtail the length of hospital stay, improve laboratory indicators, and so on. In addition, Kang et al. consulted the literature and obtained 149 components of Chinese medicinal herbs that could stably bind to antiviral targets or anti-inflammatory or immune-regulating targets by the prediction of molecular docking (Kang et al. 2022). Compared with Western medicine (WM), combined Chinese herbal medicine (CHM) and WM (CHM-WM) treatment for different severity of COVID-19 showed higher total effectiveness rate, lower symptom scores of fever, cough, fatigue, dry throat and pharyngalgia, shorter mean time to viral conversion, better computerized tomography image and blood results, fewer total adverse events and worse conditions. Subgroup analysis showed that the total effectiveness rate of combined CHM-WM group was significantly higher than WM group, especially for mild and moderate patients (Li et al. 2022). It suggested that the mechanisms involved anti-virus, anti-inflammation, and regulation of immunity (Kang et al. 2022).

### Medications

Medications may also have a role in the treatment and prevention of long COVID syndrome. A recent randomized placebo-controlled study including 1126 overweight and obese patients showed that metformin during the acute infection reduced the incidence of long COVID by 41.3% compared with placebo (Bramante et al. 2023). Other medications such as ivermectin and fluvoxamine were not shown to reduce the risk of long COVID and severe acute COVID-19 infection (Bramante et al. 2022, 2023).

### Potential novel therapies

There are many potential novel therapies such as the recombinant form of human ACE2 (rhACE2, APN01) which potentially both neutralize the virus and protect against acute lung injury. These interventions may enhance cardiovascular health by boosting natural immunity, promoting immune cell circulation, and reducing inflammation (Saha and Sharma 2022; Chen et al. 2022; Tsampasian et al. 2024; Adu-Amankwaah 2024). Recently, Aguida et al. found that near-infrared light exposure can stimulate mitochondrial metabolism to produce antioxidants, leading to an alleviation of proinflammatory signals caused by the SARS-CoV-2 infection (Aguida et al. 2023; Tangos et al. 2024). Previous studies have unveiled proprotein convertase subtilisin/kexin type 9 (PCSK9) inhibition involvement in various physiological processes, including cholesterol metabolism, inflammation, immune regulation, and thrombosis. The potential role of PCSK9 inhibition in the management of COVID-19 has emerged as an intriguing area of research. Preclinical studies suggest that PCSK9 inhibition could dampen inflammatory cascade by reducing the production of pro-inflammatory cytokines. Additionally, PCSK9 inhibition may protect against acute respiratory distress syndrome through its effects on lung injury and inflammation. PCSK9 inhibitors can lower LDL-C levels by enhancing the recycling of LDL receptors in the liver. Reduced LDL-C might protect blood vessels from further damage and lower the risk of atherosclerotic plaque formation. Moreover, PCSK9 inhibitors have potential antithrombotic effects in preclinical studies, which may mitigate the increased risk of coagulation disorders and thrombotic events in COVID-19 patients (Arsh et al. 2024). Besides the mentioned interventions, early medical attention, adherence to guidelines, supportive care, mental health support, individualized treatment plans, symptom monitoring, and staying informed are vital for preventing long COVID and its associated cardiovascular complications (Adu-Amankwaah 2024). In the cardiovascular system, targeting the persistent viruses and their enzymes, maintaining RAAS equilibrium, and enhancing immune responses emerge as logical approaches for addressing complications related to long COVID (Tsampasian et al. 2024; Adu-Amankwaah 2024).

## Cardiovascular side effects of COVID-19 treatment

It is worth noting that many drugs used to treat COVID-19 have serious cardiovascular side effects and toxicity as well as both conditions that need to be cautious or avoidance of these drugs. According to previous retrospective analysis, 37.8% of patients with COVID-19 revealed adverse drug events, of which 63.8% of all events were attributed to consumption of lopinavir/ritonavir (Sun et al. 2020). However, these data of side effects and toxicities come from patients that use these drugs chronically for the treatment of autoimmune diseases (chloroquine/hydroxychloroquine, rocilizumab), hepatitis (ribavarin, IFN-a), or HIV infection lopinivir/ritonivir). Therefore, the impact of short-term use of these drugs for COVID-19 patients is still unknown.

### Prolongation of QT interval

Recently, both anti-malarial and anti-autoimmune agents chloroquine and hydroxychloroquine (HCQ; Plaquenil) have been used to treat and prevent COVID-19. However, there were few data supporting the effectiveness of these drugs so far, and the cardiovascular side effects and toxicity are also considerable (Parvu et al. 2022; Chimenti et al. 2022). HCQ and chloroquine can inhibit the funny current channels (*I*_f_), delay rectifier potassium currents (*I*_Kr_) and L-type calcium ion currents (*I*_CaL_), and prolong the QT interval (Gautret et al. 2020; Giudicessi et al. 2020; Alblaihed et al. 2023). In COVID-19 patients with abnormal cardiac structure or function (such as left ventricular hypertrophy or reduced ejection fraction), QT prolongation may increase the risk of torsade de pointes ventricular tachycardia. Fortunately, the Heart Rhythm Society has recently released the management guidelines for QT prolongation in COVID-19 drug treatment and other cardiac electrophysiology issues related to COVID-19 (Lakkireddy et al. 2020). Nevertheless, these drugs should be avoided with concomitant QT-prolonging medications such as azithromycin and ritonavir/lopinavir; metabolic derangements; and renal failure (Yu et al. 2021). In a previous systematic review, Chatre et al. showed that the main side effects of these medicines were cardiac conduction disorder (85%). Moreover, these drugs had a long duration (median: 7 years) and a high cumulative dose. Other disadvantageous cardiovascular events included heart failure (26.8%), ventricular hypertrophy (22%), dyskinesia (9.4%), valve dysfunction (7.1%), and pulmonary hypertension (3.9%; Chatre et al. 2018). A large number of patients (44.9%) could return to normal cardiac function after discontinuing chloroquine and HCQ, while other patients continued to experience irreversible damage (12.9%) or die from drug side effects (30.8%; Liang et al. 2020). Therefore, these drugs should be carefully used, especially in the absence of more powerful data on their efficacy. The QT interval prolongation risk of favipiravir is considered to be low (Yu et al. 2021). No effect on the QT interval was observed in patients receiving ribavirin, tocilizumab, IFN-β treatment (Yu et al. 2021).

### Arrhythmia

Rare case reports showed that IFN-β induces premature ventricular captures and atrioventricular block (Yu et al. 2021). Fingolimod (FTY-720; an oral immunomodulating agent; antagonist of lipid sphingosine-1-phosphate 1 receptors in the lymph node T cells) has been reported to induce bradyarrhythmia, atrioventricular block (Yu et al. 2021). Dose-related asymptomatic prolongation in the PR interval with atazanavir treatment has been observed in clinical studies (Yu et al. 2021). Ritonavir/lopinavir has been shown to cause PR interval prolongation in some healthy adults, and second- or third-degree atrioventricular block in patients with underlying structural heart disease and pre-existing conduction system abnormalities (Yu et al. 2021). Tachycardia and PR interval prolongation have been documented in case reports of ivermectin treatment (Yu et al. 2021).

### Dyslipidemia

It is worth noting that tolizumab could affect lipid metabolism in patients with cardiovascular disease. It obviously increased TC, LDL-C and HDL-C levels, while significantly decreased high-density lipoprotein serum amyloid A (HDL-SAA), secretory phospholipase A2 IIA and lipoprotein (a) concentrations (Gabay et al. 2016). Some patients taking tocilizumab experienced rapid increases in LDL-C (Yu et al. 2021). Biological products i.e. tocilizumab and IFN-α 2B may occur hyperlipidemia or hypertriglyceridemia (Su et al. 2022).

### Impact on blood pressure

Fingolimod (FTY-720) has been reported to induce high blood pressure through increasing the vascular tone. It may also cause retinal arterial vasospasm and retinal vein occlusion (Yu et al. 2021). Although extensive cardiovascular toxicities and drug interactions have not yet been reported, prior evaluations of remdesivir during the Ebola outbreak noted that one patient developed hypotension and subsequent cardiac arrest (Lucey 2019; Yu et al. 2021). Some patients taking tocilizumab experienced rapid increases in blood pressure (Yu et al. 2021). Orthostatic hypotension has been documented in case reports of ivermectin treatment (Yu et al. 2021). Both tocilizumab and IFN-α 2B may occur hypertension (Su et al. 2022).

### Renal adverse effects

The renal adverse effects of monotherapy of lopinavir/ritonavir in COVID-19 patients, or combining antiviral agents metabolized by CYP3A4 with HCQ are significantly associated with a higher incidence of acute kidney injury such as a lower glomerular filtration rate (GFR) as well as glycosuria and proteinuria (Schneider et al. 2021; Jahanshahi et al. 2024). Remedsivir is also considered to have adverse effects on GFR (Jahanshahi et al. 2024).

### Others

Biological products (tocilizumab and IFN-α 2B), antiviral drugs (ribavarin, lopinivir or ritonivir), antimalarials (chloroquine or HCQ), and azithromycin used to treat COVID-19 may induce thrombocytopenia, anaemia, elevated liver transaminases (Su et al. 2022).

## Conclusions and future prospects

To sum up, the cardiovascular damage in COVID-19 patients is common and portends different prognosis across different populations. Therefore, differentiating between the various causes of cardiovascular damage in different populations is crucial to determining the treatment strategies. Worse clinical outcomes were found in COVID-19 patients with cardiovascular damage or pre-existing cardiovascular disease risk factors or cardiac disease; in Asian, Black and Latinx populations; in men, and in elderly peoples. These damages mainly manifested as myocarditis and pericarditis, hypertension, arrhythmia, myocardial injury and heart failure, CHD, stress cardiomyopathy, ischemic stroke, blood coagulation abnormalities, and dyslipidemia. The occurrence of these complications often aggravates COVID-19 and increases the mortality. Therefore, early recognition of these abnormalities among hospitalized COVID-19 patients is critical measures to identify patients with poor prognosis, guide treatment, and improve patients’ clinical outcomes. However, the long-term effects and their pathogenic mechanisms of SARS-CoV-2 on the cardiovascular system are not well-known (Tsampasian et al. 2024; Adu-Amankwaah 2024). The mechanisms sustaining the lingering effects of post-acute COVID-19 in the cardiovascular system may be associated with the variations in individuals’ genetic predispositions and changes in immune responses to SARS-CoV-2. The impact of RAAS on long COVID remains elusive (Tsampasian et al. 2024; Adu-Amankwaah 2024). This enigmatic phenomenon, was termed “long COVID” or “post-COVID-19 condition” by the World Health Organization (WHO; Tsampasian et al. 2024; Adu-Amankwaah 2024), and was defined as the “continuation or development of new symptoms 3 months after the initial SARS-CoV-2 infection, with the symptoms lasting for at least 2 months with no other explanation” (Soriano et al. 2022; Tsampasian et al. 2024; Adu-Amankwaah 2024). Nearly 45% of patients who have survived COVID-19 are contending with persistent symptoms even 4 months following the initial infection (O’Mahoney et al. 2022; Tsampasian et al. 2024; Adu-Amankwaah 2024). A 12-year follow-up study of 25 patients who recuperated from SARS-CoV infection revealed that they developed hyperlipidaemia (68%), cardiovascular disorders (44%), and glucose metabolism abnormalities (60%; Wu et al. 2017; Zheng et al. 2020; Adu-Amankwaah et al. 2021). Therefore, we still need to conduct long-term follow-up and a lot of research and exploration.

### Electronic supplementary material

Below is the link to the electronic supplementary material.

Supplementary Material 1

## Data Availability

Not applicable.
