# Supplementary material for: Pathogenic mechanisms of cardiovascular damage in COVID-19
Source: Mol Med. 2024 Jun 19;30:92. doi: 10.1186/s10020-024-00855-2 (PMC11186295; doi:10.1186/s10020-024-00855-2)
Supplement: Supplementary file 1 — Supplementary Material 1 [file 10020_2024_855_MOESM1_ESM.docx]

**Red = corrected or added;** **Blue = deleted**

**REVIEW**

**Cardiovascular damage and its pathogenetic mechanism of COVID-19 Pathogenic mechanisms of cardiovascular damage in COVID-19**

**Hong-Hua Shao^1^, and Rui-Xing Yin^1,2^*ID**

^1^ Department of Infectious Diseases, HIV/AIDS Clinical Treatment Center of Guangxi (Nanning), The Fourth People’s Hospital of Nanning, No. 1 Erli, Changgang Road, Nanning 530023, Guangxi, People’s Republic of China.

^2^ Department of Cardiology, Institute of Cardiovascular Diseases, The First Affiliated Hospital, Guangxi Medical University, 6 Shuangyong Road, Nanning 530021, Guangxi, People’s Republic of China.

*Correspondence:

Rui-Xing Yin

yinruixing@163.com

**ORCID:** Rui-Xing Yin: https://orcid.org/0000-0001-7883-4310

Cardiovascular damage of COVID-19

Hong-Hua Shao: 876380430@qq.com

Rui-Xing Yin: yinruixing@163.com

**Abstract**

COVID-19 is a new infectious disease caused by the severe acute respiratory syndrome coronavirus 2 (SARS CoV-2). Since the outbreak in 2019, it has caused an unprecedented world pandemic, leading to a global human health crisis. Although SARS CoV-2 mainly affects the lungs, causing interstitial pneumonia and severe acute respiratory distress syndrome, severe patients often have extensive clinical manifestations, such as gastrointestinal symptoms, cardiovascular damage and renal dysfunction. The most common cardiovascular manifestations are myocarditis, hypertension, arrhythmia, myocardial injury and heart failure, coronary heart disease, and venous and arterial thromboembolism events. Although the underlying pathophysiological mechanism of cardiovascular complications related to COVID-19 is not completely clear, both viral infection and hypersensitivity response of the body have been considered as the possible mechanisms of cardiovascular damage. This review article discusses the damage of SARSCoV-2 to the cardiovascular system and its pathogenetic mechanism, and providing some useful suggestions for future clinical prevention and treatment.

**Background** COVID-19 is a new infectious disease caused by the severe acute respiratory syndrome coronavirus 2 (SARS CoV-2). Since the outbreak in December 2019, it has caused an unprecedented world pandemic, leading to a global human health crisis. Although SARS CoV-2 mainly affects the lungs, causing interstitial pneumonia and severe acute respiratory distress syndrome, a number of patients often have extensive clinical manifestations, such as gastrointestinal symptoms, cardiovascular damage and renal dysfunction.

**Purpose** This review article discusses the pathogenic mechanisms of cardiovascular damage in COVID-19 patients and provides some useful suggestions for future clinical prevention and treatment.

**Methods** An English-language literature search was conducted in PubMed and Web of Science databases up to 12^th^ April, 2024 for the terms “COVID-19”, “SARS CoV-2”, “cardiovascular damage”, “myocardial injury”, “myocarditis”, “hypertension”, “arrhythmia”, “heart failure” and “coronary heart disease”, especially update articles in 2023 and 2024. Salient medical literatures regarding the cardiovascular damage of COVID-19 were selected, extracted and synthesized.

**Results** The most common cardiovascular damage was myocarditis and pericarditis, hypertension, arrhythmia, myocardial injury and heart failure, coronary heart disease, stress cardiomyopathy, ischemic stroke, blood coagulation abnormalities, and dyslipidemia. Two important pathogenic mechanisms of the cardiovascular damage may be direct viral cytotoxicity as well as indirect hyperimmune responses of the body to viral infection.

**Conclusions** Cardiovascular damage in COVID-19 patients is common and portends a worse prognosis. Although the underlying pathophysiological mechanisms of cardiovascular damage related to COVID-19 are not completely clear, two important pathogenic mechanisms of cardiovascular damage may be the direct damage of the SARSCoV-2 infection and the indirect hyperimmune responses.

**Keywords** COVID-19,; SARS-CoV-2,; Cardiovascular damage,; Myocardial injury, Myocarditis,; Hypertension,; Myocardial injury, Arrhythmia

**1 | INTRODUCTION Introduction**

Novel coronavirus pneumonia (NCP) has led to a world pandemic, which poses a huge threat to the safety of human life. It is a novel identified human acute infectious disease caused by severe acute respiratory syndrome coronavirus 2 (SARS-CoV-2). In December 2019, the first case of pneumonia caused by novel coronavirus appeared in Wuhan, China (Li et al. 2020; Wang et al. 2020a).^1,2^ On January 12, 2020, the World Health Organization (WHO) temporarily named the novel coronavirus that caused the Wuhan pneumonia epidemic as 2019 novel coronavirus (2019 nCoV). On February 12, 2020, the WHO officially named that novel coronavirus was SARS CoV-2, and the disease caused by SARS CoV-2 was coronavirus disease 2019 (COVID-19). On March 11, 2020, WHO announced that this epidemic of coronavirus pneumonia was another world pandemic since the 2009 influenza A (H1N1) pandemic. So far, the number of persons infected worldwide has exceeded 635 million, causing more than 6.59 million deaths (Firouzabadi et al. 2023),^3^ and the number of infections and deaths is still rising. SARS CoV-2 mainly infects the respiratory tract and lungs. The main clinical manifestation is symptoms of influenza syndrome. Mild cases manifest as asymptomatic subclinical infections. In severe patients, it may occur acute respiratory distress syndrome (ARDS) which requires mechanical ventilation and concurs multiple organ failure. Although COVID-19 has been initially associated with respiratory system, it has become rapidly clear that it may affect multiple important organs including the heart (Tomasoni et al. 2020).^4^ Severe patients can also be accompanied by gastrointestinal symptoms, and may even experience cardiac and renal dysfunction. Cardiac damage in COVID-19 patients is associated with poor prognosis and may lead to an increase in mortality rates. Although the causal relationship between COVID-19 and cardiac damage is not yet clear, current evidence suggests that myocardial inflammation is accompanied or not accompanied by direct myocardial damage, suggesting different pathophysiological mechanisms of COVID mediated myocardial damage. According to previous reports, 19-28% of COVID-19 patients experience cardiac damage (Shi et al. 2020; Guo et al. 2020b; Bonow et al. 2020).^5-7^ For example, 11.8% of the deceased showed elevated troponin I or sudden cardiac arrest during hospitalization (Zheng et al. 2020);^8^ 33% of COVID-19 deaths can also be attributed to heart disease (Ruan et al. 2020).^9^ The occurrence of cardiovascular complications may also affect the severity of COVID-19, and potential cardiovascular diseases may increase mortality (Santoso et al. 2021; Tian et al. 2020).^10,11^ Although the clinical manifestations and laboratory test results of COVID-19 patients have been reported widely, data are scant in the cardiovascular damage and its pathogenetic mechanism. Therefore, an in-depth understanding the cardiovascular damage caused by SARS-CoV-2 and the underlying pathogenetic mechanisms may improve the diagnosis, treatment and prognosis of COVID-19 patients. This article reviews the cardiovascular damage caused by SARS-CoV-2 infection and its pathogenetic mechanism. It may provide some useful reference for future prevention, therapy and control.

COVID-19 is a novel identified acute infectious disease caused by severe acute respiratory syndrome coronavirus 2 (SARS-CoV-2). Since the outbreak in December 2019, it has caused an unprecedented world pandemic, leading to a global human health crisis (Li et al. 2020; Wang et al. 2020a). As of December 24, 2023, a total of 773,119,173 persons have been infected with COVID-19 worldwide. Of them, 6,990,067 people died, with an overall mortality rate of 1% (Choi et al. 2024; Hatch et al. 2024) to 4% (Shu et al. 2023). At present, the prevalence of COVID-19 has slightly improved with virus mutation and population vaccination, but the number of infections and deaths is still rising. Although COVID-19 has been initially associated with respiratory system, it has become rapidly clear that it may affect multiple important organs including the heart (Tomasoni et al. 2020; Del Vecchio et al. 2024). COVID-19 may directly exacerbate pre-existing heart disease and frequently induce new cardiovascular complications (Burger et al. 2021). There is a two-way relationship between COVID-19 and cardiovascular disease. Pre-existing cardiovascular risk factors, such as hypertension, diabetes, and chronic cardiovascular diseases, are easy to cause serious COVID-19. On the contrary, COVID-19 can lead to cardiovascular complications (Ozcan et al. 2023). The mechanisms involving these cardiovascular complications of COVID-19 may include direct myocardial injury, systemic inflammation and cytokine storm, downregulation of ACE2 receptors, mismatch of myocardial oxygen demand-supply, plaque rupture and coronary thrombosis, electrolyte imbalances, diffused endothelial damage, coagulation abnormalities characterized by hypercoagulation and microthrombosis, and adverse effects of therapies (Parvu et al. 2022; Zhao et al. 2023; Ozcan et al. 2023; Tangos et al. 2024). According to previous reports, 19-28% of COVID-19 patients experience cardiac damage (Shi et al. 2020; Guo et al. 2020b; Bonow et al. 2020), 33% of COVID-19 deaths can also be attributed to heart disease (Zheng et al. 2020; Ruan et al. 2020). The occurrence of cardiovascular complications may also affect the severity and increase mortality of COVID-19 (Santoso et al. 2021; Tian et al. 2020). Although the clinical manifestations and laboratory test results of COVID-19 patients have been reported widely, the data regarding the pathogenetic mechanism of cardiovascular damage in COVID-19 patients remain scant. Therefore, this article addresses the cardiovascular damage caused by SARS-CoV-2 infection and its pathogenetic mechanism. It may provide some valuable reference materials for clinicans to future prevent, diagnose and treat.

**Search methodology**

A literature search was performed through systematical search for current finding mainly from PubMed and Web of Science databases from 12^th^ December, 2019 to 12^th^ April, 2024. The literature search was restricted to studies published or translated into English. The search strategy of medical subject headings and keywords combined with entry terms was utilized to search all related literatures. The keywords were selected based on the previous publications, and all the terms used were based on the criterion that they were present in the titles, abstracts, and keywords of the articles. The following defined words were used as search strategy to obtain relevant information: “COVID-19”, “SARS CoV-2”, “cardiovascular damage”, “myocardial injury”, “myocarditis”, “hypertension”, “arrhythmia”, “heart failure” and “coronary heart disease”, especially update articles in 2023 and 2024. The inclusion of references was based on documents displayed information on cardiovascular damage and myocardial injury associated with COVID-19. Salient medical literatures regarding the cardiovascular damage of COVID-19 were selected, extracted and synthesized.

**2 | STRUCTURE AND BIOLOGICAL PROPERTIES OF SARS-CoV-2 Structure and biological properties of SARS-CoV-2**

Coronavirus (CoV) is a large class of viruses widely present in nature. It is hosted by birds, mammals and humans, and can cause respiratory tract, gastrointestinal tract, liver and nervous system diseases. Under the electron microscope, this kind of virus is spherical or oval particles, and there are regularly arranged cystic collagen fiber processes, which are similar to the royal crown, so it is named as coronavirus. Coronavirus has a single-stranded RNA genome with a positive sense and cyst membrane. Its diameter is 80~120 nm. It belongs to the Riboviridia, Nidovirales, Coronaviridae, and Orthocoronavirinae, and can be divided into α, β, γ and δ four genera (Genus) (Guzik et al. 2020).^12^ Coronavirus can infect many animal species, such as bats, dogs, pigs, mice, birds, cattle, whales, horses, goats, monkeys, etc. It is also susceptible to humans and can cause infectious diseases of multiple important organs. There are 6 previously known human coronaviruses, namely α Genus: human coronavirus 229E (HCoV-229E), human coronavirus NL63 (HCoV-NL63); β Genus: human coronavirus OC43 (HCoV OC43), human coronavirus Hong Kong University 1 (HCoV HKU1), SARS coronavirus (SARS CoV), Middle East respiratory syndrome related coronavirus (MERS CoV). Among them, 4 kinds of coronaviruses: 229E, NL63, OC43 and HKU1 are common in the population, but their pathogenicity is low, and they can cause mild respiratory symptoms similar to common cold in people with normal immunity. The other two kinds of coronaviruses: SARS CoV and MERS CoV are animal derived coronaviruses, which can cause severe acute respiratory syndrome (SARS) and Middle East respiratory syndrome (MERS) respectively, with mortality rates of 10% and 37% respectively (Huang et al. 2020).^13^

Due to its wide distribution, genetic diversity, frequent recombination, and the expansion of human activities, the coronavirus often spreads across species. In this way, novel coronavirus is likely to infect humans regularly. The newly discovered SARS CoV-2 is the seventh coronavirus that can infect humans and belongs to β Genus Mesosak β Coronavirus (Sarbecovirus) subgenus. There is an envelope, the particle is round or oval, often polymorphic, with a diameter of 50-200 nm, and it is a single-stranded positive-sense RNA virus with genomes ranging between 26.2 and 31.7 kb RNA. SARS CoV-2 has five essential genomes, namely virus envelope, nuclear protein, matrix protein, spike protein, RNA dependent RNA polymerase (RdRp, **Figure 1**). The spike (S), nucleocapsid (N), membrane (M), and envelope (E) proteins were four major structural proteins encoded by the genome, all required to produce the viral particle (Schoeman and Fielding 2019).^14^ Viral nucleoprotein will wrap around the RNA gene cluster to form a nucleocapsid, known as the nucleocapsid (N) protein, which wraps the viral genome. There are spike (S) protein and envelope (E) protein on the capsule. Spike (S) protein plays an important role in the process of virus infection of target cells. SARS CoV-2 can enter the host cells by the binding of spike (S) protein and the corresponding receptors such as angiotensin converting enzyme 2 (ACE2) and CD26 receptors (Hoffmann et al. 2020; Lim et al. 2020).^15,16^ ACE2 is a membrane-bound peptidase that expression is very high on cell surface of many tissues and organs including the respiratory, circulatory, urinary, and digestive systems, and it is involved in the regulation of several cardiovascular and immune pathways (Zheng et al. 2020; Turner et al. 2004; Hooper et al. 2020).^8,17,18^ Moreover, spike protein has a strong binding affinity with ACE2 receptor, and its binding free energy is –50.6 kcal/mol (Xu et al. 2020a).^19^ In human type II alveolar epithelial cells (AT2 cells), macrophages and other cell types, esophageal epithelial cells and layered epithelial cells, absorptive intestinal epithelial cells of ileum and colon, the expression of ACE2 receptor is very high, suggesting that gastrointestinal tract may also be a potential pathway of SARS CoV-2 infection (Zhang et al. 2020).^20^ For example, the first patient in the United States was diagnosed after virus nucleic acid was detected in stool. Spike (S) protein can be divided into S1 and S2 subunits (**Figure 2**). The S1 subunit has the N-terminal (S1-NTD) and C-terminal domains (S1-CTD). These regions are the sites binding to various cellular receptors, and the binding domains of these receptors contain carbohydrates or proteins. For example, the receptor binding domain (RBD) of S1 can bind to the ACE2 receptor on the cell surface. This process requires viral S protein activation through the cellular serine protease transmembrane serine protease 2 (TMPRSS2). The S2 subunit of the trimer has two heptapeptide repeat (HR) sequences of HR1 and HR2, which mediate the fusion of the virus with the infected cell membrane. The S protein of SARS CoV and SARS CoV-2 is similar in gene sequence (76.47%) and structure (Wan et al. 2020),^21^ indicating that they may share the same or common receptors. In addition to ACE2, the protease furin is also needed to promote entrance of the virus into the cell (Walls et al. 2020).^22^ There is also a conserved Furin recognition site on the S1/S2 cleavage site of SARS-CoV-2, which is directly related to the high pathogenicity of the virus. In addition, the second protease cleavage site called S2' is located near the N-terminus of S2. The genome size of SARS-CoV-2 is 29,891 nucleotides, including the UTR gene at both ends and a complete open reading frame (ORF) gene, which can encode 9,860 amino acids (Ren et al. 2020).^23^ The genome of SARS CoV-2 has a cap structure at the 5' end and a polyadenylate poly (A) tail at the 3' end. From the 5' end to the 3' end, there are 12 ORFs respectively: ORF1a, ORF1b, S, ORF3a, E, M, ORF6, ORF7a, ORF7b, ORF8, N and ORF10 (**Figure 2**). Among them, there are two large reading frames ORF1a and ORF1b in the first 2/3 of the genome, which can encode two replicase related multi protein precursors pp1a and pp1ab. Coronavirus needs to transpose a nucleotide (–1) from the end of ORF1a to the 5' end to synthesize 1b through the mechanism of ribosome reading frame displacement, and finally translate into the complete multi protein precursor pp1ab. Studies have shown that it can form a double membrane vesicular organelle like structure with the size of 200~350 nm in the cytoplasm after infecting cells, which is an important place for viral transcription and replication. The sequence of SARS-CoV-2 genome is 5'-replicase polysaccharide protein (orf1/ab)-structural protein [spike (S) glycoprotein-small envelope (E) glycoprotein-membrane (M) glycoprotein -nucleocapsid (N) protein-3' (Chan et al. 2020a).^24^ The length of its RNA sequence is about 300,000 nucleotides (Liu et al. 2020a).^25^ The SARS CoV-2 genome is highly variable at two core locations (silent variation in ORF1ab gene and polymorphism in ORF8 amino acid). The mutation in ORF8 will lead to its two variants (ORF8-L and ORF8-S), leading to structural abnormalities of the protein (Ceraolo and Giorgi 2020).^26^ For example, a single N501T mutation may significantly strengthen the binding affinity of SARS CoV-2 RBD and human ACE2. In addition, spike glycoprotein and nucleocapsid protein are also prone to mutation (Benvenuto et al. 2020).^27^ The mutation of the virus may be due to the genetic information change in the process of its rapid genetic material replication, or it may be due to the genetic material recombination between live viruses. This suggests that SARS CoV-2 has unique genomic and structural characteristics, and further research is needed to clarify its role in viral replication and pathogenesis.

The newly discovered SARS CoV-2 is the seventh coronavirus. It belongs to β Genus Mesosak β Coronavirus subgenus (Guzik et al. 2020). The viral particle is often polymorphic, with a diameter of 50-200 nm, and it is a single-stranded positive-sense RNA virus with genomes ranging between 26.2 and 31.7 kb RNA. SARS CoV-2 has four major structural proteins: spike (S), nucleocapsid (N), membrane (M), and envelope (E) proteins, all required to produce the viral particle (Huang et al. 2020; Schoeman and Fielding 2019; Chan et al. 2020a; **Figure 1**). SARS CoV-2 can bind to the surface-bound peptidase angiotensin-converting enzyme 2 (ACE2, EC 3.4.17.23) or CD26 receptors leading to tissue infection and viral replication (Hoffmann et al. 2020; Lim et al. 2020; Chappell 2023). When SARS-CoV-2 binds to ACE2, internalization of the SARS-CoV-2-ACE2 complex reduced ACE2 activity, and subsequent activation of the renin-angiotensin system (RAS) [higher Ang II/Ang-(1-7) ratio] that may exacerbate the acute inflammatory events in COVID-19 patients and possibly contribute to the effects of long COVID-19 (Chappell 2023). In addition, it can activate metalloproteinase 17 (ADAM17), which induces ACE2 membrane shedding, exacerbates the accumulation of Ang II, and diminishes the cardioprotective effects of ACE2 (Gheblawi et al. 2020; Zhao et al. 2023). Moreover, COVID-19 patients present with an array of autoantibodies to various components of the RAS including the peptide Ang II, the enzyme ACE2, and the AT1, AT2 and Mas receptors (Chappell 2023). The density of ACE2 receptors is very high on cell surface of many tissues and organs including human type II alveolar epithelial cells, macrophages and other cell types, esophageal epithelial cells and layered epithelial cells, absorptive intestinal epithelial cells of ileum and colon, and it is involved in the regulation of several cardiovascular and immune pathways (Zheng et al. 2020; Hooper et al. 2020). Increased expression of plasma soluble ACE2 was found in patients with cardiovascular diseases such as myocardial infarction, atrial fibrillation, valvular disease, and heart failure, reflecting a higher basal ACE2 expression and increased susceptibility in this condition (García-Escobar et al. 2022; Silva et al. 2022). Currently, gene expression studies showed that human ventricular myocardium contains all the requisite mediators of SARS-CoV-2 binding and entry. In the heart, ACE2 is expressed more in cardiomyocytes and pericytes than that in endothelial cells and fibroblasts (Chung et al. 2021; Shu et al. 2023). In addition, pericytes, which support the microvasculature throughout the myocardium, appear particularly susceptible with robust expression of ACE2 (Chung et al. 2021). Moreover, S protein has a strong binding affinity with ACE2 receptor, and its binding free energy is –50.6 kcal/mol (Xu et al. 2020a). Gastrointestinal tract may also be a potential pathway of SARS CoV-2 infection (Zhang et al. 2020). S protein includes S1 and S2 subunits (**Figure 2**). The S1 subunit has the N-terminal domain (S1-NTD) and C-terminal domain (S1-CTD). These regions are the sites binding to various cellular receptors, and the binding domains of these receptors contain carbohydrates or proteins. For example, the receptor binding domain (RBD) of S1 can bind to the ACE2 receptor on the cell surface. This process requires viral S protein activation through the cellular serine protease transmembrane serine protease 2 (TMPRSS2). The S2 subunit of the trimer has two heptapeptide repeat (HR) sequences of HR1 and HR2, which mediate the fusion of the virus with the infected cell membrane. In addition, the protease Furin is also needed to promote entrance of the virus into the cell (Walls et al. 2020). There is also a conserved Furin recognition site on the S1/S2 cleavage site of SARS-CoV-2, which is directly related to the high pathogenicity of the virus. The second protease cleavage site called S2' is located near the N-terminus of S2. The genome size of SARS-CoV-2 is 29,891 nucleotides, including the UTR gene at both ends and a complete open reading frame (ORF) gene, which can encode 9,860 amino acids (Ren et al. 2020; Liu et al. 2020a). The genome of SARS CoV-2 has a cap structure at the 5' end and a polyadenylate poly (A) tail at the 3' end. From the 5' end to the 3' end, there are 12 ORFs respectively: ORF1a, ORF1b, S, ORF3a, E, M, ORF6, ORF7a, ORF7b, ORF8, N and ORF10 (Walls et al. 2020; Chan et al. 2020a). Among them, there are two large reading frames ORF1a and ORF1b in the first 2/3 of the genome, which can encode two replicase related multi protein precursors pp1a and pp1ab. SARS-CoV-2 needs to transpose a nucleotide (–1) from the end of ORF1a to the 5' end to synthesize 1b through the mechanism of ribosome reading frame displacement, and finally translate into the complete multi protein precursor pp1ab. The genome of SARS CoV-2 is highly variable at two core locations (silent variation in ORF1ab gene and polymorphism in ORF8 amino acid). The mutation in ORF8 will lead to its two variants (ORF8-L and ORF8-S), leading to structural abnormalities of the protein (Ceraolo and Giorgi 2020). For example, a single N501T mutation may significantly strengthen the binding affinity of SARS CoV-2 RBD and human ACE2. In addition, S and N proteins are also prone to mutation (Benvenuto et al. 2020).

Based on gene expression studies, human ventricular myocardium contains all the requisite mediators of SARS-CoV-2 binding and entry. Although TMPRSS2 is minimally expressed in the heart, there is high expression of ACE2, and other proteases (eg, FURIN, NRP1, CTSB/L) known to participate in priming and membrane fusion, and integrin co-receptors appear to be ubiquitously expressed

**3 | EPIDEMIC CHARACTERISTICS OF COVID-19 Epidemic characteristics of COVID-19**

The sequence of SARS-CoV-2 genome shares 75% to 80% homology with SARS CoV (Lake 2020),^28^ which was closer to several bat coronaviruses (Andersen et al. 2020)^29^ and more than 85% to bat SL CoVZC45. Therefore, it is speculated that its natural host may be bats (such as the Chinese chrysanthemum bat), and bats can host many kinds of animals β-CoV (Lu et al. 2020b).^30^ The transmission route may be bat human or bat intermediate host human. Pigs, ferrets, cats and primates may be their intermediate hosts (Wan et al. 2020).^21^ After the cross species transmission of wild animals to humans, there is also the transmission between people. Therefore, infected patients and asymptomatic infected people are the main source of infection of COVID-19. The incubation period has infectivity, and the infectiousness is very high within 5 days after onset. The important epidemic feature is aggregation, such as community or family aggregation transmission. A previous report showed that 49.5% of patients were infected through family gatherings and social activities, where 5 couples infected each other, 6 patients infected after attending a ceremony in a local temple, 5 patients infected after a dinner party, and some patients infected during square dancing (Lian et al. 2020).^31^ Asymptomatic infection mainly refers to recessive infection and patients at the end of incubation period. The incubation period is generally 1~14 days, mostly 3~7 days, and very few cases can reach 24 days (Chan et al. 2020b; Chen et al. 2020c; Jin et al. 2020).^32-34^ The incubation period is infectious, and asymptomatic infected persons may also become the source of infection (Andersen et al. 2020; Rothe et al. 2020).^29,35^ The basic infection number (RO) value of SARS CoV-2 at the initial outbreak stage is greater than 1:2.2-2.9 (Li et al. 2020; Callaway et al. 2020; Zhao et al. 2020),^1,36,37^ that is, each infected person can infect the virus to at least three normal people, indicating that it is highly infectious (Jiang et al. 2020).^38^ The transmission rate of SARS CoV-2 was higher than that of SARS CoV and influenza. The viral load of infected persons was more than 1 billion RNA copies per ml of sputum. Severe COVID-19 patients often had higher viral load and longer viral transmission period than the mild patients. Respiratory droplets and close contact are the two main routes of transmission. Contact with viral contaminated items such as saliva, nasal mucus, feces and urine can also cause infection. It cannot be ruled out that it can be transmitted through fecal mouth, nor can it be ruled out that contaminated food can be transmitted through mouth. When exposed to high concentrations of aerosols for a long time in a relatively closed environment, it is possible to propagate through aerosols. Therefore, attention should be paid to contact transmission or aerosol transmission caused by environmental pollution. There are also reports of the possibility of mother to child vertical transmission. The distance of droplet transmission is generally not more than 1 m because the droplet diameter is usually greater than 5 μm. Within 1 m, the droplet will settle on the surface of the object and become the source of contact propagation. When the droplet is small, the water in the droplet can evaporate to form aerosols. Contact transmission is caused by droplets containing SARS CoV-2 or other excreta of patients contaminating the surface of objects. The hands are usually the last link of infection after human contact. After hands are contaminated, they are infected by touching the face, nasal mucosa and oral mucosa. Aerosol transmission refers to small droplets floating in the air, which can be transmitted over a long distance through inhalation of respiratory tract infection. It has been reported that the nucleic acid of SARS CoV-2 can be detected in the feces of patients, and the virus can be isolated (Holshue et al. 2020),^39^ indicating that there is a virus in the feces, but its significance in transmission is still unclear. If the environment is polluted, it can be transmitted by contact, and if aerosol is formed, it can also be transmitted. The population is generally lack of immunity and susceptible. The symptoms of pediatric cases are relatively mild, and some children and newborns may experience atypical symptoms (Shen and Yang 2020).^40^ The aged, people with chronic basic diseases (hypertension, diabetes, cardiovascular diseases, etc.), late pregnancy and perinatal women, and obese individuals are seriously ill after infection (Zheng et al. 2020; Chan et al. 2020b; Wu et al. 2020; Zhou et al. 2020a; Tang et al. 2020).^8,32,41-43^ After infection or inoculation with novel coronavirus vaccine, certain immunity can be obtained, but the duration is not clear.

The transmission route of COVID-19 may be bat human or bat intermediate host human. Pigs, ferrets, cats and primates may be their intermediate hosts (Wan et al. 2020; Lake 2020; Andersen et al. 2020; Lu et al. 2020b). After the cross species transmission of wild animals to humans, there is also the transmission between people. Therefore, infected patients and asymptomatic infected people are the main source of infection of COVID-19 (Andersen et al. 2020; Rothe et al. 2020). Cholesterol in the host cell plasma membrane plays an important role in the SARS-CoV-2 entry into cells (Chidambaram et al. 2022b). The transmission of SARS CoV-2 increases proportionally with rising levels of cholesterol in the cell membrane. This is due to the fact that cholesterol increases the number of viral entry spots and the concentration of ACE2 receptor, crucial for viral penetration (Kowalska et al. 2022). The infectiousness is very high within 5 days after onset. The important epidemic feature is aggregation, such as community or family aggregation transmission (Lian et al. 2020). The incubation period is generally 1~14 days, mostly 3~7 days, and very few cases can reach 24 days (Chan et al. 2020b; Chen et al. 2020c; Jin et al. 2020). The basic infection number (RO) value of SARS CoV-2 at the initial outbreak stage is greater than 1:2.2-2.9 (Li et al. 2020; Callaway et al. 2020; Zhao et al. 2020), indicating that it is highly infectious (Jiang et al. 2020). The viral load of infected persons was more than 1 billion RNA copies per ml of sputum. Severe COVID-19 patients often had higher viral load and longer viral transmission period than the mild patients. Respiratory droplets and close contact are the two main routes of transmission. Contact with viral contaminated items such as saliva, nasal mucus, feces and urine can also cause infection (Holshue et al. 2020). It cannot be ruled out that it can be transmitted through fecal mouth, nor can it be ruled out that contaminated food can be transmitted through mouth. When exposed to high concentrations of aerosols for a long time in a relatively closed environment, it is possible to propagate through aerosols. Therefore, attention should be paid to contact transmission or aerosol transmission caused by environmental pollution. There are also reports of the possibility of mother to child vertical transmission. Contact transmission is caused by droplets containing SARS CoV-2 or other excreta of patients contaminating the surface of objects. The hands are usually the last link of infection after human contact. After hands are contaminated, they are infected by touching the face, nasal mucosa and oral mucosa. Aerosol transmission refers to small droplets floating in the air, which can be transmitted over a long distance through inhalation of respiratory tract infection. The symptoms of pediatric cases are relatively mild, and some children and newborns may experience atypical symptoms (Shen and Yang 2020; Vasichkina et al. 2023). The aged, people with chronic basic diseases (hypertension, diabetes, cardiovascular diseases, etc.), late pregnancy and perinatal women, and obese individuals are seriously ill after infection (Zheng et al. 2020; Chan et al. 2020b; Wu et al. 2020; Zhou et al. 2020a; Tang et al. 2020).

**4 | CARDIOVASCULAR DAMAGE AND ITS PATHOGENETIC MECHANISM OF COVID-19 Pathogenic mechanisms of cardiovascular damage in COVID-19**

COVID-19 not only impacts the respiratory system but often exhibits extrapulmonary involvement, resulting in systemic disease (Ozcan et al. 2023). Therefore, the clinical presentations of COVID-19 are multifaceted and vary widely, mainly include the general features of respiratory infection and the special manifestations of extrapulmonary complications such as cardiovascular, cerebrovascular, gastrointestinal, musculoskeletal, endocrine, and renal systems. Many patients with COVID-19 can develop different types of cardiovascular complications during hospitalization (Pellicori et al. 2021), which may be a part of post-acute infection sequelae (Tsampasian et al. 2024). Approximately 62% of the patients hospitalized with COVID-19 have been reported to have acute myocardial injuries (Shu et al. 2023). Myocardial involvement may be a feature of long COVID syndrome from the early months of the COVID-19 pandemic (Tsampasian et al. 2024). About 45% of COVID-19 survivors experienced persistent symptoms at 4 months post the acute infection (O'Mahoney et al. 2022; Tsampasian et al. 2024; Adu-Amankwaah 2024). Systematic review confirms that chest pain, palpitations, dyspnoea, and syncope are the most commonly reported symptoms among patients with long COVID syndrome (Tsampasian et al. 2024). The patients with underlying cardiac disease or the presence of cardiovascular disease risk factors are more likely to experience serious outcomes (Tian et al. 2020). The clinical manifestations of cardiovascular damage in COVID-19 patients include myocarditis and pericarditis, hypertension, arrhythmia, myocardial injury and heart failure, coronary heart disease, stress cardiomyopathy, ischemic stroke, blood coagulation abnormalities, and dyslipidemia (Adu-Amankwaah et al. 2021; Zhao et al. 2023; Tsampasian et al. 2024; **Figure 3**). Long COVID-19 can affect almost all organs of the body, and can lead to more than 200 different clinical manifestations (Gyöngyösi et al. 2023). There are strong evidences demonstrating that pre-existing obesity, heart failure, and ischemic heart disease are significant risk factors for the development of long COVID syndrome. However, there is conflicting data in literature about other cardiovascular diseases such as hypertension, cholesterol, atrial fibrillation, and diabetes mellitus (Tsampasian et al. 2024).

There may be racial and ethnic disparities in COVID-19 outcomes. Patients from ethnic minority groups are disproportionately affected by COVID-19 (Magesh et al. 2021). A previous meta-analysis showed that the risk of COVID-19 infection was higher in the individuals of Black and Asian ethnicities compared to White individuals. Moreover, Asians may have higher risk of intensive therapy unit admission and death (Sze et al. 2020). Higher mortality rates of COVID-19 were also found in Black and Latinx populations (Gross et al. 2020; Yancy 2020), and in predominantly Black compared with White populated counties (Yancy 2020; Scannell et al. 2020). In an analysis of 7868 patients hospitalized with COVID-19, Hispanics, non-Hispanic Black, Asian, and non-Hispanic White were 33%, 25.5%, 6.3%, and 35.2%; respectively. However, Asian patients had the highest cardiorespiratory severity score (Rodriguez et al. 2021). In the UK, COVID-19-related disease severity and mortality were higher in Black, Asian, mixed and other ethnic groups than in the White ethnic group majority (Siddiq et al. 2023). A meta-analysis comprising 4,318,929 patients from 68 studies also showed that White race, Hispanic/Latino, African American, multiracial and another race or ethnicity, Asian American, American Indian or Alaska Native, Pacific Islander account for 24.0%, 19.7%, 8.6%, 6.2%, 2.4%, 0.2%, and 0.2%; respectively. African American and Hispanic individuals were the most likely to test positive for COVID-19. Asian American individuals had the highest risk of intensive care unit admission (Magesh et al. 2021). Compared with White majority populations, Irizar et al. observed an increased risk of testing positive for infection for people from Black, South Asian, Mixed, and Other ethnic groups. Black, Hispanic, and South Asian people were more likely to be seropositive. Among population-based studies, Black and Hispanic ethnic groups and Indigenous peoples had an increased risk of hospitalization. Black, Hispanic, South Asian, East Asian and Mixed ethnic groups and Indigenous peoples had an increased risk of ICU admission. Mortality risk was increased for Hispanic, Mixed, and Indigenous groups. Smaller differences were seen for prognosis following infection. Following hospitalisation, South Asian, East Asian, Black and Mixed ethnic groups had an increased risk of ICU admission, and mortality risk was greater in Mixed ethnic groups (Irizar et al. 2023).

The progression and outcome of COVID-19 may have sex differences (Widmann et al. 2024; Tangos et al. 2024). Several studies have revealed that the morbidity, severity, and mortality of COVID-19 were higher in men than in women (Chen et al. 2020d; Haitao et al. 2020; Alwani et al. 2021; Wehbe et al. 2021; Marik et al. 2021; Torres et al. 2023; Woodruff et al. 2023; Chappell 2023). The prevalence of COVID-19 in combination with cardiovascular disease was also higher in male than in female (Gebhard et al. 2020). The mortality in patients with acute myocardial infarction and COVID-19 (Yeo et al. 2023) or heart failure and COVID-19 (Isath et al. 2023) was higher in men than in women. In a systematic review and meta-analysis, there were also different in the sex, racial, and ethnic representation among COVID-19 prevention and treatment trials (Xiao et al. 2023). These findings suggest that men may have higher susceptibility to and more severe adverse clinical outcomes from SARS-CoV-2 infection than women. The reasons for sex differences in COVID-19 may be attributed to the difference in the expression levels of ACE2 and type II transmembrane serine protease (TMPRSS2), sex hormones, and immune and inflammatory responses (Viveiros et al. 2021; Thomas et al. 2021; Zhao et al. 2023). Plasma ACE2 concentrations and TMPRSS2 expression were higher in males than in females, TMPRSS2 is regulated by androgens (Okwan-Duodu et al. 2021), which may lead to an increased initial viral load (Viveiros et al. 2022). ACE2 and TMPRSS2 are key factors in promoting SARS-CoV-2 entry into cells (Hoffmann et al. 2020). Females can express higher amounts of toll-like receptor 7 (TLR7), which recognizes single-stranded RNA and promotes interferon production, playing an important role in the immune response to SARS-CoV-2 infection (Bienvenu et al. 2020; Wehbe et al. 2021; Zhao et al. 2023). Estradiol enhances the antiviral response by increasing the number of neutrophils and natural killer cells and decreasing pro-inflammatory cytokines, whereas androgens have immunosuppressive effects (Viveiros et al. 2021; Bechmann et al. 2022; Brandi 2022). Androgen receptors are transcription promoters for Transmembrane protease serine 2 (TMPRSS2) and can, therefore, facilitate SARS-COV2 entry. Genetic variants in the androgen receptor were correlated with androgen sensitivity (Mohamed et al. 2021). However, Bugiardini et al. found that women in general wards were at increased risk of acute heart failure and in-hospital mortality for COVID-19 compared with men. For patients receiving intensive care unit care, fatal complications including acute heart failure and mortality appeared to be independent of sex (Bugiardini et al. 2023).

Age is an uncontrollable risk factor for increased mortality in COVID-19 patients. The susceptibility is higher as well as the clinical outcomes is more severe in older than in younger people (Bonanad et al. 2020; Zhou et al. 2020a; Torres et al. 2023; Tangos et al. 2024). Several previous studies also demonstrated that the risk of cardiovascular complications was greatly increased in the elderly COVID-19 patients. The risk of cardiovascular disease in COVID-19 patients was increased with age (Pellicori et al. 2021). The expression of ACE2 in SARS-CoV-2 infected cells was reduced, especially in elderly people. Thus, elderly people with down-regulated ACE2 expression have more severe adverse outcomes when infected with SARS-CoV-2 (AlGhatrif et al. 2020). These findings suggest that individualized differences, different populations tolerate and respond differently to the COVID-19 (Liu et al. 2021b; Tobler et al. 2022). However, Giugni et al. found that younger age is associated with cardiovascular pathological phenotype of severe COVID-19 at autopsy. They showed that younger age is associated with cardiovascular abnormalities such as acute heart ischemia, myocarditis and lung angiomatosis, and older age with pulmonary findings including exudative diffuse alveolar damage, proliferative diffuse alveolar damage lung squamous metaplasia and lung viral atypia (Giugni et al. 2024).

**4.1 SARS CoV-2 and myocarditis *Myocarditis*** *and pericarditis*

Myocarditis and pericarditis in COVID-19 patients are potential post-acute cardiac sequelae of SARS-CoV-2 infection, arising from adaptive immune responses (Tuvali et al. 2022; Smer et al. 2023 ). It is common knowledge that SARS-CoV-2 infection may lead to myocardial injury, acute viral myocarditis and pericarditis (Ho et al. 2020; Siripanthong et al. 2020; Patone et al. 2022; Castiello et al. 2022; Sawalha et al. 2021; Liu et al. 2021a; Daniels et al. 2021; Caforio et al. 2022; Gnecchi et al. 2020; Yokoo et al. 2020; Chiu et al. 2020; Warchoł et al. 2020; Fairweather et al. 2023), or even fulminant myocarditis (Chen et al. 2020a; Hu et al. 2021; Zeng et al. 2020; Kitsou et al. 2024).^56-58^ The incidence of myocarditis/pericarditis after COVID-19 was at least 15 times higher than that before COVID-19. The prevalence of myocarditis before COVID-19 was 1 to 10 cases/100,000 people, while that after COVID-19 was 150 to 4,000 cases/100,000 individuals (Fairweather et al. 2023). In a total of 176,137 hospitalizations with confirmed COVID-19-infection in Germany, 226 (0.01%) had myocarditis (incidence: 1.28 per 1000 hospitalization-cases). Independent risk factors for myocarditis in COVID-19 were age < 70 years, male, pneumonia, and multisystemic inflammatory COVID-19 infection. Myocarditis was independently associated with increased case-fatality (Keller et al. 2023).There were a number of case reports of myocarditis associated with COVID-19 (Ho et al. 2020; Siripanthong et al. 2020; Patone et al. 2022; Castiello et al. 2022; Sawalha et al. 2021; Liu et al. 2021a; Daniels et al. 2021; Caforio et al. 2022; Gnecchi et al. 2020; Yokoo et al. 2020; Chiu et al. 2020; Warchoł et al. 2020; Chen et al. 2020a; Hu et al. 2021; Zeng et al. 2020).^44-58^ Therefore, it is speculated that myocarditis may be an important reason of acute heart injury in patients with COVID-19. However, the prevalence, clinical manifestations and pathogenetic mechanism of myocarditis in patients with COVID-19 are not very clear yet. In Balb/c mice, myopericarditis could be induced by inadvertent intravenous injection of COVID-19 mRNA vaccines. The mRNA expression levels of several cytokines in myocardial tissue were significantly increased from 1 dpi to 2 dpi in the intravenous group but not the intramuscular group. These cytokines included interleukin (IL)-1β, IL-6, interferon (IFN)-β, and tumor necrosis factor (TNF)-α (Li et al. 2022).^59^ In humans, COVID-19-related myocarditis may only manifest as palpitations or mild chest discomfort, which may not be differentiated with other causes of most patients. In the majority of cases, fever, shortness of breath, cough and chest pain were the most common presenting symptoms (Ho et al. 2020; Chimenti et al. 2022).^44^ In some patients, however, myocarditis could lead to fulminant disease. The cardiac and inflammatory biomarkers were elevated in most patients. Temporary ECG changes such as non-specific ST-segment and T-wave changes and ventricular tachycardia are common and may be helpful to determine the presence and severity of myocardial injury (Ho et al. 2020).^44^ Myocarditis may develop into conduction block, tachyarrhythmia and impaired left ventricular function. When myocardial injury is detected in the absence of an acute coronary syndrome, myocarditis should be highly suspected. Left ventricular dysfunction and hypokinesis was common. A diagnosis can usually make if cardiac magnetic resonance imaging (MRI) detects a typical signal of acute myocardial injury. When cardiac MRI is not feasible, cardiac computed tomographic angiography with delayed myocardial imaging may be beneficial for excluding significant coronary heart disease and identifying myocardial inflammatory patterns (Siripanthong et al. 2020).^45^ Endocardial myocardial biopsy (EMB) has been considered as the gold standard for the diagnosis of myocarditis for a long time, which can directly show myocardial necrosis and mononuclear cell infiltration. In some cases, EMB can also do virus isolation or nucleic acid test and autoimmune function test to find evidence of the cause of myocarditis. However, in the COVID-19 cases, this evidence is rare at present, and is mainly based on some case reports. During the current COVID-19 pandemic and related medical crisis, EMB may be inappropriate as a diagnostic tool. Although Some scholars believe that fulminant myocarditis may be an important clinical manifestation of the disease (Chen et al. 2020a; Hu et al. 2021),^56,57^ the exact prevalence of this serious complication is not very well understood yet. The pathophysiological mechanism of myocarditis in COVID-19 patients has been thought to be a combined role of direct viral injury (by ACE2 receptor) and the host’s hyperimmune response (Ho et al. 2020; Siripanthong et al. 2020).^44,45^ SARS-CoV-2 particles in myocardial tissue have been detected by reverse transcription-polymerase chain reaction (RT-PCR) in some cases (Yao et al. 2020).^60^ The Animal model of viral myocarditis suggests that the intrinsic immune response is activated by the release of proinflammatory cytokines in heart injury. Colzani et al. demonstrated that inflammatory cytokines (including IL-6, TNF-α, IL1-β, IL-10, CRP, and neutrophil to lymphocyte ratio with a specific reduction of CD4^+^ and CD8^+^ cells) were at least in part responsible for the cardiovascular damage seen in COVID-19 and characterise the downstream activated pathways in human cardiomyocytes (Colzani et al. 2024). ORF8, a unique accessory protein specific to SARS-CoV-2 has been shown to be a modulator of cytokine responses during SARS-CoV-2 infection. ORF8 has the capability to induce inflammatory responses (Móvio et al. 2024). Proteins released by cell lysis may exhibit similar characteristics to viral antigens and play a role by main histocompatibility complexes (MHCs). Myosin heavy chain, a kind of myocardial sarcomere protein, seems to be a main example of “molecular mimicry”. CD4+ T helper cells (Th) and cytotoxic CD8+ T cells mediate their responses by activating the inflammatory cascade and cytolysis. Macrophages can migrate to the injured site. In the final stage, there is recovery or low-level chronic inflammation and the occurrence of left ventricular failure (Blyszczuk 2019).^61^ It is worth noting that myocarditis occurs 10 to 15 days after the onset of COVID-19 symptoms. Based on the above observation results and experimental background, the core problem of potential treatment plans should be the degree of myocardial injury caused by virus replication, immune mediation or other mechanisms. Since acute myocardial injury begins 2 weeks after the onset of COVID-19 symptoms (Tajbakhsh et al. 2021),^62^ adaptive T-cell-mediated immunity or dysregulated innate effects or pathways may play a key role on the occurrence of myocarditis. In severe cases, the increase of highly proinflammatory CCR6+ Th17 in CD4+ T cells was the prominent inflammatory mediator of myocarditis. In this way, most scholars believe that a delay of myocardial inflammation is consistent with at least two pathogenic mechanisms: first, that the “cytokine storm” induces a subclinical autoimmune myocarditis; second, myocardial injury and/or molecular mimicry cause a new autoimmune response. To date, the targeted treatment plan for myocarditis is still difficult to implement. As for myocarditis in other cases, the strategy of widely supporting treatment is still the key measure. A recent case report showed that the early use of steroids and intravenous use of immunoglobulin, neuraminidase inhibitors and active mechanical life support had a top effect (Chen et al. 2020a).^56^

**4.2 SARS CoV-2 and hypertension *Hypertension***

The association between hypertension and the SARS-CoV-2 susceptibility is not completely clear. Hypertension is a common cardiovascular comorbidity in patients with COVID-19 (Harrison et al. 2021; Pellicori et al. 2021). The prevalence of hypertension among hospitalized COVID-19 patients was 15–40% in different studies ()., which was basically equivalent to the rate of hypertension in the general population (~30%) (Guan et al. 2020; Beaney et al. 2019; Mauer et al. 2022; Vosko et al. 2023).^63,64^ A previous recent analysis from China showed that hypertension was present in 13.4% of subjects with non-severe disease and in 23.7% of subjects with severe disease. A higher prevalence of hypertension was also observed in those with a poor composite outcome (Guan et al. 2020).^63^ The China CDC reported that The prevalence of hypertension was 12.8% in the whole COVID-19 patients and 39.7% in the deceased (Epidemiology Working Group for NCIP Epidemic Response, Chinese Center for Disease Control and Prevention 2020).^65^ In addition, the odds ratio (OR) of mortality in COVID-19 patients with hypertension was significantly increased by 3.05 (95% CI 1.57–5.92) (Zhou et al. 2020a).^42^ Therefore, the association between hypertension and susceptibility to SARS-CoV-2 may be largely confused by the higher incidence of hypertension in the elderly, as the prognosis is significantly poorer, the course of the disease is more severe, and the mortality rate is higher in the elderly than in young patients (Guan et al. 2020).^63^ Although these evidences are insufficient to indicate an increased susceptibility of hypertensive patients to SARS-CoV-2 (Kreutz et al. 2020), the prognosis is significantly poorer, the course of the disease is more severe, and the mortality rate is higher in the elderly than in young patients (Guan et al. 2020; Su et al. 2022). However, hypertension was associated with increased risk of various infections and post-infection complications, and increased mortality of severe infections (Su et al. 2022).^67^ The possible association between hypertension and COVID-19 may attribute to the role of ACE2 (Harrison et al. 2021). ACE2 is a key component of the rennin-angiotensin-aldosterone system (RAAS), which is closely related to the pathophysiology of hypertension (Shukla and Banerjee 2021; Vaduganathan et al. 2020). Inhibition of RAAS by angiotensin-converting enzyme inhibitors (ACEIs) or angiotensin receptor blockers (ARBs) can lead to a compensatory increase of ACE2 levels in tissues, indicating that these drugs may be harmful to patients exposed to SARS-CoV-2 (Danser et al. 2020). However, there is no clear evidence to suggest that ACEIs or ARBs lead to upregulation of ACE2 in human body (Danser et al. 2020). Therefore, there is no justification for stopping ACEIs or ARBs in patients at risk of COVID-19 (Sommerstein et al. 2020). As a matter of fact, the application of ACEIs/ARBs may be a double-edged sword in COVID-19 patients. On the one hand, it may increase the risk of SARS-CoV-2 infection. On the other hand, it may decrease the severity of lung damage caused by SARS-CoV-2 infection. In addition, SARS-CoV-2 infection may affect the balance between angiotensin II and angiotensin 1-7, while ACEI**s**/ARBs can block the RAAS and protect the heart and other organs, which are susceptible to damage caused by the RAAS activation (Guo et al. 2020a). The action of immune system is another mechanism linking hypertension and COVID-19. The function of immune system is dysregulated in hypertension and SARS-CoV-2 infection (Drummond et al. 2019; Loperena et al. 2018). Further dysregulation of the immune system was observed in patients with poor control of blood pressure. Blood lymphocyte counts were associated with human hypertension (Siedlinski et al. 2020), and CD8+ T cell dysfunction was also observed in hypertensive patients (Youn et al. 2013). CD8+ T cells cannot effectively fight against viral infection and lead to pathological overproduction of cytokines, which may be related to COVID-19. Conversely, the dysregulated immune system in hypertension can be restored after better control of blood pressure by using ACEIs or ARBs.

The possible association between hypertension and COVID-19 may attribute to the role of ACE2. ACE2 is a key component of the rennin-angiotensin-aldosterone system (RAAS), which is closely related to the pathophysiology of hypertension (Shukla and Banerjee 2021; Vaduganathan et al. 2020).^68,69^ Inhibition of RAAS by angiotensin-converting enzyme inhibitors (ACEIs) or angiotensin receptor blockers (ARBs) in animal experiments can lead to a compensatory increase of ACE2 levels in tissues, indicating that these drugs may be harmful to patients exposed to SARS-CoV-2 (Danser et al. 2020).^70^ However, there is no clear evidence to suggest that ACEIs or ARBs lead to upregulation of ACE2 in human body (Danser et al. 2020).^70^ Therefore, there is no justification for stopping ACEIs or ARBs in patients at risk of COVID-19 (Sommerstein et al. 2020).^71^ As a matter of fact, the application of ACEIs/ARBs may be a double-edged sword in COVID-19 patients. On the one hand, it may increase the risk of SARS-CoV-2 infection. On the other hand, it may decrease the severity of lung damage caused by SARS-CoV-2 infection. In addition, SARS-CoV-2 infection may affect the balance between angiotensin II and angiotensin 1-7, while ACEI**s**/ARBs can block the RAAS and protect the heart and other organs, which are susceptible to damage caused by the RAAS activation (Guo et al. 2020a).^72^

The action of immune system is another mechanism linking hypertension and COVID-19. The function of Immune system is dysregulated in hypertension and SARS-CoV-2 infection (Drummond et al. 2019; Loperena et al. 2018).^73,74^ Further dysregulation of the immune system was observed in patients with poor control of blood pressure. Blood lymphocyte counts were associated with human hypertension (Siedlinski et al. 2020),^75^ and CD8+ T cell dysfunction was also observed in hypertensive patients (Youn et al. 2013).^76^ CD8+ T cells cannot effectively fight against viral infection and lead to pathological overproduction of cytokines, which may be related to COVID-19. Conversely, the dysregulated immune system in hypertension can be restored after better control of blood pressure by using ACEIs or ARBs.

**4.3 SARS CoV-2 and arrhythmia *Arrhythmia***

Arrhythmia is common in COVID-19 patients. It can be a new-onset arrhythmia or an aggravation of a previously existing arrhythmia which indicates myocardial involvement. Liu et al. ^77^ reported that palpitation was one of their presenting symptoms in 7.3% of the COVID-19 patients (Liu et al. 2020b). Wang et al. ^2^ revealed that 16.7% of 138 hospitalized COVID-19 patients with arrhythmia, which was higher in intensive care unit (ICU) patients (44.4%) than in non-ICU patients (6.9%; Wang et al. 2020a). The prevalence of malignant arrhythmia such as hemodynamically unstable ventricular tachycardia or ventricular fibrillation was also higher in patients with high troponin than those with normal troponin levels (11.5% *vs*. 5.2%, *P* < 0.001; Guo et al. 2020b).^6^ The main types of arrhythmia in patients with COVID-19 included atrial fibrillation, atrioventricular block, ventricular tachycardia (pleomorphism, torsade de pointes) and ventricular fibrillation (Varney et al. 2022; Kochav et al. 2020).^78,79^ Bhatla et al. (2020)^80^ reported that there were 9 cases of cardiac arrest, 25 cases of atrial fibrillation, 9 cases of clinically significant slow arrhythmia, and 10 cases of non persistent ventricular tachycardia among a total of 700 patients (45% male; 71% African American) with COVID-19. Among 241 patients who received COVID-19 treatment in a tertiary hospital in Brazil (Pimentel et al. 2021),^81^ the prevalence of arrhythmia was 8.7%, and the most common arrhythmia was atrial tachyarrhythmia (76.2%). The mortality rate was higher in patients with arrhythmia than without arrhythmia (52.4% *vs*. 24.1%, *P* = 0.005). A high risk of arrhythmia was observed in patients with heart failure (hazard ratio 11.9, 95% CI 3.6-39.5, *P* < 0.001), and 3.3% of the patients experienced cardiac arrest and died during hospitalization. This indicates that the mortality rate of cardiac arrest in COVID-19 patients was very high. Interestingly, in a previous report from China, some patients mainly manifested as cardiovascular symptoms, such as palpitations and chest tightness, rather than respiratory symptoms during the initial epidemic period (Zheng et al. 2020).^8^ The potential mechanisms of arrhythmias in patients with COVID-19 have not been fully elucidated. All of SARS-CoV-2 infection-related metabolic dysfunction, myocardial inflammation, and activation of the sympathetic nervous system were associated with cardiac arrhythmia (Su et al. 2022).^67^ It is established that the immune system is implicated in the pathogenesis of cardiac arrhythmias. Auto-immune and inflammatory cardiac channelopathies may promote arrhythmias via auto-antibodies and cytokines respectively (Lazzerini et al. 2019). Inflammatory cytokines, such as tumour necrosis factor alpha (TNF-α), interleukin-1 (IL-1), and IL-6 can be arrhythmogenic and this phenomenon is observed after a systemic inflammatory response to a pathogen, including SARS-CoV-2 (Tsampasian et al. 2024). The concentration of TNF-α, IL-1, and IL-6 in patients with long COVID was substantially elevated for prolonged periods (Phetsouphanh et al. 2022; Schultheiß et al. 2022; Karbalaeimahdi et al. 2023; Melhorn et al. 2023). Thus, the possible risk factors include hypoxia, myocarditis, abnormal host immune response, myocardial ischemia, myocardial strain, electrolyte disorder, intravascular volume imbalance, metabolic disarray, sympathetic nervous system activation, hypotension, and drug side effects such as COVID-19 drug therapy and other drug interactions caused by direct pulmonary virus infection. It is worth noting that some drugs for COVID-19 therapies can prolong the QT interval and may have arrhythmogenic effects (Manolis et al. 2020; Kochi et al. 2020; Dherange et al. 2020; Yu et al. 2024).^82-84^

**4.4 SARS CoV-2 and myocardial injury and heart failure *Myocardial injury and heart failure***

Myocardial injury is very common in COVID-19 patients. It is commonly also associated with disease severity. A number of previous studies showed that the serum concentrations of lactate dehydrogenase (LDH), creatine kinase (CK) and its isoenzyme CK-MB, and high-sensitivity cardiac troponin (hs-cTn) were increased in almost all hospitalized patients with COVID-19 (Zheng et al. 2020; Huang et al. 2020; Lippi et al. 2020),^8,13,85^ or had evidence of new electrocardiographic or echocardiographic abnormalities (Huang et al. 2020; Zhou et al. 2020a).^13,42^ Approximately 10% of COVID-19 patients had heart failure, with incidence ranging from 25% to 35% in hospitalized patients (Shu et al. 2023). Heart failure was also observed in 52% of non survivors and 12% of survivors. A previous report from Wuhan, China showed that five of the first 41 patients (12%) with COVID-19 had the evidence of myocardial injury, such as elevated high-sensitivity cardiac troponin I (cTnI, > 28 pg/mL) levels (Zheng et al. 2020; Huang et al. 2020; Lippi et al. 2020),^8,13,85^ and 7.2-17% of COVID-19 inpatients had acute myocardial injury (Wang et al. 2020a; Zhou et al. 2020a).^2,42^ In another analysis of 68 death causes from Wuhan, 36 cases (53%) were respiratory failure, 5 cases (7%) were myocardial injury and circulatory failure, 22 cases (33%) were both respiratory and circulatory failures, and 5 cases (7%) were unknown cause (Ruan et al. 2020).^9^ In addition, the level of N-terminal pro B-type natriuretic peptide (NT proBNP) in COVID-19 patients was also increased in 27.5% of cases. These data indicate that myocardial injury and heart failure associated with COVID-19 are very common and should be valued by the majority of medical workers. In a previous recent report of 138 COVID-19 inpatients from Wuhan, patients treated in ICU had higher levels of biomarkers (CK-MB and hs-cTnI) of myocardial injury than those do not need ICU care (Wang et al. 2020a).^2^ In the study conducted by Zhou et al. ^42^, univariate analysis showed that cTnI level was closely related to increased mortality, but this correlation could not be detected in a multivariate model (Zhou et al. 2020a). When the cohorts was analyzed according to the need for ICU care, the similar correlation between elevated cTnI and the severity of the disease was also found (Wang et al. 2020a; Huang et al. 2020).^2,13^ Moreover, recovered COVID-19 patients showed an increased risk of incident heart failure in the same follow-up period. COVID-19 survivors had an additional 90% risk of developing heart failure after COVID-19 infection in the long-term period. This risk was directly related with age and previous history of hypertension especially in the early post-acute phase of the infection (Zuin et al. 2023b). Therefore, according to current experience and research, patient monitoring should include some laboratory indicators.

Cardiogenic shock is a critical manifestation of myocardial injury in COVID-19 patients (Shu et al. 2003). The incidence of cardiogenic shock in COVID-19 was about 0.7%. Compared to patients without shock, those with cardiogenic shock had a higher incidence of previous myocardial infarction, coronary revascularization, and heart failure, as well as abnormal chest imaging and elevated troponin, D-dimer, C-reactive protein, and natriuretic peptides on admission. The incidence of in-hospital mortality, cardiac arrest, myocardial infarction, or stroke were higher in patients with cardiogenic shock than in those without cardiogenic shock (77% *vs*. 13%; Varshney et al. 2021 ). At present, it is believed that large-scale cytokine storm induced by viral infection is the major cause of cardiogenic shock. Moderate doses of steroids can significantly improve the shock and multiple organ dysfunction of patients. Takotsubo cardiomyopathy induced by COVID-19 may manifest as cardiogenic shock (Shu et al. 2003).

At present, the exact mechanism of myocardial injury in COVID-19 patients is not fully understood. The proposed mechanisms of myocardial injury include direct damage effects of SARS-CoV-2, systemic inflammation, endothelial dysfunction, platelet activation, sympathetic activation, myocardial interstitial fibrosis, interferon mediated immune response, exaggerated cytokine response by types 1 and 2 helper T cells, vasoconstriction, hypercoagulation, hypoxemia, and COVID-19 therapy-related drugs (corticosteroids, rivabirina) (Wang et al. 2020a; Shi et al. 2020; Guo et al. 2020b; Zhou et al. 2020a; Chen et al. 2020b; Babapoor-Farrokhran et al. 2020; Xu et al. 2020b; Varga et al. 2020; Klok et al. 2020).^2,5,6,42,86-90^

Due to the high expression of ACE2 in the cardiovascular system, especially in the failed human heart (Chen et al. 2020b)[86], SARS CoV-2 may directly infect myocardial cells, which seems to explain the higher viral infection and mortality rates in heart failure patients. The high inflammatory state and cytokine release induced by virus infection may lead to vascular and myocardial inflammation, plaque instability, hypercoagulability, and even directly inhibit the myocardium. In this case, the “cytokine storm” caused by immune imbalance may be a key modulator (Zheng et al. 2020).^8^ The concentration of plasma IL-6 in COVID-19 patients with myocardial injury was increased (Chen et al. 2020a),^56^ and there were many abnormal cytokines in COVID-19 patients. TNF-α and IL-6 are known to be implicated in the pathophysiology of myocardial infarction, inflammation, and heart failure (Schumacher and Naga Prasad 2018; Hanna and Frangogiannis 2020). Other complications such as sepsis and disseminated intravascular coagulation (DIC) may also lead to myocardial injury. In addition, patients with long COVID have been shown to have auto-antibodies specifically against components of the cardiovascular system, including anti-cardiolipin and anti-apolipoprotein A-1 antibodies, both of which are linked with cardiovascular events and worse outcomes (Dobrowolska et al. 2023).

Endothelial cell dysfunction is closely associated with the development of many cardiovascular diseases, such as atherosclerosis, CHD, and hypertension. Numerous studies have shown that SARS CoV-2 infection can cause serious cardiovascular system complications and that patients with pre-existing cardiac disease have a higher incidence of adverse events and mortality after infection with SARS CoV-2, with endothelial damage being an important part of the pathogenesis (Rossouw et al. 2022). Histological and pathological findings of patients who died from COVID-19 suggested the presence of endothelial inflammation, degradation of endothelial cells, and viral structures in endothelial cells were observed in multiple organs throughout the body (Fodor et al. 2021). The pathological mechanism of endothelial cell structure and dysfunction caused by SARS-CoV-2 is complex. First, the virus itself attacks and damages endothelial cells. S protein disrupts endothelial cell integrity, and N protein induces a pro-inflammatory cell phenotype that triggers the release of inflammatory factors and cytokines by binding to TLR2 in endothelial cells, triggering the NF-κB and MAPK signaling pathways (Qian et al. 2021). Both of these proteins drive viral-mediated endothelial injury. In addition, S protein can activate the alternative pathway of complement to increase endothelial cytotoxicity and activate NLRP3 present in vascular endothelial cells, leading to endothelial cell dysfunction (Rossouw et al. 2022). SARS-CoV-2 can also indirectly damage endothelial cells through oxidative stress (Fodor et al. 2021). SARS-CoV-2 can induce activation of NADPH-oxidase and promote superoxide (O_2_^−^) production, which leads to mitochondrial damage (Fodor et al. 2021). Damaged mitochondria in COVID-19 can promote β-oxidation of fatty acids in vascular endothelial cells to increase oxidative stress (Montiel et al. 2022). In addition, oxidative stress can promote the oxidation of thiols in SARS-CoV-2 and SARS-CoV-2 proteins to disulfides, increasing viral binding to ACE2 and thus aggravating the infection (Hati and Bhattacharyya 2020). Therefore, the reduction of oxidative stress is an essential component for the intervention and control of the recent and long-term complications of SARS-CoV-2 infection. 3) Increased expression of adhesion molecules and platelet aggregation occur when severe infections lead to endothelial cell damage (Rossouw et al. 2022). It was found that some patients with SARS-CoV-2 infection have different types of antibodies in their sera that activate endothelial cells to increase the expression of surface adhesion molecules such as intercellular adhesion molecule-1, E-selectin, and vascular cell adhesion molecule-1, increasing the incidence of adverse thrombotic events (Shi et al. 2022). Meanwhile, Toll receptor 7 (TLR7) on the platelet surface during SARS-CoV-2 infection binds to the single-stranded RNA of SAR2-CoV-2, accelerating endothelial damage and leading to increased thrombotic susceptibility (Rossouw et al. 2022). Endothelial cell injury can cause excessive platelet stress, and the interaction between the two disrupts the pre-existing homeostatic balance of the vasculature, thereby causing microvascular occlusion, cellular oxidative stress, and the release of pro-thrombotic/pro-coagulant factors (Rossouw et al. 2022). In addition, endothelial cell dysfunction can be secondary to inflammatory responses and increased vascular permeability, leading to the development of myocardial edema and myocarditis (Prasad et al. 2021; Rossouw et al. 2022).

Many drugs for COVID-19 treatments may induce cardiac toxicity. Currently, the treatment measures for myocardial injury caused by SARS CoV-2 include steroids, immunoglobulin, hydroxychloroquine and other antiviral drugs, as well as various life support therapies (Chen et al. 2020a).^56^ Although it is uncertain whether these treatments can successfully limit myocardial injury, the detection of cardiac injury indicators in hospitalized patients with COVID-19 may help to determine the risk of complications.

**4.5 SARS CoV-2 and coronary heart disease *Coronary heart disease***

SARS CoV-2 infection has been associated with a higher incidence of acute myocardial infarction and related complications (Nanavaty et al. 2024). COVID-19 is a risk factor for acute myocardial infarction, which may represent a part of the clinical picture of COVID-19 (Katsoularis et al. 2021). ST-segment elevation myocardial infarction (STEMI) is one of the fatal complications following COVID-19 (Gharibzadeh et al. 2023). According to available data, 2.5-15% of COVID-19 patients had CHD (Wang et al. 2020a; Chen et al. 2020c; Guan et al. 2020). In a previous analysis of 191 patients, 15 (8%) of COVID-19 patients had CHD (Zhou et al. 2020a). Recent studies and meta-analysis revealed that COVID-19 patients with CHD were associated with poor prognosis (Liang et al. 2021; Gharibzadeh et al. 2023; Majeed et al. 2023; Baytuğan et al. 2024; Dogan et al. 2024). Majeed et al. revealed that ST-segment elevation myocardial infarction (STEMI) patients with COVID-19 had higher inpatient mortality, increased length of stay and higher cost of hospitalization when compared to STEMI patients without COVID-19. STEMI patients with COVID-19 also received significantly less invasive cardiac procedures such as coronary angiograms,  percutaneous coronary intervention, and coronary artery bypass grafting CABG) and were more likely to receive systemic thrombolytic therapy when compared to STEMI patients without COVID-19 (Majeed et al. 2023). The mortality and adverse consequences of STEMI in patients with COVID-19 were also far higher than in the general population (Gharibzadeh et al. 2023). STEMI patients with concomitant COVID-19 or with a history of SARS CoV-2 infection were associated with increased major adverse cardiac events (MACE; Dogan et al. 2024; Baytuğan et al. 2024). These results suggest that CHD is a risk factor for poor prognosis of COVID-19 patients. Currently, the mechanisms of SARS CoV-2 infection induced acute myocardial infarction, especially non ST-T elevation acute myocardial infarction include: 1) SARS CoV-2 infection can cause systemic inflammatory response syndrome, cytokine storm and immune response that increase the risk of plaque rupture and thrombus formation, resulting in either an ST-elevation or non-ST-elevation myocardial infarction (Kang et al. 2020; Musher et al. 2019; Cole et al. 2018). 2) SARS CoV-2 infection can also reduce the oxygen delivery to myocardium via hypoxemia and vasoconstriction, as well as the hemodynamic effects of sepsis with increased myocardial oxygen demand. This supply-demand mismatch may lead to sustained myocardial ischemia in patients with underlying CHD (Kang et al. 2020). 3) DIC was found in 0.6% of survivors and 71.4% of non-survivors with COVID-19 (Tang et al. 2020). DIC was associated with coronary artery (epicardial and microvascular) thrombosis, focal myocardial necrosis, and severe cardiac dysfunction (Wang et al. 2020c; Hakobyan et al. 2023). 4) Myocardial injury of COVID-19 may also occur through non-ischaemic mechanisms, such as acute and fulminant myocarditis and stress-induced cardiomyopathy (Zeng et al. 2020; Fried et al. 2020; Sala et al. 2020; Arentz et al. 2020; Inciardi et al. 2020). 5) SARS-CoV-2 can cause the immune system to strongly release various cytokines and chemokines (IL-1, IL-6, T helper 1 cytokine IFN-γ, and TNF-α; cytokine release syndrome or cytokine storm). These proinflammatory cytokines may immediately inhibit myocardial function by activating the neural sphingomyelinase pathway (Ruan et al. 2020; Huang et al. 2020; Mehta et al. 2020; Mann 2015). Besides, cytokine storm may lead to extensive endothelial dysfunction, serious microvascular dysfunction, and induce non obstructive coronary myocardial infarction (Chen et al. 2020b).

Although report of type I myocardial infarction (induced by plaque rupture or thrombosis) in COVID-19 patients has yet been published, rumoured report was existent (Kang et al. 2020).^91^ In previous SARS and MERS cases, two of the five deaths reported earlier were accompanied by acute myocardial infarction (Peiris et al. 2003).^92^ Similar manifestations were also observed in COVID-19 patients. According to available clinical data, 2.5-15% of COVID-19 patients had coronary heart disease (Wang et al. 2020; Chen et al. 2020c; Guan et al. 2020).^2,33,63^ In a recent analysis of 191 patients from Wuhan, 15 (8%) of COVID-19 patients had coronary heart disease (Zhou et al. 2020a).^42^ A recent meta-analysis revealed that coronary heart disease was associated with poor prognosis of COVID-19. After subgroup analysis, the authors found that coronary heart disease was associated with mortality, severe/critical COVID-19, ICU admission, and disease progression. Meta regression analysis found that the correlation between the adverse prognosis of coronary heart disease and COVID-19 was influenced by hypertension (Liang et al. 2021).^93^ These results suggest that coronary heart disease is a risk factor for poor prognosis of COVID-19 patients. Currently, the mechanism of SARS CoV-2 induced coronary heart disease, especially non ST-T elevation myocardial infarction, is not fully understood. However, several pathways related to viral diseases may lead to plaque instability in COVID-19 patients (Musher et al. 2019).^94^ Viral infection can potentially destroy the stability of atherosclerotic plaque through systemic inflammatory response, cytokine storm and immune response (Cole et al. 2018).^95^ 1) Severe viral infections can cause systemic inflammatory reactions, increase the risk of plaque rupture and thrombosis, lead to either ST segment elevation or non ST segment elevation (type I) myocardial infarction (Warren-Gash et al. 2016).^96^ 2) Severe viral infections can also reduce the oxygen delivery to myocardium via hypoxaemia and vasoconstriction, as well as the haemodynamic effects of sepsis with increased myocardial oxygen demand. This supply-demand mismatch may lead to sustained myocardial ischemia in patients with underlying coronary artery disease (type II myocardial infarction) (Kang et al. 2020).^91^ 3) DIC was found in 0.6% (1/162) of survivors and 71.4% (15/21) of non-survivors with COVID-19 (Tang et al. 2020).^43^ DIC was associated with coronary artery (epicardial and microvascular) thrombosis, focal myocardial necrosis, and severe cardiac dysfunction (Sugiura et al. 1977; Wang et al. 2020b).^97,98^ 4) Myocardial injury of COVID-19 may also occur through non-ischaemic mechanisms, such as acute and fulminant myocarditis and stress-induced cardiomyopathy (Zeng et al. 2020; Fried et al. 2020; Sala et al. 2020; Arentz et al. 2020; Inciardi et al. 2020).^58,99,100-102^ 5) SARS-CoV-2 can cause the immune system to strongly release various cytokines and chemokines (Huang et al. 2020; Mehta et al. 2020).^13,103^ This phenomenon is also called cytokine release syndrome (cytokine storm), an immunopathological process induced by excessive induction of proinflammatory cytokines such as IL-1, IL-6, T helper 1 cytokine IFN-γ, and TNF-α. These proinflammatory cytokines may immediately inhibit myocardial function by activating the neural sphingomyelinase pathway (Ruan et al. 2020; Huang et al. 2020; Mehta et al. 2020; Mann et al. 2015).^9,13,103,104^  Besides, cytokine storm may lead to extensive endothelial dysfunction, serious microvascular dysfunction, and induce non obstructive coronary myocardial infarction (Chen et al. 2020b).^86^

***Stress cardiomyopathy***

Stress cardiomyopathy (Takotsubo syndrome) is a life-threatening transient left ventricular dysfunction triggered by either physical or emotional stressors (Singh et al. 2023). It has become a well-known complication of SARS-CoV-2 infections (Davis et al. 2023). During COVID-19 pandemic, emotional and physical distress indued by strict social distancing rules, self-isolation, quarantine, economic and social stress, fear of virus infection may be the cause to trigger the takotsubo syndrome (Burger et al. 2021). A significant increase in the incidence rate of takotsubo syndrome has been found dring COVID-19 pandemic (Hajra et al. 2023). Hajra et al. found that the in-hospital outcomes in patients with stress cardiomyopathy and concurrent COVID-19 infection with those without COVID-19 infection were significantly different. In a total of 41,290 hospitalizations for stress cardiomyopathy (1665 patients with of COVID-19), the incidence of complications, including acute kidney injury, acute kidney injury requiring dialysis, coagulopathy, sepsis, cardiogenic shock, cases with prolonged intubation of > 24 h, requirement of vasopressor and inpatient mortality, were significantly higher in patients with COVID-19. Concomitant COVID-19 infection was independently associated with worse outcomes and increased mortality in patients hospitalized with stress cardiomyopathy (Hajra et al. 2023). In a total of 1,659,040 patients, there were COVID-19 with stress cardiomyopathy (*n* = 1,665; 0.1%) and COVID-19 without stress cardiomyopathy (*n* = 1,657,375; 99.9%). COVID-19 patients with stress cardiomyopathy had significantly increased in-hospital mortality compared to COVID-19 patients without stress cardiomyopathy (32.8% *vs*. 14.6%, *P* = 0.01) along with significantly increased mechanical ventilation and vasopressor support, hospitalization charge, acute kidney injury requiring hemodialysis, cardiogenic shock, and cardiac arrest. These results emphasize the need for more research to reduce worse outcomes with COVID-19-related stress cardiomyopathy patients (Davis et al. 2023).

***Ischemic stroke***

There is increasing evidence that COVID-19 is associated with ischemic stroke (Finsterer et al. 2022; Ozcan et al. 2023; De Michele et al. 2023). An increasing number of COVID-19 patients with ischemic stroke has been reporting (Luo et al. 2022; Zuin et al. 2023a). A systematic review and meta-analysis including 26,691 participants and 280 patients with ischemic stroke and COVID-19 showed that the pooled prevalence of ischemic stroke in COVID-19 was 2% (95% CI 1-2%; *P* < 0.01). The pooled proportions of hypertension, hyperlipidemia and diabetes in COVID-19-related ischemic stroke was 66%, 48% and 40% (*P* < 0.01 for all), respectively (Luo et al. 2022). Another systematic review and meta-analysis including 23,559,428 patients (1,595,984 COVID-19 patients) revealed that ischemic stroke occurred in 4.40 out of 1000 patients survived to COVID-19 compared to 3.25 out of 1000 controls (Over a mean follow-up of 9.2 months). Recovered COVID-19 patients presented a higher risk of ischemic stroke compared to people who did not have COVID-19. COVID-19 patients hospitalized at the time of the infection have a subsequent higher risk of stroke during the follow-up compared to those non-hospitalized (Zuin et al. 2023a). Moreover, a recent study demonstrated that COVID-19 extends the infarct volume during acute ischemic stroke, one of the feared complications of COVID-19 (De Michele et al. 2023). COVID-stroke occurs in all age groups and predominantly in males. The anterior circulation is more frequently affected than the posterior circulation (Finsterer et al. 2022). The mechanisms underlying ischemic stroke are thought to be driven by multiple pathophysiological factors, including hypercoagulation, microthrombosis, and endothelial dysfunction (De Michele et al. 2023). Immune-mediated thrombosis, the renin angiotensin system and the effect of SARS-CoV-2 in cardiac and brain tissue may also contribute to the pathogenesis of ischemic stroke in patients with COVID-19 (Sagris et al. 2021). In addition, certain studies have suggested that COVID-19 induces acute ischemic stroke by promoting hypercoagulability. These patients often had an abnormal coagulation, namely, elevated levels of D dimer and fibrinogen, and a low platelet count. Nevertheless, the exact mechanisms through which COVID-19 leads to a hypercoagulable state in infected patients remain unclear (Zhang et al. 2021).

4.6  **SARS CoV-2 and blood coagulation abnormalities *Blood coagulation abnormalities***

COVID-19 is also associated with thromboembolic disease, and increases the risk of venous and arterial thromboembolism events (Tomasoni et al. 2020; Zhou et al. 2020a; Tang et al. 2020; Ali and Spinler 2021; Danzi et al. 2020; Wichmann et al. 2020; Wichmann 2020; Gąsecka et al. 2021; Kyriakoulis et al. 2021; Heinrich et al. 2022; Marvi et al. 2022; Tsaplin et al. 2021; Farkouh et al. 2022; Epelbaum 2020; Takasu et al. 2022; Hendren et al. 2020).^4,42,43,105-117^ This relationship has attracted widespread attention in the scientific community (Ali and Spinler 2021).^105^ Both DIC and pulmonary embolism were very common in COVID-19 patients. DIC was found in 71.4% of non survivors (Tang et al. 2020).^43^ A large number of pulmonary embolisms were also reported in COVID-19 patients (Danzi et al. 2020; Hobohm et al. 2023).^106^ In Germany, the fatality rate among patients with both COVID-19 and pulmonary embolism was substantially higher than that in those with only one of these diseases, suggesting a life-threatening additive prognostic impact of the COVID-19-pulmonary embolism combination (Hobohm et al. 2023). The incidence of deep venous thrombosis in COVID-19 patients was 22.7% by ultrasound examination (Shi and Fu 2020)^118^ and 27% in ICU patients (Klok et al. 2020).^90^ A previous recent autopsy report by German scholars showed that 7 of the 12 patients (58%) had deep vein thrombosis, which was not suspected before death. The direct cause of death in four patients was pulmonary embolism. Reticular infiltration of the lungs with severe bilateral dense consolidation was found by autopsy computed tomography scan, while 8 patients had histomorphologically diffuse alveolar injury. High concentration of SARS-CoV-2 RNA was detected in the lungs, and high viral RNA titers in the liver, kidney, or heart were also determined in 6 of 10 and 5 of 12 patients (Wichmann et al. 2020; Wichmann 2020).^107,108^ Gąsecka et al. (2021)^109^ revealed that 1 of 3 inpatients with severe COVID-19 had macrovascular thromboembolic complications, including venous thromboembolism, myocardial injury/infarction, and stroke. Meanwhile, the autopsy series showed consistent patterns of multiple organ damage and microvascular damage (Gąsecka et al. 2021).^109^ Microthrombosis mainly occurs in the pulmonary vascular system, but it may also occur in other organs (Kyriakoulis et al. 2021).^110^ Heinrich et al. (2022)^111^ also showed that venous thromboembolism in critical COVID-19 patients was 17% of ante mortem and 38% of postmortem. Incidence rate of postmortem venous thromboembolism was higher in COVID-19 (43%) than in age- and sex-matched non-COVID-19 (0%) cohorts (*P* = 0.001). In a recent study, Marvi et al.^112^ found that the maximum amplitude of thromboelastogram was related to the occurrence of venous thromboembolism in critical COVID-19 patients. For every 1 mm increase in enrollment and peak maximum amplitude, the risk of venous thromboembolism was reduced by 8% and 14%, respectively. Lower enrollment platelet counts and fibrinogen levels were also associated with an increased risk of venous thromboembolism. Platelet counts and fibrinogen levels were positively associated with maximum amplitude. The association between diminished maximum amplitude, platelet counts, fibrinogen and venous thromboembolism may suggest a relative consumptive coagulopathy in critical COVID-19 patients (Marvi et al. 2022). Recently, the study by Tsaplin et al. ^113^ found that there was a significant correlation between the Caprini score and the risk of venous thromboembolism in COVID-19 patients. All of eight models (eight different versions) including specific COVID-19 scores showed equally high predictability, and use of the original Caprini score was appropriate for COVID-19 patients (Tsaplin et al. 2021). These results indicate that coagulation abnormality induced by COVID-19 plays an important role in the high incidence of thromboembolic events. Clinical experience showed that coagulopathy associated with COVID-19 had obvious characteristics, including markedly elevated D-dimers concentration (Gąsecka et al. 2021).^109^ The increase of D-dimer highly predicted the adverse consequences of COVID-19. A retrospective and multicenter cohort study showed elevated levels of D-dimer (> 1 μg/L) was closely related to hospitalization mortality rate (Zhou et al. 2020a).^42^ Heinrich et al. (2022)^111^ showed that the change of anticoagulation practice was related to the statistically significant prolongation of survival time (HR = 2.55, 95%CI = 1.41-4.61, *P* = 0.01) and reduced the occurrence of venous thromboembolism (54% *vs*. 25%; *P* = 0.02). Some studies also support that anticoagulant therapy may play a role in patients who do not need ICU support (Gąsecka et al. 2021; Kyriakoulis et al. 2021; Farkouh et al. 2022).^109,110,114^ However, due to the lack of guidance on determining the strength and duration of anticoagulation, decisions should be made based on specific circumstances (Gąsecka et al. 2021).^109^ At present, the pathophysiolocal mechanisms have not been fully explored. The independent risk factors for venous thromboembolism were increasing age, males, long interval from symptom onset to admission, low fibrinogen, increased activity of factor V, high D-dimer levels on admission, and D-dimer increment ≥ 1.5 times (Wu et al. 2021; Shu et al 2023). Endothelial injury that induces tissue factor and platelet activation, low fibrinolysis, and pro-inflammatory cytokines that promote microvascular damage have been implicated in the thrombotic process, and involved in the development of venous thromboembolism due to COVID-19 (Shu et al 2023). The high inflammatory load associated with COVID-19 seems to be associated with coexisting coagulopathy (Kyriakoulis et al. 2021).^110^ Inflammation may occur in endothelial cells. Significant inflammation with endotheliitis can also lead to disseminated intravascular coagulation, thrombosis of small vessels or great vessels with tissue necrosis or infarction (Tomasoni et al. 2020; Hendren et al. 2020).^4,117^

***Dyslipidemia***

SARS-COV-2 infection is linked with the development of cardio-metabolic disorders, including dyslipidemia (Al-Kuraishy et al. 2023). Cholesterol may play a central role of the SARS-COV-2 infection. An elevated cholesterol concentration has been suspected to increase the susceptibility for SARS-COV-2 infection. Conversely, higher high-density lipoprotein cholesterol (HDL-C) levels seem to have protective action (Julius et al. 2022). Higher antecedent serum HDL-C levels were associated with a lower SARS-CoV-2 infection risk (Chidambaram et al. 2022a). Lower HDL-C levels correspond with a higher susceptibility to SARS-CoV-2 infection in general, while higher HDL-C levels were related to a lower risk of SARS-CoV-2 infection (Kowalska et al. 2022). Elevated triglyceride (TG) levels in COVID-19 patients may be considered an indicator of uncontrolled inflammation and an increased risk of death because TG levels were significantly higher in non-surviving severe patients than in surviving mild patients (Kowalska et al. 2022). However, the changes in serum lipid levels are inconsistent in COVID-19 patients. The levels of total cholesterol (TC) were significantly lower in COVID-19 patients than in healthy controls (Chidambaram et al. 2022b; Wang et al. 2020b). The concentrations of TG were lower (Wang et al. 2020b) or higher (Julius et al. 2022; Kowalska et al. 2022) in COVID-19 patients than in healthy controls, but did not differ based on COVID-19 severity or mortality (Chidambaram et al. 2022b). Serum HDL-C (Chidambaram et al. 2022b; Julius et al. 2022; Kowalska et al. 2022; Wang et al. 2020b) and LDL-C (Chidambaram et al. 2022a; Kowalska et al. 2022; Wang et al. 2020b) levels were significantly lower in COVID-19 patients than in healthy controls. Lipoprotein(a) may increase during COVID-19 and is most probably responsible for thromboembolic events. This lipoprotein can induce a progression of atherosclerotic lesion formation (Julius et al. 2022). A decrease in apolipoprotein A1 was associated with increased clinical severity in COVID-19 (Mietus-Snyder et al. 2022). In addition, other lipid particles, including total, large, and small HDL particles, as well as HDL functional cholesterol efflux capacity, were related to the severity of COVID-19 among pediatric patients (Mietus-Snyder et al. 2022). Severe COVID-19 patients had lower TC, LDL-C, and HDL-C at admission compared to patients with non-severe disease. Deceased patients had lower TC, LDL-C and HDL-C at admission (Chidambaram et al. 2022b). Wang et al. also found lower levels of HDL-C in severe COVID-19 patients than in non-severe patients. Moreover, patients with low HDL-C at admission showed a higher risk of developing severe events compared with those with high HDL-C (Wang et al. 2020b). A direct correlation was found between a decrease in serum cholesterol, HDL-C, LDL-C and TGs concentrations and the severity of the disease. These laboratory findings may serve as potential markers for patient outcomes (Kowalska et al. 2022). The pathogenic mechanisms of dyslipidemia in COVID-19 patients are not fully elucidated. The possible mechanisms are as follows: 1) Cholesterol is necessary for SARS-CoV-2 to enter the host cells, and membrane cholesterol increases the number of viral entry sites on the host cell membrane and the number of ACE2 receptors in the membrane fusion site (Tang et al. 2021). SARS-CoV-2 binds HDL-C, then this complex is attached to the co-localized receptors, facilitating viral entry (Al-Kuraishy et al. 2023). 2) SARS-CoV-2 infection may induce the development of dysfunctional HDL-C through different mechanisms, including induction of inflammatory and oxidative stress with activation of inflammatory signaling pathways. In turn, the induction of dysfunctional HDL-C induces the activation of inflammatory signaling pathways and oxidative stress, increasing COVID-19 severity (Al-Kuraishy et al. 2023). 3) Cholesterol in blood interacts with the S protein to promote the entry of spike cells, wherein the scavenger receptor class B type 1 plays an important role (Tang et al. 2021). 4) APOE binding to ACE2 attenuates the interaction of ACE2 with S protein, inhibits SARS-CoV-2 pseudovirus infection, and attenuates the inflammatory response. However, the inhibitory effect of APOE4 was lower due to different conformational structures (Zhang et al. 2022). 5) Patients carrying the APOEε4 gene have a higher susceptibility to SARS-CoV-2 and increased serum inflammatory factors (Zhang et al. 2022). Because of the cardiovascular protective effects and the additional anti-inflammatory effects of lipid-lowering drugs, it is currently recommended to continue lipid-lowering therapy for patients with COVID-19 (Tang et al. 2021; Julius et al. 2022). Statins, the 3-hydroxy-3-methylglutaryl coenzyme A reductase (HMG-CoA) inhibitors, have cholesterol-lowering, anti-inflammatory, antithrombotic, and antioxidant effects. Kouhpeikar et al. demonstrated that statins decreased the composite outcomes of mortality, ICU admissions, and intubations among COVID-19 patients (Kouhpeikar et al. 2022). Statin treatment also lowered inflammatory markers such as C-reactive protein (CRP) levels and neutrophil counts. These findings suggest a potential antiinflammatory role of statins in mitigating the composite adverse outcomes associated with COVID-19 (Ozcan et al. 2023). Preclinical and clinical studies indicated a potential therapeutic role of apolipoproteins and agents targeting them in COVID-19. One of the potential mechanistic hallmarks underlying the benefits of apolipoproteins is suggested to be protection against COVID-19-induced endothelial dysfunction (Ozcan et al. 2023). Some scholars, however, believe that lipid-lowering treatment should be carried out with caution, as plasma LDL-C levels may have a dual impact on COVID-19 patients, similar to a double-edged sword (Ozcan et al. 2023).

**5 | THERAPY STRATEGIES OF COVID-19 Therapy strategies of COVID-19**

Early diagnosis of SARS CoV-2 infection is crucial for the recommendation of appropriate treatment strategy and to address associated cardiovascular complications. To minimize cardiovascular complications in COVID-19 patients, the COVID-19 patients require routine monitoring of cardiac parameters with echocardiography, telemetry to assess QT interval and electrocardiograph to identify the occurrence of cardiovascular complications (Samidurai and Das 2020). There are currently no specific treatment methods for the SARS-CoV-2 infection, although many are under investigation. In addition, treatment of COVID-19 should be personalized according to host characteristics, degree of severity and available treatment options (Lui and Guaraldi 2023). The proposed therapy approaches are summarized as follows (Lu 2020a; Morse et al. 2020):^119,120^ 1)

*Supportive therapies*

oxygen therapy, extracorporeal membrane oxygenation (ECMO), intraaortic balloon pump (IABP) and so on. Oxygen therapy is the choice for patients with severe respiratory infections, respiratory distress, hypoxemia or shock. Respiratory support should be given to patients with hypoxic respiratory failure and acute respiratory distress syndrome. Extracorporeal Membrane Oxygenation (ECMO) should be considered for the patients with refractory hypoxemia that is difficult to be corrected by protective lung ventilation (Jin et al. 2020).

2) Antiplatlet and anticoagulant therapies

All P2Y_12_ receptor inhibitors can reduce platelet-leukocyte aggregates and platelet-derived pro-inflammatory cytokines. Evidences suggested that prehospital use of aspirin was associated a lower risk of for developing acute respiratory distress syndrome and mortality in pneumonia patients. Ticagrelor has potent anti-inflammatory properties via dual inhibition of platelet P2Y_12_ receptor and equilibrative nucleoside transporter 1, which inhibits cellular adenosine uptake. Ticagrelor also has encouraging clinical benefit in the management of pneumonia by preventing the complications of sepsis and reducing lung injury (Su et al. 2020). Zhou et al. suggested among COVID-19 patients who are currently on antiplatelet therapy, maintaining P2Y_12_ inhibitor monotherapy such as ticagrelor may be scientifically reasonable for patients with PCI performed ≥3 months (Zhou et al. 2020b; Su et al. 2020). Blood coagulation abnormalities such as venous and arterial thromboembolism, pulmonary embolism, DIC, and microvascular thrombosis/occlusion are known to occur in the majority of COVID-19 patients. Therefore, anticoagulant therapy (unfractionated heparin or low molecular weight heparin) in some COVID-19 patients is necessary. Anticoagulant therapy with direct oral anticoagulation was proven to be effective in reducing the mortality risk in patients with myocardial injury in patients after non-cardiac surgery, but the effect on the myocardial damage in COVID-19 is still unexplored (Nuzzi et al. 2022). In a study including 449 severe COVID-19 patients, anticoagulant therapy using low molecular weight heparin was associated with lower mortality in the subpopulation meeting sepsis-induced coagulopathy criteria or with markedly elevated D-dimer. These findings suggest that all hospitalized COVID-19 patients should receive thromboprophylaxis, or full therapeutic-intensity anticoagulation if indication is present (Bikdeli et al. 2020; Barrett et al. 2020).

*Immunosuppressive therapies*

Immunosuppressive therapies (including immunesuppression and immunomodulation): glucocorticoids (prednisone, hydrocortisone, methylprednisolone); IL-6 receptor inhibitors [tocilizumab (an antihuman IL-6 receptor monoclonal antibody), sarilumab and siltuximab]; convalescent serum or plasma (viral neutralization, specific immunoglobulin G antibody); IFN-β (Has immunomodulatory properties); cyclosporine, azathioprine; and immunomodulation (intravenous immunoglobulins, monoclonal antibodies targeting IL-6 or IL-6 receptor). Vaccination has been shown to reduce the risk of cardiac injury (Parodi et al. 2023) and prevent Long COVID syndrome (Antonelli et al. 2022). A meta-analysis has already shown that vaccinated individuals have 40% less risk to develop Long COVID compared to unvaccinated people (Tsampasian et al. 2023). Another systematic review and meta-analysis of six studies and 629 093 patients showed that patients with two-dose vaccination had 36% and 40% less risk of Long COVID compared to those with no or one-dose vaccination (Watanabe et al. 2023).

Convalescent plasma therapy (plasma containing the antibody from recovered patients infected with COVID-19) and monoclonal antibody therapy have been evaluated with some moderate success. Studies showed that convalescent plasma therapy was effective, and the level of neutralizing increased as high as 1:640 times in patients with SARS-CoV-2 infection [25]. Monoclonal antibody can target the specific epitope on the spike protein of SARS-CoV-2 and block the virus entry in to the host cells (Hoffmann et al. 2020; Samidurai and Das 2020).

*Antiretroviral therapy*

4) Antiretroviral therapy: Atazanavir (inhibited SARS-CoV-2 replication and proinflammatory cytokines), liponovir/ritonavir (A combination drug also called Kaletra, to inhibit the protease of HIV; and to bind SARS-CoV-2 3C-like proteinase and consequently suppress its replication) (protease inhibitors), remdesivir (reducing viral replication and binding to the active site on RNA polymerase of SARS-CoV-2), ivermectin (causes an influx of Cl ions through the cell membrane, leading to hyperpolarization of ion channels and muscle paralysis), lopinavir, oseltamivir, arbidol, favipiravir (Competitive inhibition of the RNA-dependent RNA polymerase), ribavirin (An RNA-dependent RNA polymerase inhibitor), nirmatrelvir [inhibits viral replication by targeting the chymotrypsin–like cysteine protease enzyme (Tsampasian et al. 2024; Adu-Amankwaah 2024)]. molnupiravir, camostat mesylate (serine protease inhibitor, inhibit SARS-CoV entry into cells), chloroquine (antimalarial drug), hydroxychloroquine (rheumatoid arthritis and systemic lupus erythematosus treatment), and azithromycin. Antivirals that are recommended for the acute COVID-19 infection in patients with high-risk features have also been shown to be beneficial (Xie et al. 2023b; Xie et al. 2023d; Wan et al. 2023; Butler et al. 2023). A recent retrospective cohort study that included 281,793 participants, showed that nirmatrelvir reduced the risk of Long COVID syndrome by 26% and the risk of post-acute death and hospitalization by 47 and 24%, respectively (Xie et al. 2023c; Tsampasian et al. 2024). Large cohort studies demonstrated that the use of nirmatrelvir and molnupiravir during the acute illness significantly reduced the incidence of Long COVID syndrome and the post-acute COVID-19 sequalae (Fung et al. 2023; Xie et al. 2023a).

*Cell-based therapies*

5) Cell-based therapies of heart disease (Some clinical studies support the notion that cell therapy can attenuate inflammation, which may be attractive in COVID-19) (Marbán 2018):^121^ skeletal myoblasts, bone marrow mononuclear cells, mesenchymal stem cells (MSCs, are somatic progenitor cells that possess immunomodulatory properties), mesenchymal precursor cells, CD34 + cells, cardiopoietic cells, and cardiosphere-derived cells (CDCs, are stromal progenitor cells) (Leng et al. 2020; Liang et al. 2020).^122,123^ Recently, stem cell therapies with secreted extracellular vesicles (EVs) offer a potential therapeutic benefit in COVID-19 patients by attenuating inflammation with regeneration of the damaged lung. Mesenchymal stem cells (MSCs)-derived extracellular vesicles-based therapy could be the most promising reparative strategy in people with COVID-19, because of its high proliferation rate, low invasive nature, and the immunomodulatory, antioxidant and anti-inflammatory properties of MSCs (Samidurai and Das 2020).

*Application of ACEIs*/*ARBs*

6) Application of ACEIs/ARBs in cardiovascular diseases.

Theoretically, ACE2 levels are increased following treatment with ACE inhibitors (ACEIs) and angiotensin receptor blockers (ARBs), which yield the concerns that using these medications might increase the severity of COVID-19, especially in patients with existing cardiovascular diseases. However, the experimental and clinical data showed conflicting results. A meta-analysis revealed that continuous administration of ACEI/ARB, compared with discontinuation, significantly reduced in-hospital mortality among hypertension patients with COVID-19 infection. Meta-regression analyses indicated a clear association between the use of antihypertensive agents and reduced mortality in these patients (Liu et al. 2024). Another study also showed that antihypertensive therapy with ACEIs/ARBs might reduce the incidence of exacerbation and in-hospital mortality (Zhang et al. 2023). However, ACEI/ARB drugs may put COVID-19 patients at high risk for moderate to severe forms of COVID-19 and higher length of hospital stay. Although, it is notable that these drugs did not significantly affect specific adverse outcomes of COVID-19, such as the need for admission to the intensive care unit (ICU), length of ICU stay, ventilation, and mortality (Najafi et al. 2023). Currently, there is no evidence either from clinical or animal study showing that ACEI/ARB use increase cardiovascular complication. Therefore, it is recommended to continue the use of antihypertensive agents for patients with hypertension during SARS-CoV-2 infection (Su et al. 2020; Liu et al. 2024), especially in patients aged 80 years or older with hypertension (Zhang et al. 2023).

*Chinese traditional medicine*

7) Chinese traditional medicine: *Lianhuaqingwen* and *ShuFengJieDu* capsules (Lu 2020a)^119^ and *HuoxiangZhengqi* capsule or *HuoxiangZhengqiShui* (Jin et al. 2020).^124^ Chinese medicinal tea such as agastache leaf 6 g, perilla leaf 6 g, stewed amomum tsao-ko 6 g, dehydrated tangerine or orange peel 9 g, and ginger slice 3 slices may also be administered alone. Since the outbreak of COVID-19 in China, traditional Chinese medicine (TCM) has made an important contribution to the prevention and control of the epidemic (Kang et al. 2022). The measures taken by traditional Chinese medicine to prevent COVID-19 include: 1)Fumigation with moxa in the room, 2)Wearing perfumed Chinese herb bags (clove, fineleaf schizonepeta herb, Perilla frutescens, atractylodes lancea, cinnamon, biond magnolia flower, asarum sieboldii, and Elettaria cardamomum, 2 g for each, crushed into powder and put it into bags for external use, change a new one every 10 days). 3) Prescription of Chinese herbs for feet bath (vulgaris 10 g, carthamus 10 g, and dried ginger 6 g) Soaking the herbs in boiling water and bath the feet into the medical liquid when the temperature is suitable. Soak feet for about 20 min. 4) Prescription of Chinese herbs for prophylaxis: Astragalus mongholicus 12 g, roasted rhizoma atractylodis macrocephalae 10 g, saposhnikovia divaricata 10 g, Cyrtomium fortunei 10 g, honeysuckle 10 g, dried tangerine or orange peel 6 g, eupatorium 10 g, and licorice 10 g. Taking the medicine above yielded decoction once a day for adults, and for 5 days as a treatment course. If for children, cutting the dose to half. 5) Medical tea: perilla leaf 6 g, agastache leaf 6 g, dried tangerine or orange peel 9 g, stewed amomum tsao-ko 6 g, and 3 slices of ginger. Soak the herbs in hot water and drink the water just like enjoying the tea. 6) Chinese patent medicine: Huoxiang Zhengqi capsule or Huoxiang Zhengqi Shui (in half dose) (Jin et al. 2020). ShuFengJieDu Capsules and Lianhuaqingwen Capsule, could be the drug treatment options for 2019-nCoV (Lu 2020a). For the COVID-19 sequelae during the recovery period, Jinshuibao tablets and Shengmaiyin oral liquid significantly improved the cardiopulmonary function of recovering COVID-19 patients. Shumian capsules significantly improved patients' sleep disorders. Xiangsha Liujun pills and Ludangshen oral liquid significantly improved digestive function (An et al. 2021). A multicenter observational study showed that Chinese Medicine (CM) treatment for the post-COVID-19 patients may be a key to further promote rehabilitation and resolution of residual symptoms. Its therapeutic effects include the clinical symptom and lung function improvement, and recovery of a balanced body constitution (Zhong et al. 2022). A previous meta-analysis demonstrated that TCM could decrease the proportion of patients progressing to severe cases by 55% and the mortality rate of severe or critical patients by 49%. Moreover, TCM could relieve clinical symptoms, curtail the length of hospital stay, improve laboratory indicators, and so on. In addition, we consulted the literature and obtained 149 components of Chinese medicinal herbs that could stably bind to antiviral targets or anti-inflammatory or immune-regulating targets by the prediction of molecular docking (Kang et al. 2022). Compared with Western medicine (WM), combined Chinese herbal medicine (CHM) and WM (CHM-WM) treatment for different severity of COVID-19 showed higher total effectiveness rate, lower symptom scores of fever, cough, fatigue, dry throat and pharyngalgia, shorter mean time to viral conversion, better Computerized Tomography (CT) image and blood results, fewer total adverse events and worse conditions (P < 0.05). Subgroup analysis showed that the total effectiveness rate of combined CHM-WM group was significantly higher than WM group, especially for mild and moderate patients (Li et al. 2022). It suggested that the mechanisms involved anti-virus, anti-inflammation, and regulation of immunity (Kang et al. 2022).

*Medications*

8) Medications may also have a role in the prevention of Long COVID syndrome. In a recent randomized placebo-controlled study that included 1126 overweight and obese patients, it was shown that metformin during the acute infection reduces the incidence of Long COVID by 41.3% compared with placebo (Bramante et al. 2023). Other medications such as ivermectin and fluvoxamine were not shown to reduce the risk of Long COVID and severe acute COVID-19 infection (Bramante et al. 2022; Bramante et al. 2023).

*Potential novel therapies*

9) Potential novel therapies: the recombinant form of human ACE2 (rhACE2, APN01, potentially both neutralize the virus and protect against acute lung injury). These interventions may enhance cardiovascular health by boosting natural immunity, promoting immune cell circulation, and reducing inflammation (Saha and Sharma 2022; Chen et al. 2022; Tsampasian et al. 2024; Adu-Amankwaah 2024). Besides the mentioned interventions, early medical attention, adherence to guidelines, supportive care, mental health support, individualized treatment plans, symptom monitoring, and staying informed are vital for preventing long COVID and its associated cardiovascular complications (Adu-Amankwaah 2024). In the cardiovascular system, targeting the persistent viruses and their enzymes, maintaining RAS equilibrium, and enhancing immune responses emerge as logical approaches for addressing complications related to long COVID (Tsampasian et al. 2024; Adu-Amankwaah 2024). Recently, Aguida et al. found that near-infrared light exposure can stimulate mitochondrial metabolism to produce antioxidants, leading to an alleviation of proinflammatory signals caused by the SARS-CoV-2 infection (Aguida et al. 2023; Tangos et al. 2024). Recent studies have unveiled proprotein convertase subtilisin/kexin type 9 (PCSK9) inhibition involvement in various physiological processes, including cholesterol metabolism, inflammation, immune regulation, and thrombosis. The potential role of PCSK9 inhibition in the management of COVID-19 has emerged as an intriguing area of research. Preclinical studies suggest that PCSK9 inhibition could dampen inflammatory cascade by reducing the production of pro-inflammatory cytokines. Additionally, PCSK9 inhibition may protect against acute respiratory distress syndrome through its effects on lung injury and inflammation. PCSK9 inhibitors can lower low-density lipoprotein cholesterol (LDL-C) levels by enhancing the recycling of LDL receptors in the liver. Reduced LDL-C might protect blood vessels from further damage and lower the risk of atherosclerotic plaque formation. Moreover, PCSK9 inhibitors have potential antithrombotic effects in preclinical studies, which may mitigate the increased risk of coagulation disorders and thrombotic events in COVID-19 patients (Arsh et al. 2024).

**6 | CARDIOVASCULAR SIDE EFFECTS OF COVID-19 TREATMENT Cardiovascular side effects of COVID-19 treatment**

It is worth noting that many drugs used to for the treatment of COVID-19 have serious cardiovascular side effects and toxicity as well as both conditions that need to be cautious or avoidance of these drugs. According to previous retrospective analysis, 37.8% of patients with COVID-19 revealed adverse drug events, of which 63.8% of all events were attributed to consumption of lopinavir/ritonavir (Sun et al. 2020). For example, immune-modulating drugs hydroxychloroquine (HCQ; Plaquenil) has actions, pharmacokinetics, and metabolism similar to those of chloroquine. As an anti-malarial and anti-autoimmune agent, HCQ can inhibit the funny current channels (*I*_f_), delay rectifier potassium currents (*I*_Kr_) and L-type calcium ion currents (*I*_CaL_). HCQ blocks these channels and causes QT prolongation. Therefore, HCQ should be avoided with concomitant QT-prolonging medications such as azithromycin, metabolic derangements, and renal failure (Yu et al. 2021). No clinically significant effect on QT prolongation was observed in healthy subjects at therapeutic doses of tocilizumab. Some patients taking tocilizumab experienced rapid increases in blood pressure and low-density lipoprotein cholesterol (Yu et al. 2021). No clinically significant effects of interferon-β on QTc prolongation have been observed. Only rare case reports showed premature ventricular captures and atrioventricular block (Yu et al. 2021). Fingolimod (FTY-720; an oral immunomodulating agent; antagonist of lipid sphingosine-1-phosphate 1 receptors in the lymph node T cells). Fingolimod has been reported to induce bradyarrhythmia, atrioventricular block, and blood pressure through increasing the vascular tone. It may cause retinal arterial vasospasm and retinal vein occlusion (Yu et al. 2021)

many of the antivirals, antibiotics, antimalarials (chloroquine or hydroxychloroquine), and azithromycin used to treat COVID-19 are known to prolong the QT interval and increase the risk of torsades de pointes (TdP), and antimalarials such as chloroquine or hydroxychloroquine, and azithromycin may lead to QT interval prolongation (Gautret et al. 2020; Giudicessi et al. 2020; Alblaihed et al. 2023),^125-127^ thrombocytopenia and anemia. In COVID-19 patients with abnormal cardiac structure or function (such as left ventricular hypertrophy or reduced ejection fraction), QTc prolongation may increase the risk of torsade de pointes ventricular tachycardia. Fortunately, the Heart Rhythm Society has recently released the management guidelines for QTc prolongation in COVID-19 drug treatment and other cardiac electrophysiology issues related to COVID-19 (Lakkireddy et al. 2020).^128^ Antiviral drugs including ribavarin, lopinivir or ritonivir may induce thrombocytopenia, haemolytic anemia, hyperlipidemia and hypertriglyceridemia.  Although extensive cardiovascular toxicities and drug interactions have not yet been reported, prior evaluations of remdesivir during the Ebola outbreak noted that one patient developed hypotension and subsequent cardiac arrest (Lucey 2019; Yu et al. 2021). Dose-related asymptomatic prolongation in the PR interval with atazanavir has been observed in clinical studies (Yu et al. 2021). Ritonavir/lopinavir has been shown to cause QT and PR interval prolongation in some healthy adults, and second- or third-degree atrioventricular block in patients with underlying structural heart disease and pre-existing conduction system abnormalities (Yu et al. 2021). The renal adverse effects of monotherapy of lopinavir/ritonavir in COVID-19 patients, or combining antiviral agents metabolized by CYP3A4 with HCQ are significantly associated with a higher incidence of acute kidney injury such as a lower GFR as well as glycosuria and proteinuria (Schneider et al. 2021; Jahanshahi et al. 2024 ). The QT interval prolongation risk of favipiravir is considered to be low (Yu et al. 2021). No effect on the QT interval was observed in patients receiving ribavirin (Yu et al. 2021). Tachycardia, orthostatic hypotension, and PR interval prolongation have been documented in case reports of ivermectin treatment (Yu et al. 2021). Remedsivir is considered to have adverse effects on glomerular filtration rate (GFR) (Jahanshahi et al. 2024).

Biological products i.e. tocilizumab and interferon-α 2B may occur hypertension, thrombocytopenia, anaemia, elevated liver transaminases, and hyperlipidemia or hypertriglyceridemia (Su et al. 2022).^67^ These data of side effects and toxicities come from patients that use these drugs chronically for the treatment of autoimmune diseases (chloroquine/hydroxychloroquine, rocilizumab), hepatitis (ribavarin, IFN-a), or HIV infection lopinivir/ritonivir). Therefore, the impact of short-term use of these drugs for COVID-19 patients is still unknown. The cardiovascular side effects and toxicity of redcivir are not yet clear (Nakkazi 2018).^129^ Recently, both chloroquine and hydroxychloroquine have been used to treat and prevent COVID-19. However, there were few data supporting the effectiveness of these drugs so far, and the cardiovascular side effects and toxicity are also considerable (Parvu et al. 2022; Chimenti et al. 2022). In a previous systematic review, Chatre et al. (2018) ^130^ showed that the main side effects of these medicines were cardiac conduction disorder (85%). Moreover, these drugs had a long duration (median: 7 years) and a high cumulative dose. Other disadvantageous cardiovascular events included heart failure (26.8%), ventricular hypertrophy (22%), dyskinesia (9.4%), valve dysfunction (7.1%), and pulmonary hypertension (3.9%). A large number of patients (44.9%) could return to normal cardiac function after discontinuing chloroquine and hydroxychloroquine, while other patients continued to experience irreversible damage (12.9%) or die from drug side effects (30.8%) (Liang et al. 2020).^123^ Therefore, these drugs should be carefully used, especially in the absence of more powerful data on their efficacy. It is worth noting that tolizumab could affect lipid metabolism in patients with cardiovascular disease. It obviously increased total cholesterol, low-density lipoprotein and high-density lipoprotein cholesterol, while significantly decreased high-density lipoprotein serum amyloid A (HDL-SAA), secretory phospholipase A2 IIA and lipoprotein (a) (Gabay et al. 2016).^131^ Very recently, ENTRACE clinical trial supports the cardiovascular safety of tolimab in patients with rheumatoid arthritis (Giles et al. 2020).^132^ However, up to now, IL-6 targeting has not been applied to the secondary prevention of cardiovascular diseases.

**7 | CONCLUSION Conclusions**

To sum up, the cardiovascular damage in of COVID-19 patients is common and portends different prognosis across different populations. Therefore, differentiating between the various causes of cardiovascular damage in different populations is crucial to determining the treatment course. Worse clinical outcomes were found in COVID-19 patients with cardiovascular damage or preexisting cardiovascular disease risk factors or cardiac disease; in Asian, Black and Latinx populations; in men, and in elderly peoples. These damages mainly manifested as myocarditis, hypertension, arrhythmia, heart failure, coronary heart disease and thrombosis events. The occurrence of these complications often aggravates the disease and increases the mortality. Therefore, early recognition of these abnormalities among hospitalized COVID-19 patients is critical measures to identify patients with poor prognosis, guide treatment, and improve patients’ clinical outcomes. However, the long-term effects and their pathogenic mechanisms of SARS-CoV-2 on the cardiovascular system are not well-known (Tsampasian et al. 2024; Adu-Amankwaah 2024). The mechanisms sustaining the lingering effects of post-acute COVID-19 in the cardiovascular system may be associated with the variations in individuals’ genetic predispositions and changes in immune responses to SARS-CoV-2. The impact of renin–angiotensin system (RAS) on long COVID remains elusive (Tsampasian et al. 2024; Adu-Amankwaah 2024). This enigmatic phenomenon, was termed “long COVID” or “post-COVID-19 condition” by the World Health Organization (WHO, Tsampasian et al. 2024; Adu-Amankwaah 2024), and was defined as the “continuation or development of new symptoms 3 months after the initial SARS-CoV-2 infection, with the symptoms lasting for at least 2 months with no other explanation” (Soriano et al. 2022; Tsampasian et al. 2024; Adu-Amankwaah 2024). Nearly 45% of patients who have survived COVID-19 are contending with persistent symptoms even 4 months following the initial infection (O'Mahoney et al. 2022; Tsampasian et al. 2024; Adu-Amankwaah 2024). A 12- year follow-up study of 25 patients who recuperated from SARS-CoV infection revealed that they developed hyperlipidaemia (68%), CVS disorders (44%), and glucose metabolism abnormalities (60%) (Wu et al. 2017; Zheng et al 2020; Adu-Amankwaah et al. 2021). Therefore, we still need to conduct long-term follow-up and a lot of research and exploration.

**Abbreviations**

2019 nCoV: 2019 novel coronavirus; ACE2: Angiotensin converting enzyme 2; ACEI: Angiotensin-converting enzyme inhibitor ARB: Angiotensin receptor blocker; ARDS: Acute respiratory distress syndrome; AT2 cells: Type II alveolar epithelial cells; CDC: Center for Disease Control; CDCs: Cardiosphere-derived cells; CK: Creatine kinase; CK-MB: Creatine kinase MB; CoV: Coronavirus; COVID-19: Coronavirus disease 2019; CTD: C-terminal domain; cTnI: Cardiac troponin I; DIC: Diffuse intravascular coagulation; ECMO: Extracorporeal membrane oxygenation; EMB: Endocardial myocardial biopsy; HDL-SAA: High-density lipoprotein serum amyloid A; HR: Heptapeptide repeat; hs-cTn: High-sensitivity cardiac troponin; hs-cTnI: High-sensitivity cardiac troponin I; IABP: Intraaortic balloon pump; ICU: Intensive care unit; IFN: Interferon; IL: Interleukin; LDH: Lactate dehydrogenase; MERS: Middle East respiratory syndrome; MHC: Major histocompatibility complex; MRI: Magnetic resonance imaging; MSCs: Mesenchymal stem cells; NCP: Novel coronavirus pneumonia; NT proBNP: N-terminal pro B-type natriuretic peptide; NTD: N-terminal domain; OR: Odds ratio; ORF: Open reading frame; RAAS: Renin-angiotensin-aldosterone system; RBD: Receptor binding domain; RdRp: RNA dependent RNA polymerase; RT-PCR: Reverse transcription-polymerase chain reaction; SARS: Severe acute respiratory syndrome; SARS-CoV-2: Severe acute respiratory syndrome coronavirus 2; Th: T helper cells; TMPRSS2: Transmembrane serine protease 2 (TMPRSS2); TNF: Tumor necrosis factor; WHO: World Health Organization.

**Acknowledgements**

Not applicable.

**ORCID**

Rui-Xing Yin: https://orcid.org/0000-0001-7883-4310

**Author contributions**

H.-H.S. performed the literature search and review, wrote the manuscript, and ideated and produced the figures. R.-X.Y. conceived and designed the paper, ideated and produced the figures, and critically revised the manuscript. Both authors read and approved the final manuscript.

**Funding**

This study was supported by the Nanning Science and Technology Major Project (No: 20193008). The funder had no role in the design of the study; in the collection, analyses, or interpretation of data; in the writing of the manuscript, or in the decision to publish the results.

**Availability of data and materials**

Not applicable.

**Declarations**

**Ethics approval and consent to participate**

This article does not contain any studies with human participants or animals performed by both of the authors. Not applicable.

**Consent for publication**

Both authors gave consent for publication. Not applicable.

**Competing interests**

The authors declare that they have no competing interests.

**Author details**

^1^ Department of Infectious Diseases, HIV/AIDS Clinical Treatment Center of Guangxi (Nanning), The Fourth People’s Hospital of Nanning, No. 1 Erli, Changgang Road, Nanning 530023, Guangxi, People’s Republic of China. ^2^ Department of Cardiology, Institute of Cardiovascular Diseases, The First Affiliated Hospital, Guangxi Medical University, 6 Shuangyong Road, Nanning 530021, Guangxi, People’s Republic of China.

**References**

1. Li Q, Guan X, Wu P, et al. Early transmission dynamics in Wuhan, China, of novel coronavirus–infected pneumonia. *N Engl J Med*. 2020;382(13):1199–1207.
2. Wang D, Hu B, Hu C, et al. Clinical characteristics of 138 hospitalized patients with 2019 novel coronavirus–infected pneumonia in Wuhan, China. *JAMA*. 2020;323(11):1061–1069.
3. Firouzabadi N, Ghasemiyeh P, Moradishooli F, Mohammadi-Samani S. Update on the effectiveness of COVID-19 vaccines on different variants of SARS-CoV-2. *Int Immunopharmacol*. 2023;117:109968.
4. Tomasoni D, Italia L, Adamo M, et al. COVID 19 and heart failure: from infection to inflammation and angiotensin II stimulation. Searching for evidence from a new disease. *Eur J Heart Fail*. 2020;22(6):957–966.
5. Shi S, Qin M, Shen B, et al. Association of cardiac injury with mortality in hospitalized patients with COVID-19 in Wuhan, China. *JAMA Cardiol*. 2020;5(7):802–810.
6. Guo T, Fan Y, Chen M, et al. Cardiovascular implications of fatal outcomes of patients with coronavirus disease 2019 (COVID-19). *JAMA Cardiol*. 2020;5(7):811–818.
7. Bonow RO, Fonarow GC, O’Gara PT, Yancy CW. Association of coronavirus disease 2019 (COVID-19) with myocardial injury and mortality. *JAMA Cardiol*. 2020;5(7):751–753.
8. Zheng YY, Ma YT, Zhang JY, Xie X. COVID-19 and the cardiovascular system. *Nat Rev Cardiol*. 2020;17(5):259–260.
9. Ruan Q, Yang K, Wang W, Jiang L, Song J. Clinical predictors of mortality due to COVID-19 based on an analysis of data of 150 patients from Wuhan, China. *Intensive Care Med*. 2020;46(5):846–848.
10. Santoso A, Pranata R, Wibowo A, Al-Farabi MJ, Huang I, Antariksa B. Cardiac injury is associated with mortality and critically ill pneumonia in COVID-19: A meta-analysis. *Am J Emerg Med*. 2021;44:352–357.
11. Tian W, Jiang W, Yao J, et al. Predictors of mortality in hospitalized COVID-19 patients: A systematic review and meta-analysis. *J Med Virol*. 2020;92(10):1875–1883.
12. Guzik TJ, Mohiddin SA, Dimarco A, et al. COVID-19 and the cardiovascular system: implications for risk assessment, diagnosis, and treatment options. *Cardiovasc Res*. 2020;116(10):1666–1687.
13. Huang C, Wang Y, Li X, et al. Clinical features of patients infected with 2019 novel coronavirus in Wuhan, China. *Lancet*. 2020;395(10223):497–506.
14. Schoeman D, Fielding BC. Coronavirus envelope protein: current knowledge. *Virol J*. 2019;16(1):69.
15. Hoffmann M, Kleine-Weber H, Schroeder S, et al. SARS-CoV-2 cell entry depends on ACE2 and TMPRSS2 and is blocked by a clinically proven protease inhibitor. *Cell*. 2020;181(2):271–280.e8.
16. Lim J, Jeon S, Shin HY, et al. Case of the Index Patient Who Caused Tertiary Transmission of COVID-19 Infection in Korea: the Application of Lopinavir/Ritonavir for the Treatment of COVID-19 Infected Pneumonia Monitored by Quantitative RT-PCR. *J Korean Med Sci*. 2020;35(6):e79.
17. Turner AJ, Hiscox JA, Hooper NM. ACE2: from vasopeptidase to SARS virus receptor. *Trends Pharmacol Sci*. 2004;25(6):291–294.
18. Hooper NM, Lambert DW, Turner AJ. Discovery and characterization of ACE2 - a 20-year journey of surprises from vasopeptidase to COVID-19. *Clin Sci* (*Lond*). 2020;134(18):2489–2501.
19. Xu X, Chen P, Wang J, et al. Evolution of the novel coronavirus from the ongoing Wuhan outbreak and modeling of its spike protein for risk of human transmission. *Sci China Life Sci*. 2020;63(3):457–460.
20. Zhang H, Kang Z, Gong H, et al. Digestive system is a potential route of COVID-19: an analysis of single-cell coexpression pattern of key proteins in viral entry process. *Gut*. 2020;69(6): 1010–1018.
21. Wan Y, Shang J, Graham R, Baric RS, Li F. Receptor recognition by novel coronavirus from Wuhan: An analysis based on decade-long structural studies of SARS. *J Virol*. 2020;94(7):e00127-20.
22. Walls AC, Park YJ, Tortorici MA, Wall A, McGuire AT, Veesler D. Structure, function, and antigenicity of the SARS-CoV-2 spike glycoprotein. *Cell*. 2020;181(2):281–292.e6.
23. Ren LL, Wang YM, Wu ZQ, et al. Identification of a novel coronavirus causing severe pneumonia in human: A descriptive study. *Chin Med J* (*Engl*). 2020;133(9):1015–1024.
24. Chan JF, Kok KH, Zhu Z, et al. Genomic characterization of the 2019 novel human-pathogenic coronavirus isolated from a patient with atypical pneumonia after visiting Wuhan. *Emerg Microbes Infect*. 2020;9(1):221–236.
25. Liu CX, Wang YL, Yan FY. Understanding novel coronavirus pneumonia, paying attention to research and development of epidemic prevention and control drugs. *Chinese Journal of Antibiotics*. 2020;45(2):1–10.
26. Ceraolo C, Giorgi FM. Genomic variance of the 2019-nCoV coronavirus. *J Med Virol*. 2020;92(5):522–528.
27. Benvenuto D, Giovanetti M, Ciccozzi A, Spoto S, Angeletti S, Ciccozzi M. The 2019-new coronavirus epidemic: Evidence for virus evolution. *J Med Virol*. 2020;92(4):455–459.
28. Lake MA. What we know so far: COVID-19 current clinical knowledge and research. *Clin Med* (*Lond*). 2020;20(2):124–127.
29. Andersen KG, Rambaut A, Lipkin WI, Holmes EC, Garry RF. The proximal origin of SARS-CoV-2. *Nat Med*. 2020;26(4):450–452.
30. Lu R, Zhao X, Li J, et al. Genomic characterisation and epidemiology of 2019 novel coronavirus: implications for virus origins and receptor binding. *Lancet*. 2020;395(10224):565–574.
31. Lian J, Jin X, Hao S, et al. Analysis of epidemiological and clinical features in older patients with coronavirus disease 2019 (covid-19) outside Wuhan. *Clin Infect Dis*. 2020; 71(15):740–747.
32. Chan JF, Yuan S, Kok KH, et al. A familial cluster of pneumonia associated with the 2019 novel coronavirus indicating person-to-person transmission: a study of a family cluster. *Lancet*. 2020;395(10223):514–523.
33. Chen N, Zhou M, Dong X, et al. Epidemiological and clinical characteristics of 99 cases of 2019 novel coronavirus pneumonia in Wuhan, China: a descriptive study. *Lancet*. 2020;395(10223):507–513.
34. Jin YH, Cai L, Cheng ZS, et al. A rapid advice guideline for the diagnosis and treatment of 2019 novel coronavirus (2019-nCoV) infected pneumonia (standard version). *Mil Med Res*. 2020;7(1):4.
35. Rothe C, Schunk M, Sothmann P, et al. Transmission of 2019-nCoV infection from an asymptomatic contact in Germany. *N Engl J Med*. 2020;382(10):970–971.
36. Callaway E, Cyranoski D, Mallapaty S, Stoye E, Tollefson J. The coronavirus pandemic in five powerful charts. *Nature*. 2020;579(7800):482–483.
37. Zhao S, Lin Q, Ran J, et al. Preliminary estimation of the basic reproduction number of novel coronavirus (2019-nCoV) in China, from 2019 to 2020: A data-driven analysis in the early phase of the outbreak.*Int J Infect Dis*. 2020;92:214–217.
38. Jiang S, Xia S, Ying T, Lu L. A novel coronavirus (2019-nCoV) causing pneumonia-associated respiratory syndrome. *Cell Mol Immunol*. 2020;17(5):554.
39. Holshue ML, DeBolt C, Lindquist S, et al. First case of 2019 novel coronavirus in the United States. *N Engl J Med*. 2020;382(10):929–936.
40. Shen KL, Yang YH. Diagnosis and treatment of 2019 novel coronavirus infection in children: a pressing issue. *World J Pediatr*. 2020;16(3):219–221.
41. Wu JT, Leung K, Bushman M, et al. Estimating clinical severity of COVID-19 from the transmission dynamics in Wuhan, China. *Nat Med*. 2020;26(4):506–510.
42. Zhou F, Yu T, Du R, et al. Clinical course and risk factors for mortality of adult inpatients with COVID-19 in Wuhan, China: a retrospective cohort study. *Lancet*. 2020;395(10229):1054–1062.
43. Tang N, Li D, Wang X, Sun Z. Abnormal coagulation parameters are associated with poor prognosis in patients with novel coronavirus pneumonia. *J Thromb Haemost*. 2020;18(4):844–847.
44. Ho JS, Sia CH, Chan MY, Lin W, Wong RC. Coronavirus-induced myocarditis: A meta-summary of cases. *Heart Lung*. 2020;49(6):681–685.
45. Siripanthong B, Nazarian S, Muser D, et al. Recognizing COVID-19-related myocarditis: The possible pathophysiology and proposed guideline for diagnosis and management. *Heart Rhythm*. 2020;17(9):1463–1471.
46. Patone M, Mei XW, Handunnetthi L, et al. Risks of myocarditis, pericarditis, and cardiac arrhythmias associated with COVID-19 vaccination or SARS-CoV-2 infection. *Nat Med*. 2022;28(2):410–422.
47. Castiello T, Georgiopoulos G, Finocchiaro G, et al. COVID-19 and myocarditis: a systematic review and overview of current challenges. *Heart Fail Rev*. 2022;27(1):251–261.
48. Sawalha K, Abozenah M, Kadado AJ, et al. Systematic review of COVID-19 related myocarditis: insights on management and outcome. *Cardiovasc Revasc Med*. 2021;23:107–113.
49. Liu J, Deswal A, Khalid U. COVID-19 myocarditis and long-term heart failure sequelae. *Curr Opin Cardiol*. 2021;36(2):234–240.
50. Daniels CJ, Rajpal S, Greenshields JT, et al. Prevalence of Clinical and Subclinical Myocarditis in Competitive Athletes With Recent SARS-CoV-2 Infection: Results From the Big Ten COVID-19 Cardiac Registry. *JAMA Cardiol*. 2021;6(9):1078–1087.
51. Caforio ALP, Baritussio A, Basso C, Marcolongo R. Clinically suspected and biopsy-proven myocarditis temporally associated with SARS-CoV-2 infection. *Annu Rev Med*. 2022;73:149–166.
52. Gnecchi M, Moretti F, Bassi EM, et al. Myocarditis in a 16-year-old boy positive for SARS-CoV-2. *Lancet*. 2020;395(10242):e116.
53. Yokoo P, Fonseca EKUN, Sasdelli Neto R, et al. COVID-19 myocarditis: a case report. *Einstein* (*Sao Paulo*). 2020;18:eRC5876.
54. Chiu JS, Lahoud-Rahme M, Schaffer D, Cohen A, Samuels-Kalow M. Kawasaki disease features and myocarditis in a patient with COVID-19. *Pediatr Cardiol*. 2020;41(7):1526–1528.
55. Warchoł I, Dębska-Kozłowska A, Karcz-Socha I, Książczyk M, Szymańska K, Lubiński A. Terra incognita: clinically suspected myocarditis in a patient with severe acute respiratory syndrome coronavirus 2 infection. *Pol Arch Intern Med*. 2020;130(5):446–448.
56. Chen C, Zhou Y, Wang DW. SARS-CoV-2: a potential novel etiology of fulminant myocarditis. *Herz*. 2020;45(3):230–232.
57. Hu H, Ma F, Wei X, Fang Y. Coronavirus fulminant myocarditis saved with glucocorticoid and human immunoglobulin. *Eur Heart J*. 2021;42(2):206.
58. Zeng JH, Liu YX, Yuan J, et al. First case of COVID- 19 complicated with fulminant myocarditis: a case report and insights. *Infection*. 2020;48(5):773–777.
59. Li C, Chen Y, Zhao Y, et al. Intravenous injection of coronavirus disease 2019 (COVID-19) mRNA vaccine can induce acute myopericarditis in mouse model. *Clin Infect Dis*. 2022;74(11):1933–1950.
60. Yao XH, Li TY, He ZC, et al. A pathological report of three COVID-19 cases by minimally invasive autopsies. *Zhonghua Bing Li Xue Za Zhi*. 2020;49(5):411–417.
61. Blyszczuk P. Myocarditis in humans and in experimental animal models. *Front Cardiovasc Med*. 2019;6:64.
62. Tajbakhsh A, Gheibi Hayat SM, Taghizadeh H, et al. COVID-19 and cardiac injury: clinical manifestations, biomarkers, mechanisms, diagnosis, treatment, and follow up. *Expert Rev Anti Infect Ther*. 2021;19(3):345–357.
63. Guan WJ, Ni ZY, Hu Y, et al. Clinical Characteristics of Coronavirus Disease 2019 in China. *N Engl J Med*. 2020;382(18):1708–1720.
64. Beaney T, Burrell LM, Castillo RR, et al. May Measurement Month 2018: a pragmatic global screening campaign to raise awareness of blood pressure by the International Society of Hypertension. *Eur Heart J*. 2019;40(25):2006–2017.
65. Epidemiology Working Group for NCIP Epidemic Response, Chinese Center for Disease Control and Prevention. Epidemiology Working Group for NCIP Epidemic Response, Chinese Center for Disease Control and Prevention. The epidemiological characteristics of an outbreak of 2019 novel coronavirus diseases (COVID-19) in China. *Zhonghua Liu Xing Bing Xue Za Zhi*. 2020;41(2):145–151.
66. Kreutz R, Algharably E, Azizi M, et al. Hypertension, the renin–angiotensin system, and the risk of lower respiratory tract infections and lung injury: implications for COVID-19. European Society of Hypertension COVID-19 Task Force Review of Evidence. *Cardiovasc Res*. 2020;116(10):1688–1699.
67. Su S, Chen R, Zhang S, Shu H, Luo J. Immune system changes in those with hypertension when infected with SARS-CoV-2. *Cell Immunol*. 2022;378:104562.
68. Shukla AK, Banerjee M. Angiotensin-converting-enzyme 2 and renin-angiotensin system inhibitors in COVID-19: an update. *High Blood Press Cardiovasc Prev*. 2021;28(2):129–139.
69. Vaduganathan M, Vardeny O, Michel T, McMurray JJV, Pfeffer MA, Solomon SD. Renin–angiotensin–aldosterone system inhibitors in patients with Covid-19.*N Engl J Med*. 2020;382(17):1653–1659.
70. Danser AHJ, Epstein M, Batlle D. Renin-angiotensin system blockers and the COVID-19 pandemic: at present there is no evidence to abandon renin-angiotensin system blockers. *Hypertension*. 2020;75(6):1382–1385.
71. Sommerstein R, Kochen MM, Messerli FH, Gräni C. Coronavirus Disease 2019 (COVID- 19): Do Angiotensin-Converting Enzyme Inhibitors/Angiotensin Receptor Blockers Have a Biphasic Effect? *J Am Heart Assoc*. 2020;9(7):e016509.
72. Guo J, Huang Z, Lin L, Lv J. Coronavirus Disease 2019 (COVID-19) and Cardiovascular Disease: A Viewpoint on the Potential Influence of Angiotensin- Converting Enzyme Inhibitors/Angiotensin Receptor Blockers on Onset and Severity of Severe Acute Respiratory Syndrome Coronavirus 2 Infection. *J Am Heart Assoc*. 2020;9(7):e016219.
73. Drummond GR, Vinh A, Guzik TJ, Sobey CG. Immune mechanisms of hypertension. *Nat Rev Immunol*. 2019;19(8):517–532.
74. Loperena R, Van Beusecum JP, Itani HA, et al. Hypertension and increased endothelial mechanical stretch promote monocyte differentiation and activation: roles of STAT3, interleukin 6 and hydrogen peroxide.*Cardiovasc Res*. 2018;114(11):1547–1563.
75. Siedlinski M, Jozefczuk E, Xu X, et al. White blood cells and blood pressure: a Mendelian randomization study. *Circulation*. 2020;141(16):1307–1317.
76. Youn JC, Yu HT, Lim BJ, et al. Immunosenescent CD8+ T cells and C-X-C chemokine receptor type 3 chemokines are increased in human hypertension. *Hypertension*. 2013;62(1):126–133.
77. Liu K, Fang YY, Deng Y, et al. Clinical characteristics of novel coronavirus cases in tertiary hospitals in Hubei Province. *Chin Med J* (*Engl*). 2020;133(9):1025–1031.
78. Varney JA, Dong VS, Tsao T, et al. COVID-19 and arrhythmia: An overview. *J Cardiol*. 2022;79(4):468–475.
79. Kochav SM, Coromilas E, Nalbandian A, et al. Cardiac arrhythmias in COVID-19 infection. *Circ Arrhythm Electrophysiol*. 2020;13(6):e008719.
80. Bhatla A, Mayer MM, Adusumalli S, et al. COVID-19 and cardiac arrhythmias. *Heart Rhythm*. 2020;17(9):1439–1444.
81. Pimentel M, Magalhães APA, Novak CV, May BM, Rosa LGBD, Zimerman LI. Cardiac Arrhythmias in Patients with COVID-19. *Arq Bras Cardiol*. 2021;117(5):1010–1015.
82. Manolis AS, Manolis AA, Manolis TA, Apostolopoulos EJ, Papatheou D, Melita H. COVID-19 infection and cardiac arrhythmias. *Trends Cardiovasc Med*. 2020;30(8):451–460.
83. Kochi AN, Tagliari AP, Forleo GB, Fassini GM, Tondo C. Cardiac and arrhythmic complications in patients with COVID-19. *J Cardiovasc Electrophysiol*. 2020;31(5):1003–1008.
84. Dherange P, Lang J, Qian P, et al. Arrhythmias and COVID-19: A Review.*JACC Clin Electrophysiol*. 2020;6(9):1193–1204.
85. Lippi G, Lavie CJ, Sanchis-Gomar F. Cardiac troponin I in patients with coronavirus disease 2019 (COVID-19): evidence from a meta-analysis.*Prog Cardiovasc Dis*. 2020;63(3):390–391.
86. Chen L, Li X, Chen M, Feng Y, Xiong C. The ACE2 expression in human heart indicates new potential mechanism of heart injury among patients infected with SARS-CoV-2. *Cardiovasc Res*. 2020;116(6):1097–1100.
87. Babapoor-Farrokhran S, Gill D, Walker J, Rasekhi RT, Bozorgnia B, Amanullah A. Myocardial injury and COVID-19: Possible mechanisms. *Life Sci*. 2020;253:117723.
88. Xu Z, Shi L, Wang Y, et al. Pathological findings of COVID-19 associated with acute respiratory distress syndrome. *Lancet Respir Med*. 2020;8(4):420–422.
89. Varga Z, Flammer AJ, Steiger P, et al. Endothelial cell infection and endotheliitis in COVID-19. *Lancet*. 2020;395(10234):1417–1418.
90. Klok FA, Kruip MJHA, van der Meer NJM, et al. Incidence of thrombotic complications in critically ill ICU patients with COVID-19. *Thromb Res*. 2020;191:145–147.
91. Kang Y, Chen T, Mui D, et al. Cardiovascular manifestations and treatment considerations in covid-19. *Heart*. 2020;106(15):1132–1141.
92. Peiris JS, Chu CM, Cheng VC, et al. Clinical progression and viral load in a community outbreak of coronavirus- associated SARS pneumonia: a prospective study. *Lancet*. 2003;361(9371):1767–1772.
93. Liang C, Zhang W, Li S, Qin G. Coronary heart disease and COVID-19: A meta-analysis. *Med Clin* (*Barc*). 2021;156(11):547–554.
94. Musher DM, Abers MS, Corrales-Medina VF. Acute infection and myocardial infarction. *N Engl J Med*. 2019;380(2):171–176.
95. Cole JE, Park I, Ahern DJ, et al. Immune cell census in murine atherosclerosis: cytometry by time of flight illuminates vascular myeloid cell diversity. *Cardiovasc Res*. 2018;114(10):1360–1371.
96. Warren-Gash C, Hayward AC, Hemingway H, et al. Influenza infection and risk of acute myocardial infarction in England and Wales: a CALIBER self-controlled case series study. *J Infect Dis*. 2012;206(11):1652–1659.
97. Sugiura M, Hiraoka K, Ohkawa S, Ueda K, Matsuda T. A clinicopathological study on cardiac lesions in 64 cases of disseminated intravascular coagulation. *Jpn Heart J*. 1977;18(1):57–69.
98. Wang YD, Zhang SP, Wei QZ, et al. COVID-19 complicated with DIC: 2 cases report and literatures review. *Zhonghua Xue Ye Xue Za Zhi*. 2020;41(3):245–247.
99. Fried JA, Ramasubbu K, Bhatt R, et al. The variety of cardiovascular presentations of COVID-19. *Circulation*. 2020;141(23):1930–1936.
100. Sala S, Peretto G, Gramegna M, et al. Acute myocarditis presenting as a reverse Tako-Tsubo syndrome in a patient with SARS-CoV-2 respiratory infection.*Eur Heart J*. 2020;41(19):1861–1862.
101. Arentz M, Yim E, Klaff L, et al. Characteristics and outcomes of 21 critically ill patients with COVID-19 in Washington state. *JAMA*. 2020;323(16):1612–1614.
102. Inciardi RM, Lupi L, Zaccone G, et al. Cardiac involvement in a patient with coronavirus disease 2019 (COVID-19). *JAMA Cardiol*. 2020;5(7):819–824.
103. Mehta P, McAuley DF, Brown M, et al. COVID-19: consider cytokine storm syndromes and immunosuppression.*Lancet*. 2020;395(10229):1033–1034.
104. Mann DL. Innate immunity and the failing heart: the cytokine hypothesis revisited. *Circ Res*. 2015;116(7):1254–1268.
105. Ali MAM, Spinler SA. COVID-19 and thrombosis: From bench to bedside. *Trends Cardiovasc Med*. 2021;31(3):143–160.
106. Danzi GB, Loffi M, Galeazzi G, Gherbesi E. Acute pulmonary embolism and COVID-19 pneumonia: a random association? *Eur Heart J*. 2020;41(19):1858.
107. Wichmann D, Sperhake JP, Lütgehetmann M, et al. Autopsy findings and venous thromboembolism in patients with COVID-19: A prospective cohort study. *Ann Intern Med*. 2020;173(4):268–277.
108. Wichmann D. Autopsy findings and venous thromboembolism in patients with COVID-19. *Ann Intern Med*. 2020;173(12):1030.
109. Gąsecka A, Borovac JA, Guerreiro RA, et al. Thrombotic complications in patients with COVID-19: pathophysiological mechanisms, diagnosis, and treatment. *Cardiovasc Drugs Ther*. 2021;35(2):215–229.
110. Kyriakoulis KG, Kokkinidis DG, Kyprianou IA, et al. Venous thromboembolism in the era of COVID-19. *Phlebology*. 2021;36(2):91–99.
111. Heinrich F, Roedl K, Jarczak D, et al. New insights in the occurrence of venous thromboembolism in critically ill patients with COVID-19-a large postmortem and clinical analysis. *Viruses*. 2022;14(4):811.
112. Marvi TK, Stubblefield WB, Tillman BF, et al. Serial thromboelastography and the development of venous thromboembolism in critically ill patients with COVID-19. *Crit Care Explor*. 2022;4(1):e0618.
113. Tsaplin S, Schastlivtsev I, Zhuravlev S, Barinov V, Lobastov K, Caprini JA. The original and modified Caprini score equally predicts venous thromboembolism in COVID-19 patients. *J Vasc Surg Venous Lymphat Disord*. 2021;9(6):1371–1381.e4.
114. Farkouh ME, Stone GW, Lala A, et al. Anticoagulation in patients with COVID-19: JACC Review Topic of the Week. *J Am Coll Cardiol*. 2022;79(9):917–928.
115. Epelbaum O. Autopsy findings and venous thromboembolism in patients with COVID-19. *Ann Intern Med*. 2020;173(12):1029–1030.
116. Takasu S, Ariizumi M, Matsumoto S, Nakagawa H, Iwadate K. Cerebral venous sinus thrombosis associated with COVID-19: an autopsy case report. *Forensic Sci Med Pathol*. 2022;18(1):80–85.
117. Hendren NS, Drazner MH, Bozkurt B, Cooper LT Jr. Description and proposed management of the acute COVID-19 cardiovascular syndrome. *Circulation*. 2020;141(23):1903–1914.
118. Shi Z, Fu W. Diagnosis and treatment recommendation for novel coronavirus pneumonia related isolated distal deep vein thrombosis. *Shanghai Medical Journal*. 2020;43(4):207-210.
119. Lu H. Drug treatment options for the 2019-new coronavirus (2019-nCoV). *Biosci Trends*. 2020;14(1):69–71.
120. Morse JS, Lalonde T, Xu S, Liu WR. Learning from the past: possible urgent prevention and treatment options for severe acute respiratory infections caused by 2019-nCoV. *Chembiochem*. 2020;21(5):730–738.
121. Marbán E. A mechanistic roadmap for the clinical application of cardiac cell therapies. *Nat Biomed Eng*. 2018;2(6):353–361.
122. Leng Z, Zhu R, Hou W, et al. Transplantation of ACE2- mesenchymal stem cells improves the outcome of patients with COVID-19 pneumonia. *Aging Dis*. 2020;11(2):216–228.
123. Liang B, Chen J, Li T, et al. Clinical remission of a critically ill COVID-19 patient treated by human umbilical cord mesenchymal stem cells: A case report. *Medicine* (*Baltimore*). 2020 Jul 31;99(31):e21429.
124. Jin YH, Cai L, Cheng ZS, et al. A rapid advice guideline for the diagnosis and treatment of 2019 novel coronavirus (2019-nCoV) infected pneumonia (standard version). *Mil Med Res*. 2020;7(1):4. (see 34)
125. Gautret P, Lagier JC, Parola P, et al. Hydroxychloroquine and azithromycin as a treatment of COVID-19: results of an open- label non- randomized clinical trial. *Int J Antimicrob Agents*. 2020;56(1):105949.
126. White NJ. Cardiotoxicity of antimalarial drugs. *Lancet Infect Dis*. 2007;7(8):549–558.
127. Giudicessi JR, Noseworthy PA, Friedman PA, Ackerman MJ. Urgent Guidance for Navigating and Circumventing the QTc-Prolonging and Torsadogenic Potential of Possible Pharmacotherapies for Coronavirus Disease 19 (COVID-19). *Mayo Clin Proc*. 2020 Jun;95(6):1213–1221.
128. Lakkireddy DR, Chung MK, Gopinathannair R, et al. Guidance for cardiac electrophysiology during the COVID-19 pandemic from the Heart Rhythm Society COVID-19 Task Force; Electrophysiology Section of the American College of Cardiology; and the Electrocardiography and Arrhythmias Committee of the Council on Clinical Cardiology, American Heart Association. *Heart Rhythm*. 2020;17(9):e233–e241.
129. Nakkazi E. Randomised controlled trial begins for Ebola therapeutics. *Lancet*. 2018;392(10162):2338.
130. Chatre C, Roubille F, Vernhet H, Jorgensen C, Pers YM. Cardiac complications attributed to chloroquine and hydroxychloroquine: a systematic review of the literature. *Drug Saf*. 2018;41(10):919–931.
131. Gabay C, Riek M, Hetland ML, et al. Effectiveness of tocilizumab with and without synthetic disease-modifying antirheumatic drugs in rheumatoid arthritis: results from a European collaborative study. A*nn Rheum Dis*. 2016;75(7):1336–1342.
132. Giles JT, Sattar N, Gabriel S, et al. Cardiovascular safety of tocilizumab versus etanercept in rheumatoid arthritis: a randomized controlled trial. *Arthritis Rheumatol*. 2020;72(1):31–40.

**Figure 1. Structural properties of the SARS-CoV-2.** Severe acute respiratory syndrome coronavirus 2 (SARS-CoV-2) consists of five components: an RNA gene chain and four proteins. The outermost layer is spike (S) protein, with a molecular weight of 180,000-200,000, which is composed of 1,200-1,500 amino acids and contains 21-35 N-glycosylation sites. Several S proteins form a special spiky corolla structure on the virus surface in the form of trimer (about 600 kDa). The main function of S protein is to bind to the angiotensin converting enzyme 2 (ACE2) receptor on the surface of human cells. It is one of the largest class I fusion proteins known, which enables virus particles to fuse into cells for replication and produce more next-generation virus particles. The envelope (E) protein and membrane (M) glycoprotein below the spike are components of the envelope of virus particles, which protect the RNA gene chain inside the virus. M protein is also involved in the assembly and release of next generation virus particles, which plays an important role in the structural stability and functional expression of S, E and N proteins. Inside the virus is a spiral nucleoprotein core composed of RNA gene chain and nucleocapsid protein (N). N protein plays an important role in virus replication. RNA gene chain is an RNA chain compounded in N protein, which is composed of 29,891 nucleotides (about 30,000 nucleotides) in series. Nucleotide G and nucleotide C account for about 40%. The main function of RNA gene chain is to preserve the genetic code of the virus so that the next generation of virus particles can be replicated.

**Figure 2. Names and distributions of 12 open reading frames from 5' to 3' ends of SARS CoV-2 gene and the number of translated amino acids.** The number in brackets is the number of amino acids in translation. The S protein contains S1 and S2 subunits and a single transmembrane (TM) anchor. S protein binds to the angiotensin converting enzyme 2 (ACE2) receptor on the cell surface through the receptor binding domain (RBD), which is an essential step in membrane fusion. Activation of S requires Furin protease like protease to cleave S1/S2 and undergo conformational changes from pre fusion to post fusion. At present, several pre fusion conformations of S protein are known, among which three RBDs show different directions, namely "up" or "down". The receptor binding sites are exposed only when RBD adopts the "up" conformation.

**Figure 3. Cardiovascular damage of the SARS-CoV-2 infection.**

**References**

Adu-Amankwaah J, Mprah R, Adekunle AO, Ndzie Noah ML, Adzika GK, Machuki JO, Sun H. The cardiovascular aspect of COVID-19. Ann Med. 2021;53(1):227–36.

Adu-Amankwaah J. Behind the shadows: Bringing the cardiovascular secrets of long COVID into light. Eur J Prev Cardiol. 2024 Mar 5:zwae098. doi: 10.1093/eurjpc/zwae098.

Alblaihed L, Brady WJ, Al-Salamah T, Mattu A. Dysrhythmias associated with COVID-19: Review and management considerations. Am J Emerg Med. 2023;64:161–8.

AlGhatrif M, Cingolani O, Lakatta EG. The dilemma of coronavirus disease 2019, aging, and cardiovascular disease: insights from cardiovascular aging science. JAMA Cardiol. 2020;5(7):747–8.

Ali MAM, Spinler SA. COVID-19 and thrombosis: From bench to bedside. Trends Cardiovasc Med. 2021;31(3):143–60.

Al-Kuraishy HM, Hussien NR, Al-Niemi MS, Fahad EH, Al-Buhadily AK, Al-Gareeb AI, Al-Hamash SM, Tsagkaris C, Papadakis M, Alexiou A, Batiha GE. SARS-CoV-2 induced HDL dysfunction may affect the host's response to and recovery from COVID-19. Immun Inflamm Dis. 2023;11(5):e861.

Alwani M, Yassin A, Al-Zoubi RM, Aboumarzouk OM, Nettleship J, Kelly D, Al-Qudimat AR, Shabsigh R. Sex-based differences in severity and mortality in COVID-19. Rev Med Virol. 2021;31(6):e2223.

An X, Duan L, Zhang YH, Jin D, Zhao S, Zhou RR, Duan Y, Lian F, Tong X. The three syndromes and six Chinese patent medicine study during the recovery phase of COVID-19. Chin Med. 2021;16(1):44.

Andersen KG, Rambaut A, Lipkin WI, Holmes EC, Garry RF. The proximal origin of SARS-CoV-2. Nat Med. 2020;26(4):450–2.

Antonelli M, Penfold RS, Merino J, Sudre CH, Molteni E, Berry S, Canas LS, Graham MS, Klaser K, Modat M, Murray B, Kerfoot E, Chen L, Deng J, Österdahl MF, Cheetham NJ, Drew DA, Nguyen LH, Pujol JC, Hu C, Selvachandran S, Polidori L, May A, Wolf J, Chan AT, Hammers A, Duncan EL, Spector TD, Ourselin S, Steves CJ. Risk factors and disease profile of post-vaccination SARS-CoV-2 infection in UK users of the COVID symptom study app: a prospective, community-based, nested, case-control study. Lancet Infect Dis. 2022;22(1):43–55.

Arentz M, Yim E, Klaff L, Lokhandwala S, Riedo FX, Chong M, Lee M. Characteristics and outcomes of 21 critically ill patients with COVID-19 in Washington state. JAMA. 2020;323(16):1612–4.

Arsh H, Manoj Kumar F, Simran F, Tamang S, Rehman MU, Ahmed G, Khan M, Malik J, Mehmoodi A. [Role of PCSK9 inhibition during the inflammatory stage of SARS-COV-2: an updated review.](https://pubmed.ncbi.nlm.nih.gov/38333263/) Ann Med Surg (Lond). 2024;86(2):899–908.

Babapoor-Farrokhran S, Gill D, Walker J, Rasekhi RT, Bozorgnia B, Amanullah A. Myocardial injury and COVID-19: possible mechanisms. Life Sci. 2020;253:117723.

Barrett CD, Moore HB, Yaffe MB, Moore EE. ISTH interim guidance on recognition and management of coagulopathy in COVID-19: A comment. J Thromb Haemost. 2020;18(8):2060–3.

Baytuğan NZ, Kandemir HÇ, Bezgin T. In-hospital outcomes of ST-segment elevation myocardial infarction in COVID-19 positive patients undergoing primary percutaneous intervention. Arq Bras Cardiol. 2024;121(1):e20230258.

Beaney T, Burrell LM, Castillo RR, Charchar FJ, Cro S, Damasceno A, Kruger R, Nilsson PM, Prabhakaran D, Ramirez AJ, Schlaich MP, Schutte AE, Tomaszewski M, Touyz R, Wang JG, Weber MA, Poulter NR; MMM Investigators. May Measurement Month 2018: a pragmatic global screening campaign to raise awareness of blood pressure by the International Society of Hypertension. Eur Heart J. 2019;40(25):2006–17.

Bechmann N, Barthel A, Schedl A, Herzig S, Varga Z, Gebhard C, Mayr M, Hantel C, Beuschlein F, Wolfrum C, Perakakis N, Poston L, Andoniadou CL, Siow R, Gainetdinov RR, Dotan A, Shoenfeld Y, Mingrone G, Bornstein SR. Sexual dimorphism in COVID-19: potential clinical and public health implications. Lancet Diabetes Endocrinol. 2022;10(3):221–30.

Benvenuto D, Giovanetti M, Ciccozzi A, Spoto S, Angeletti S, Ciccozzi M. The 2019-new coronavirus epidemic: Evidence for virus evolution. J Med Virol. 2020;92(4):455–9.

Bhatla A, Mayer MM, Adusumalli S, Hyman MC, Oh E, Tierney A, Moss J, Chahal AA, Anesi G, Denduluri S, Domenico CM, Arkles J, Abella BS, Bullinga JR, Callans DJ, Dixit S, Epstein AE, Frankel DS, Garcia FC, Kumareswaram R, Nazarian S, Riley MP, Santangeli P, Schaller RD, Supple GE, Lin D, Marchlinski F, Deo R. COVID-19 and cardiac arrhythmias. Heart Rhythm. 2020;17(9):1439–44.

Bienvenu LA, Noonan J, Wang X, Peter K. Higher mortality of COVID-19 in males: sex differences in immune response and cardiovascular comorbidities. Cardiovasc Res. 2020;116(14):2197–206.

Bikdeli B, Madhavan MV, Jimenez D, Chuich T, Dreyfus I, Driggin E, Nigoghossian C, Ageno W, Madjid M, Guo Y, Tang LV, Hu Y, Giri J, Cushman M, Quéré I, Dimakakos EP, Gibson CM, Lippi G, Favaloro EJ, Fareed J, Caprini JA, Tafur AJ, Burton JR, Francese DP, Wang EY, Falanga A, McLintock C, Hunt BJ, Spyropoulos AC, Barnes GD, Eikelboom JW, Weinberg I, Schulman S, Carrier M, Piazza G, Beckman JA, Steg PG, Stone GW, Rosenkranz S, Goldhaber SZ, Parikh SA, Monreal M, Krumholz HM, Konstantinides SV, Weitz JI, Lip GYH; Global COVID-19 Thrombosis Collaborative Group, Endorsed by the ISTH, NATF, ESVM, and the IUA, Supported by the ESC Working Group on Pulmonary Circulation and Right Ventricular Function. COVID-19 and thrombotic or thromboembolic disease: implications for prevention, antithrombotic therapy, and follow-up: JACC state-of-the-art review. J Am Coll Cardiol. 2020;75(23):2950–73.

Blyszczuk P. Myocarditis in humans and in experimental animal models. Front Cardiovasc Med. 2019;6:64.

Bonanad C, García-Blas S, Tarazona-Santabalbina F, Sanchis J, Bertomeu-González V, Fácila L, Ariza A, Núñez J, Cordero A. The effect of age on mortality in patients with COVID-19: a meta-analysis with 611,583 subjects. J Am Med Dir Assoc. 2020;21(7):915–8.

Bonow RO, Fonarow GC, O’Gara PT, Yancy CW. Association of coronavirus disease 2019 (COVID-19) with myocardial injury and mortality. JAMA Cardiol. 2020;5(7):751–3.

Bramante CT, Buse JB, Liebovitz DM, Nicklas JM, Puskarich MA, Cohen K, Belani HK, Anderson BJ, Huling JD, Tignanelli CJ, Thompson JL, Pullen M, Wirtz EL, Siegel LK, Proper JL, Odde DJ, Klatt NR, Sherwood NE, Lindberg SM, Karger AB, Beckman KB, Erickson SM, Fenno SL, Hartman KM, Rose MR, Mehta T, Patel B, Griffiths G, Bhat NS, Murray TA, Boulware DR; COVID-OUT Study Team. Outpatient treatment of COVID-19 and incidence of post-COVID-19 condition over 10 months (COVID-OUT): a multicentre, randomised, quadruple-blind, parallel-group, phase 3 trial. Lancet Infect Dis. 2023;23(10):1119–29.

Bramante CT, Huling JD, Tignanelli CJ, Buse JB, Liebovitz DM, Nicklas JM, Cohen K, Puskarich MA, Belani HK, Proper JL, Siegel LK, Klatt NR, Odde DJ, Luke DG, Anderson B, Karger AB, Ingraham NE, Hartman KM, Rao V, Hagen AA, Patel B, Fenno SL, Avula N, Reddy NV, Erickson SM, Lindberg S, Fricton R, Lee S, Zaman A, Saveraid HG, Tordsen WJ, Pullen MF, Biros M, Sherwood NE, Thompson JL, Boulware DR, Murray TA; COVID-OUT Trial Team. Randomized trial of metformin, ivermectin, and fluvoxamine for COVID-19. N Engl J Med. 2022;387(7):599–610.

Brandi ML. Are sex hormones promising candidates to explain sex disparities in the COVID-19 pandemic? Rev Endocr Metab Disord. 2022;23(2):171–83.

Bugiardini R, Nava S, Caramori G, Yoon J, Badimon L, Bergami M, Cenko E, David A, Demiri I, Dorobantu M, Fronea O, Jankovic R, Kedev S, Ladjevic N, Lasica R, Loncar G, Mancuso G, Mendieta G, Miličić D, Mjehović P, Pašalić M, Petrović M, Poposka L, Scarpone M, Stefanovic M, van der Schaar M, Vasiljevic Z, Vavlukis M, Vega Pittao ML, Vukomanovic V, Zdravkovic M, Manfrini O. Sex differences and disparities in cardiovascular outcomes of COVID-19. Cardiovasc Res. 2023;119(5):1190–201.

Burger AL, Kaufmann CC, Jäger B, Pogran E, Ahmed A, Wojta J, Farhan S, Huber K. Direct cardiovascular complications and indirect collateral damage during the COVID-19 pandemic: A review. Wien Klin Wochenschr. 2021;133(23-24):1289–97.

Butler CC, Hobbs FDR, Gbinigie OA, Rahman NM, Hayward G, Richards DB, Dorward J, Lowe DM, Standing JF, Breuer J, Khoo S, Petrou S, Hood K, Nguyen-Van-Tam JS, Patel MG, Saville BR, Marion J, Ogburn E, Allen J, Rutter H, Francis N, Thomas NPB, Evans P, Dobson M, Madden TA, Holmes J, Harris V, Png ME, Lown M, van Hecke O, Detry MA, Saunders CT, Fitzgerald M, Berry NS, Mwandigha L, Galal U, Mort S, Jani BD, Hart ND, Ahmed H, Butler D, McKenna M, Chalk J, Lavallee L, Hadley E, Cureton L, Benysek M, Andersson M, Coates M, Barrett S, Bateman C, Davies JC, Raymundo-Wood I, Ustianowski A, Carson-Stevens A, Yu LM, Little P; PANORAMIC Trial Collaborative Group. Molnupiravir plus usual care versus usual care alone as early treatment for adults with COVID-19 at increased risk of adverse outcomes (PANORAMIC): an open-label, platform-adaptive randomised controlled trial. Lancet. 2023;401(10373):281–93.

Caforio ALP, Baritussio A, Basso C, Marcolongo R. Clinically suspected and biopsy-proven myocarditis temporally associated with SARS-CoV-2 infection. Annu Rev Med. 2022;73:149–66.

Callaway E, Cyranoski D, Mallapaty S, Stoye E, Tollefson J. The coronavirus pandemic in five powerful charts. Nature. 2020;579(7800):482–3.

Castiello T, Georgiopoulos G, Finocchiaro G, Claudia M, Gianatti A, Delialis D, Aimo A, Prasad S. COVID-19 and myocarditis: a systematic review and overview of current challenges. Heart Fail Rev. 2022;27(1):251–61.

Ceraolo C, Giorgi FM. Genomic variance of the 2019-nCoV coronavirus. J Med Virol. 2020;92(5):522–8.

Chan JF, Kok KH, Zhu Z, Chu H, To KK, Yuan S, Yuen KY. Genomic characterization of the 2019 novel human-pathogenic coronavirus isolated from a patient with atypical pneumonia after visiting Wuhan. Emerg Microbes Infect. 2020a;9(1):221–36.

Chan JF, Yuan S, Kok KH, To KK, Chu H, Yang J, Xing F, Liu J, Yip CC, Poon RW, Tsoi HW, Lo SK, Chan KH, Poon VK, Chan WM, Ip JD, Cai JP, Cheng VC, Chen H, Hui CK, Yuen KY. A familial cluster of pneumonia associated with the 2019 novel coronavirus indicating person-to-person transmission: a study of a family cluster. Lancet. 2020b;395(10223):514–23.

Chappell MC. Renin-angiotensin system and sex differences in COVID-19: a critical assessment. Circ Res. 2023;132(10):1320–37.

Chatre C, Roubille F, Vernhet H, Jorgensen C, Pers YM. Cardiac complications attributed to chloroquine and hydroxychloroquine: a systematic review of the literature. Drug Saf. 2018;41(10):919–31.

Chen C, Zhou Y, Wang DW. SARS-CoV-2: a potential novel etiology of fulminant myocarditis. Herz. 2020a;45(3):230–2.

Chen H, Chen C, Spanos M, Li G, Lu R, Bei Y, Xiao J. Exercise training maintains cardiovascular health: signaling pathways involved and potential therapeutics. Signal Transduct Target Ther. 2022;7(1):306.

Chen L, Li X, Chen M, Feng Y, Xiong C. The ACE2 expression in human heart indicates new potential mechanism of heart injury among patients infected with SARS-CoV-2. Cardiovasc Res. 2020b;116(6):1097–100.

Chen N, Zhou M, Dong X, Qu J, Gong F, Han Y, Qiu Y, Wang J, Liu Y, Wei Y, Xia J, Yu T, Zhang X, Zhang L. Epidemiological and clinical characteristics of 99 cases of 2019 novel coronavirus pneumonia in Wuhan, China: a descriptive study. Lancet. 2020c;395(10223):507–13.

Chen T, Wu D, Chen H, Yan W, Yang D, Chen G, Ma K, Xu D, Yu H, Wang H, Wang T, Guo W, Chen J, Ding C, Zhang X, Huang J, Han M, Li S, Luo X, Zhao J, Ning Q. Clinical characteristics of 113 deceased patients with coronavirus disease 2019: retrospective study. BMJ. 2020d;368:m1091.

Chidambaram V, Kumar A, Majella MG, Seth B, Sivakumar RK, Voruganti D, Bavineni M, Baghal A, Gates K, Kumari A, Al'Aref SJ, Galiatsatos P, Karakousis PC, Mehta JL. HDL cholesterol levels and susceptibility to COVID-19. EBioMedicine. 2022a;82:104166.

Chidambaram V, Shanmugavel Geetha H, Kumar A, Majella MG, Sivakumar RK, Voruganti D, Mehta JL, Karakousis PC. Association of lipid levels with COVID-19 infection, disease severity and mortality: a systematic review and meta-analysis. Front Cardiovasc Med. 2022b;9:862999.

Chimenti C, Magnocavallo M, Ballatore F, Bernardini F, Alfarano M, Della Rocca DG, Severino P, Lavalle C, Francesco F, Frustaci A. Prevalence and clinical implications of COVID-19 myocarditis. Card Electrophysiol Clin. 2022;14(1):53–62.

Chiu JS, Lahoud-Rahme M, Schaffer D, Cohen A, Samuels-Kalow M. Kawasaki disease features and myocarditis in a patient with COVID-19. Pediatr Cardiol. 2020;41(7):1526–8.

Choi HS, Choi AY, Kopp JB, Winkler CA, Cho SK.J Review of COVID-19 therapeutics by mechanism: from discovery to approval. Korean Med Sci. 2024;39(14):e134. doi: 10.3346/jkms.2024.39.e134.

Chung MK, Zidar DA, Bristow MR, Cameron SJ, Chan T, Harding CV 3rd, Kwon DH, Singh T, Tilton JC, Tsai EJ, Tucker NR, Barnard J, Loscalzo J. COVID-19 and Cardiovascular Disease: From Bench to Bedside. Circ Res. 2021;128(8):1214–36.

Cole JE, Park I, Ahern DJ, Kassiteridi C, Danso Abeam D, Goddard ME, Green P, Maffia P, Monaco C. Immune cell census in murine atherosclerosis: cytometry by time of flight illuminates vascular myeloid cell diversity. Cardiovasc Res. 2018;114(10):1360–71.

Colzani M, Bargehr J, Mescia F, Williams EC, Knight-Schrijver V, Lee J, Summers C, Mohorianu I, Smith KGC, Lyons PA, Sinha S. Proinflammatory cytokines driving cardiotoxicity in COVID-19. Cardiovasc Res. 2024;120(2):174–87.

Daniels CJ, Rajpal S, Greenshields JT, Rosenthal GL, Chung EH, Terrin M, Jeudy J, Mattson SE, Law IH, Borchers J, Kovacs R, Kovan J, Rifat SF, Albrecht J, Bento AI, Albers L, Bernhardt D, Day C, Hecht S, Hipskind A, Mjaanes J, Olson D, Rooks YL, Somers EC, Tong MS, Wisinski J, Womack J, Esopenko C, Kratochvil CJ, Rink LD; Big Ten COVID-19 Cardiac Registry Investigators. Prevalence of Clinical and Subclinical Myocarditis in Competitive Athletes With Recent SARS-CoV-2 Infection: Results From the Big Ten COVID-19 Cardiac Registry. JAMA Cardiol. 2021;6(9):1078–87.

Danser AHJ, Epstein M, Batlle D. Renin-angiotensin system blockers and the COVID-19 pandemic: at present there is no evidence to abandon renin-angiotensin system blockers. Hypertension. 2020;75(6):1382–5.

Danzi GB, Loffi M, Galeazzi G, Gherbesi E. Acute pulmonary embolism and COVID-19 pneumonia: a random association? Eur Heart J. 2020;41(19):1858.

Davis MG, Bobba A, Majeed H, Bilal MI, Nasrullah A, Ratmeyer GM, Chourasia P, Gangu K, Farooq A, Avula SR, Sheikh AB. COVID-19 with stress cardiomyopathy mortality and outcomes among patients hospitalized in the united states: a propensity matched analysis using the national inpatient sample database. Curr Probl Cardiol. 2023;48(5):101607.

Del Vecchio L, Balafa O, Dounousi E, Ekart R, Fernandez BF, Mark PB, Sarafidis P, Valdivielso JM, Ferro CJ, Mallamaci F. COVID-19 and cardiovascular disease in patients with chronic kidney disease. Nephrol Dial Transplant. 2024;39(2):177–89.

Dherange P, Lang J, Qian P, Oberfeld B, Sauer WH, Koplan B, Tedrow U. Arrhythmias and COVID-19: A Review. JACC Clin Electrophysiol. 2020;6(9):1193–204.

Dobrowolska K, Zarębska-Michaluk D, Poniedziałek B, Jaroszewicz J, Flisiak R, Rzymski P. Overview of autoantibodies in COVID-19 convalescents. J Med Virol. 2023;95(6):e28864.

Dogan Z, Erden I, Bektasoglu G, Karabulut A. Association between history of polymerase chain reaction-verified COVID-19 infection and outcomes of subsequent ST-elevation myocardial infarction. Angiology. 2024;75(2):131–8.

Drummond GR, Vinh A, Guzik TJ, Sobey CG. Immune mechanisms of hypertension. Nat Rev Immunol. 2019;19(8):517–32.

Epelbaum O. Autopsy findings and venous thromboembolism in patients with COVID-19. Ann Intern Med. 2020;173(12):1029–30.

Epidemiology Working Group for NCIP Epidemic Response, Chinese Center for Disease Control and Prevention. Epidemiology Working Group for NCIP Epidemic Response, Chinese Center for Disease Control and Prevention. The epidemiological characteristics of an outbreak of 2019 novel coronavirus diseases (COVID-19) in China. Zhonghua Liu Xing Bing Xue Za Zhi. 2020;41(2):145–51.

Fairweather D, Beetler DJ, Di Florio DN, Musigk N, Heidecker B, Cooper LT Jr. COVID-19, Myocarditis and pericarditis. Circ Res. 2023;132(10):1302–19.

Farkouh ME, Stone GW, Lala A, Bagiella E, Moreno PR, Nadkarni GN, Ben-Yehuda O, Granada JF, Dressler O, Tinuoye EO, Granada C, Bustamante J, Peyra C, Godoy LC, Palacios IF, Fuster V. Anticoagulation in Patients With COVID-19: JACC Review Topic of the Week. J Am Coll Cardiol. 2022;79(9):917–28.

Finsterer J, Scorza FA, Scorza CA, Fiorini AC. Ischemic stroke in 455 COVID-19 patients. Clinics (Sao Paulo). 2022;77:100012.

Fodor A, Tiperciuc B, Login C, Orasan OH, Lazar AL, Buchman C, Hanghicel P, Sitar-Taut A, Suharoschi R, Vulturar R, Cozma A. Endothelial dysfunction, inflammation, and oxidative stress in COVID-19-mechanisms and therapeutic targets. Oxid Med Cell Longev. 2021;2021:8671713.

Fried JA, Ramasubbu K, Bhatt R, Topkara VK, Clerkin KJ, Horn E, Rabbani L, Brodie D, Jain SS, Kirtane AJ, Masoumi A, Takeda K, Kumaraiah D, Burkhoff D, Leon M, Schwartz A, Uriel N, Sayer G. The variety of cardiovascular presentations of COVID-19. Circulation. 2020;141(23):1930–6.

Fung KW, Baye F, Baik SH, McDonald CJ. Nirmatrelvir and molnupiravir and post–COVID-19 condition in older patients. JAMA Intern Med. 2023;183(12):1404–6.

Gabay C, Riek M, Hetland ML, Hauge EM, Pavelka K, Tomšič M, Canhao H, Chatzidionysiou K, Lukina G, Nordström DC, Lie E, Ancuta I, Hernández MV, van Riel PL, van Vollenhoven R, Kvien TK. Effectiveness of tocilizumab with and without synthetic disease-modifying antirheumatic drugs in rheumatoid arthritis: results from a European collaborative study. Ann Rheum Dis. 2016;75(7):1336-42. doi: 10.1136/annrheumdis-2015-207760.

García-Escobar A, Vera-Vera S, Jurado-Román A, Jiménez-Valero S, Galeote G, Moreno R. Calcium signaling pathway is involved in the shedding of ACE2 catalytic ectodomain: new insights for clinical and therapeutic applications of ACE2 for COVID-19. Biomolecules. 2022;12(1):76.

Gąsecka A, Borovac JA, Guerreiro RA, Giustozzi M, Parker W, Caldeira D, Chiva-Blanch G. Thrombotic complications in patients with COVID-19: pathophysiological mechanisms, diagnosis, and treatment. Cardiovasc Drugs Ther. 2021;35(2):215–29.

Gautret P, Lagier JC, Parola P, Hoang VT, Meddeb L, Mailhe M, Doudier B, Courjon J, Giordanengo V, Vieira VE, Tissot Dupont H, Honoré S, Colson P, Chabrière E, La Scola B, Rolain JM, Brouqui P, Raoult D. Hydroxychloroquine and azithromycin as a treatment of COVID-19: results of an open- label non- randomized clinical trial. Int J Antimicrob Agents. 2020;56(1):105949.

Gebhard C, Regitz-Zagrosek V, Neuhauser HK, Morgan R, Klein SL. Impact of sex and gender on COVID-19 outcomes in Europe. Biol Sex Differ. 2020;11(1):29.

Gharibzadeh A, Shahsanaei F, Rahimi Petrudi N. Clinical and cardiovascular characteristics of patients suffering ST-segment elevation myocardial infarction after COVID-19: a systematic review and meta-analysis. Curr Probl Cardiol. 2023;48(1):101045.

Gheblawi M, Wang K, Viveiros A, Nguyen Q, Zhong JC, Turner AJ, Raizada MK, Grant MB, Oudit GY. Angiotensin-converting enzyme 2: SARS-CoV-2 receptor and regulator of the renin-angiotensin system: celebrating the 20th Anniversary of the Discovery of ACE2. Circ Res. 2020;126(10):1456–74.

Giles JT, Sattar N, Gabriel S, Ridker PM, Gay S, Warne C, Musselman D, Brockwell L, Shittu E, Klearman M, Fleming TR. Cardiovascular safety of tocilizumab versus etanercept in rheumatoid arthritis: a randomized controlled trial. Arthritis Rheumatol. 2020;72(1):31–40.

Giudicessi JR, Noseworthy PA, Friedman PA, Ackerman MJ. Urgent guidance for navigating and circumventing the QTc-prolonging and torsadogenic potential of possible pharmacotherapies for coronavirus disease 19 (COVID-19). Mayo Clin Proc. 2020;95(6):1213–21.

Giugni FR, Duarte-Neto AN, da Silva LFF, Monteiro RAA, Mauad T, Saldiva PHN, Dolhnikoff M. Younger age is associated with cardiovascular pathological phenotype of severe COVID-19 at autopsy. Front Med (Lausanne). 2024;10:1327415.

Gnecchi M, Moretti F, Bassi EM, Leonardi S, Totaro R, Perotti L, Zuccaro V, Perlini S, Preda L, Baldanti F, Bruno R, Visconti LO. Myocarditis in a 16-year-old boy positive for SARS-CoV-2. Lancet. 2020;395(10242):e116.

Gross CP, Essien UR, Pasha S, Gross JR, Wang SY, Nunez-Smith M. Racial and ethnic disparities in population-level COVID-19 mortality. J Gen Intern Med. 2020;35(10):3097–9.

Guan WJ, Ni ZY, Hu Y, Liang WH, Ou CQ, He JX, Liu L, Shan H, Lei CL, Hui DSC, Du B, Li LJ, Zeng G, Yuen KY, Chen RC, Tang CL, Wang T, Chen PY, Xiang J, Li SY, Wang JL, Liang ZJ, Peng YX, Wei L, Liu Y, Hu YH, Peng P, Wang JM, Liu JY, Chen Z, Li G, Zheng ZJ, Qiu SQ, Luo J, Ye CJ, Zhu SY, Zhong NS; China Medical Treatment Expert Group for Covid-19. Clinical Characteristics of Coronavirus Disease 2019 in China. N Engl J Med. 2020;382(18):1708–20.

Guo J, Huang Z, Lin L, Lv J. Coronavirus Disease 2019 (COVID-19) and Cardiovascular Disease: A Viewpoint on the Potential Influence of Angiotensin- Converting Enzyme Inhibitors/Angiotensin Receptor Blockers on Onset and Severity of Severe Acute Respiratory Syndrome Coronavirus 2 Infection. J Am Heart Assoc. 2020a;9(7):e016219.

Guo T, Fan Y, Chen M, Wu X, Zhang L, He T, Wang H, Wan J, Wang X, Lu Z. Cardiovascular implications of fatal outcomes of patients with coronavirus disease 2019 (COVID-19). JAMA Cardiol. 2020b;5(7):811–8.

Guzik TJ, Mohiddin SA, Dimarco A, Patel V, Savvatis K, Marelli-Berg FM, Madhur MS, Tomaszewski M, Maffia P, D'Acquisto F, Nicklin SA, Marian AJ, Nosalski R, Murray EC, Guzik B, Berry C, Touyz RM, Kreutz R, Wang DW, Bhella D, Sagliocco O, Crea F, Thomson EC, McInnes IB. COVID-19 and the cardiovascular system: implications for risk assessment, diagnosis, and treatment options. Cardiovasc Res. 2020;116(10):1666–87.

Gyöngyösi M, Alcaide P, Asselbergs FW, Brundel BJJM, Camici GG, Martins PDC, Ferdinandy P, Fontana M, Girao H, Gnecchi M, Gollmann-Tepeköylü C, Kleinbongard P, Krieg T, Madonna R, Paillard M, Pantazis A, Perrino C, Pesce M, Schiattarella GG, Sluijter JPG, Steffens S, Tschöpe C, Van Linthout S, Davidson SM. Long COVID and the cardiovascular system-elucidating causes and cellular mechanisms in order to develop targeted diagnostic and therapeutic strategies: a joint Scientific Statement of the ESC Working Groups on Cellular Biology of the Heart and Myocardial and Pericardial Diseases. Cardiovasc Res. 2023;119(2):336–56.

Haitao T, Vermunt JV, Abeykoon J, Ghamrawi R, Gunaratne M, Jayachandran M, Narang K, Parashuram S, Suvakov S, Garovic VD. COVID-19 and sex differences: mechanisms and biomarkers. Mayo Clin Proc. 2020;95(10):2189–203.

Hajra A, Malik A, Bandyopadhyay D, Goel A, Isath A, Gupta R, Krishnan S, Rai D, Krittanawong C, Virani SS, Fonarow GC, Lavie CJ. Impact of COVID-19 in patients hospitalized with stress cardiomyopathy: A nationwide analysis. Prog Cardiovasc Dis. 2023;76:25–30.

Hakobyan N, Ilerhunmwuwa N, Wasifuddin M, Tasnim A, Boris A. COVID-19-associated disseminated intravascular coagulopathy presenting as inferior ST-segment elevation myocardial infarction. Cureus. 2023;15(5):e39308.

Hanna A, Frangogiannis NG. Inflammatory cytokines and chemokines as therapeutic targets in heart failure. Cardiovasc Drugs Ther. 2020;34(6):849–63.

Harrison SL, Buckley BJR, Rivera-Caravaca JM, Zhang J, Lip GYH. Cardiovascular risk factors, cardiovascular disease, and COVID-19: an umbrella review of systematic reviews. Eur Heart J Qual Care Clin Outcomes. 2021;7(4):330–9.

Hatch CJ, Piombo SD, Fang JS, Gach JS, Ewald ML, Van Trigt WK, Coon BG, Tong JM, Forthal DN, Hughes CCW. SARS-CoV-2 infection of endothelial cells, dependent on flow-induced ACE2 expression, drives hypercytokinemia in a vascularized microphysiological system. Front Cardiovasc Med. 2024;11:1360364.

Hati S, Bhattacharyya S. Impact of thiol-disulfide balance on the binding of COVID-19 spike protein with angiotensin-converting enzyme 2 receptor. ACS Omega. 2020;5(26):16292–8.

Heinrich F, Roedl K, Jarczak D, Goebels HL, Heinemann A, Schäfer U, Ludwig F, Bachmann M, Bein B, Weber CF, Sydow K, Bota M, Paschen HR, de Weerth A, Veit C, Detsch O, Brand PA, Kluge S, Ondruschka B, Wichmann D. New insights in the occurrence of venous thromboembolism in critically ill patients with COVID-19-a large postmortem and clinical analysis. Viruses. 2022;14(4):811.

Hendren NS, Drazner MH, Bozkurt B, Cooper LT Jr. Description and Proposed Management of the Acute COVID-19 Cardiovascular Syndrome. Circulation. 2020;141(23):1903–14.

Ho JS, Sia CH, Chan MY, Lin W, Wong RC. Coronavirus-induced myocarditis: A meta-summary of cases. Heart Lung. 2020;49(6):681–5.

Hobohm L, Sagoschen I, Barco S, Farmakis IT, Fedeli U, Koelmel S, Gori T, Espinola-Klein C, Münzel T, Konstantinides S, Keller K. COVID-19 infection and its impact on case fatality in patients with pulmonary embolism. Eur Respir J. 2023;61(1):2200619.

Hoffmann M, Kleine-Weber H, Schroeder S, Krüger N, Herrler T, Erichsen S, Schiergens TS, Herrler G, Wu NH, Nitsche A, Müller MA, Drosten C, Pöhlmann S. SARS-CoV-2 cell entry depends on ACE2 and TMPRSS2 and is blocked by a clinically proven protease inhibitor. Cell. 2020;181(2):271–80.e8.

Holshue ML, DeBolt C, Lindquist S, Lofy KH, Wiesman J, Bruce H, Spitters C, Ericson K, Wilkerson S, Tural A, Diaz G, Cohn A, Fox L, Patel A, Gerber SI, Kim L, Tong S, Lu X, Lindstrom S, Pallansch MA, Weldon WC, Biggs HM, Uyeki TM, Pillai SK; Washington State 2019-nCoV Case Investigation Team. First case of 2019 novel coronavirus in the United States. N Engl J Med. 2020;382(10):929–36.

Hooper NM, Lambert DW, Turner AJ. Discovery and characterization of ACE2 - a 20-year journey of surprises from vasopeptidase to COVID-19. Clin Sci (Lond). 2020;134(18):2489–501.

Hu H, Ma F, Wei X, Fang Y. Coronavirus fulminant myocarditis saved with glucocorticoid and human immunoglobulin. Eur Heart J. 2021;42(2):206.

Huang C, Wang Y, Li X, Ren L, Zhao J, Hu Y, Zhang L, Fan G, Xu J, Gu X, Cheng Z, Yu T, Xia J, Wei Y, Wu W, Xie X, Yin W, Li H, Liu M, Xiao Y, Gao H, Guo L, Xie J, Wang G, Jiang R, Gao Z, Jin Q, Wang J, Cao B. Clinical features of patients infected with 2019 novel coronavirus in Wuhan, China. Lancet. 2020;395(10223):497–506.

Inciardi RM, Lupi L, Zaccone G, Italia L, Raffo M, Tomasoni D, Cani DS, Cerini M, Farina D, Gavazzi E, Maroldi R, Adamo M, Ammirati E, Sinagra G, Lombardi CM, Metra M. Cardiac involvement in a patient with coronavirus disease 2019 (COVID-19). JAMA Cardiol. 2020;5(7):819–824.

Irizar P, Pan D, Kapadia D, Bécares L, Sze S, Taylor H, Amele S, Kibuchi E, Divall P, Gray LJ, Nellums LB, Katikireddi SV, Pareek M. Ethnic inequalities in COVID-19 infection, hospitalisation, intensive care admission, and death: a global systematic review and meta-analysis of over 200 million study participants. EClinicalMedicine. 2023;57:101877.

Isath A, Malik A, Bandyopadhyay D, Goel A, Hajra A, Dhand A, Lanier GM, Fonarow GC, Lavie CJ, Gass AL. COVID-19, heart failure hospitalizations, and outcomes: a nationwide analysis. Curr Probl Cardiol. 2023;48(4):101541.

Jahanshahi F, Jazayeri SB, Eraghi MM, Reis LO, Hamidikia M, Amiri S, Aghamir SMK. [A narrative review on adverse drug reactions of COVID-19 treatments on the kidney.](https://pubmed.ncbi.nlm.nih.gov/38584847/) Open Med (Wars). 2024;19(1):20230867.

Jiang S, Xia S, Ying T, Lu L. A novel coronavirus (2019-nCoV) causing pneumonia-associated respiratory syndrome. Cell Mol Immunol. 2020;17(5):554.

Jin YH, Cai L, Cheng ZS, Cheng H, Deng T, Fan YP, Fang C, Huang D, Huang LQ, Huang Q, Han Y, Hu B, Hu F, Li BH, Li YR, Liang K, Lin LK, Luo LS, Ma J, Ma LL, Peng ZY, Pan YB, Pan ZY, Ren XQ, Sun HM, Wang Y, Wang YY, Weng H, Wei CJ, Wu DF, Xia J, Xiong Y, Xu HB, Yao XM, Yuan YF, Ye TS, Zhang XC, Zhang YW, Zhang YG, Zhang HM, Zhao Y, Zhao MJ, Zi H, Zeng XT, Wang YY, Wang XH; for the Zhongnan Hospital of Wuhan University Novel Coronavirus Management and Research Team, Evidence-Based Medicine Chapter of China International Exchange and Promotive Association for Medical and Health Care (CPAM). A rapid advice guideline for the diagnosis and treatment of 2019 novel coronavirus (2019-nCoV) infected pneumonia (standard version). Mil Med Res. 2020;7(1):4.

Julius U, Schatz U, Tselmin S, Morawietz H. COVID-19 and lipid disorders. Horm Metab Res. 2022;54(8):514–21.

Kang X, Jin D, Jiang L, Zhang Y, Zhang Y, An X, Duan L, Yang C, Zhou R, Duan Y, Sun Y, Lian F. Efficacy and mechanisms of traditional Chinese medicine for COVID-19: a systematic review. Chin Med. 2022;17(1):30.

Kang Y, Chen T, Mui D, Ferrari V, Jagasia D, Scherrer-Crosbie M, Chen Y, Han Y. Cardiovascular manifestations and treatment considerations in covid-19. Heart. 2020;106(15):1132–41.

Karbalaeimahdi M, Farajnia S, Bargahi N, Ghadiri-Moghaddam F, Rasouli Jazi HR, Bakhtiari N, Ghasemali S, Zarghami N. The role of interferons in long COVID infection. J Interferon Cytokine Res. 2023;43(2):65–76.

Katsoularis I, Fonseca-Rodríguez O, Farrington P, Lindmark K, Fors Connolly AM. Risk of acute myocardial infarction and ischaemic stroke following COVID-19 in Sweden: a self-controlled case series and matched cohort study. Lancet. 2021;398(10300):599–607.

Keller K, Sagoschen I, Konstantinides S, Gori T, Münzel T, Hobohm L. Incidence and risk factors of myocarditis in hospitalized patients with COVID-19. J Med Virol. 2023;95(3):e28646.

Kitsou V, Lunde T, Solholm A, Blomberg B, Saeed S. Complete recovery after fulminant myocarditis in a patient with COVID-19. Pak J Med Sci. 2024;40(4):785–9.

Klok FA, Kruip MJHA, van der Meer NJM, Arbous MS, Gommers DAMPJ, Kant KM, Kaptein FHJ, van Paassen J, Stals MAM, Huisman MV, Endeman H. Incidence of thrombotic complications in critically ill ICU patients with COVID-19. Thromb Res. 2020;191:145–47.

Kochav SM, Coromilas E, Nalbandian A, Ranard LS, Gupta A, Chung MK, Gopinathannair R, Biviano AB, Garan H, Wan EY. Cardiac arrhythmias in COVID-19 infection. Circ Arrhythm Electrophysiol. 2020;13(6):e008719.

Kochi AN, Tagliari AP, Forleo GB, Fassini GM, Tondo C. Cardiac and arrhythmic complications in patients with COVID-19. J Cardiovasc Electrophysiol. 2020;31(5):1003–8.

Kouhpeikar H, Khosaravizade Tabasi H, Khazir Z, Naghipour A, Mohammadi Moghadam H, Forouzanfar H, Abbasifard M, Kirichenko TV, Reiner Ž, Banach M, Sahebkar A. Statin use in COVID-19 hospitalized patients and outcomes: a retrospective study. Front Cardiovasc Med. 2022;9:820260.

Kowalska K, Sabatowska Z, Forycka J, Młynarska E, Franczyk B, Rysz J. The influence of SARS-CoV-2 infection on lipid metabolism-the potential use of lipid-lowering agents in COVID-19 management. Biomedicines. 2022;10(9):2320.

Kreutz R, Algharably E, Azizi M, Dobrowolski P, Guzik T, Januszewicz A, Persu A, Prejbisz A, Riemer T, Wang J, Burnier M. Hypertension, the renin–angiotensin system, and the risk of lower respiratory tract infections and lung injury: implications for COVID-19. European Society of Hypertension COVID-19 Task Force Review of Evidence. Cardiovasc Res. 2020;116(10):1688–99.

Kyriakoulis KG, Kokkinidis DG, Kyprianou IA, Papanastasiou CA, Archontakis-Barakakis P, Doundoulakis I, Bakoyiannis C, Giannakoulas G, Palaiodimos L. Venous thromboembolism in the era of COVID-19. Phlebology. 2021;36(2):91–9.

Lake MA. What we know so far: COVID-19 current clinical knowledge and research. Clin Med (Lond). 2020;20(2):124–7.

Lakkireddy DR, Chung MK, Gopinathannair R, Patton KK, Gluckman TJ, Turagam M, Cheung JW, Patel P, Sotomonte J, Lampert R, Han JK, Rajagopalan B, Eckhardt L, Joglar J, Sandau KE, Olshansky B, Wan E, Noseworthy PA, Leal M, Kaufman E, Gutierrez A, Marine JE, Wang PJ, Russo AM. Guidance for cardiac electrophysiology during the COVID-19 pandemic from the Heart Rhythm Society COVID-19 Task Force; Electrophysiology Section of the American College of Cardiology; and the Electrocardiography and Arrhythmias Committee of the Council on Clinical Cardiology, American Heart Association. Heart Rhythm. 2020;17(9):e233–41.

Lazzerini PE, Laghi-Pasini F, Boutjdir M, Capecchi PL. Cardioimmunology of arrhythmias: the role of autoimmune and inflammatory cardiac channelopathies. Nat Rev Immunol. 2019;19(1):63–4.

Leng Z, Zhu R, Hou W, Feng Y, Yang Y, Han Q, Shan G, Meng F, Du D, Wang S, Fan J, Wang W, Deng L, Shi H, Li H, Hu Z, Zhang F, Gao J, Liu H, Li X, Zhao Y, Yin K, He X, Gao Z, Wang Y, Yang B, Jin R, Stambler I, Lim LW, Su H, Moskalev A, Cano A, Chakrabarti S, Min KJ, Ellison-Hughes G, Caruso C, Jin K, Zhao RC. Transplantation of ACE2- mesenchymal stem cells improves the outcome of patients with COVID-19 pneumonia. Aging Dis. 2020;11(2):216–28.

Li L, Xie H, Wang L, Zhang A, Mou X, Lin Y, Ma H, Wang Y, Li J, Gao J, Wang CC, Leung PC, Fan X, Wu X. The efficacy and safety of combined chinese herbal medicine and western medicine therapy for COVID-19: a systematic review and meta-analysis. Chin Med. 2022;17(1):77.

Li Q, Guan X, Wu P, Wang X, Zhou L, Tong Y, Ren R, Leung KSM, Lau EHY, Wong JY, Xing X, Xiang N, Wu Y, Li C, Chen Q, Li D, Liu T, Zhao J, Liu M, Tu W, Chen C, Jin L, Yang R, Wang Q, Zhou S, Wang R, Liu H, Luo Y, Liu Y, Shao G, Li H, Tao Z, Yang Y, Deng Z, Liu B, Ma Z, Zhang Y, Shi G, Lam TTY, Wu JT, Gao GF, Cowling BJ, Yang B, Leung GM, Feng Z. Early transmission dynamics in Wuhan, China, of novel coronavirus–infected pneumonia. N Engl J Med. 2020;382(13):1199–207.

Lian J, Jin X, Hao S, Cai H, Zhang S, Zheng L, Jia H, Hu J, Gao J, Zhang Y, Zhang X, Yu G, Wang X, Gu J, Ye C, Jin C, Lu Y, Yu X, Yu X, Ren Y, Qiu Y, Li L, Sheng J, Yang Y. Analysis of epidemiological and clinical features in older patients with coronavirus disease 2019 (covid-19) outside Wuhan. Clin Infect Dis. 2020;71(15):740–7.

Liang B, Chen J, Li T, Wu H, Yang W, Li Y, Li J, Yu C, Nie F, Ma Z, Yang M, Xiao M, Nie P, Gao Y, Qian C, Hu M. Clinical remission of a critically ill COVID-19 patient treated by human umbilical cord mesenchymal stem cells: A case report. Medicine (Baltimore). 2020;99(31):e21429.

Liang C, Zhang W, Li S, Qin G. Coronary heart disease and COVID-19: A meta-analysis. Med Clin (Barc). 2021;156(11):547–54.

Lim J, Jeon S, Shin HY, Kim MJ, Seong YM, Lee WJ, Choe KW, Kang YM, Lee B, Park SJ. Case of the Index Patient Who Caused Tertiary Transmission of COVID-19 Infection in Korea: the Application of Lopinavir/Ritonavir for the Treatment of COVID-19 Infected Pneumonia Monitored by Quantitative RT-PCR. J Korean Med Sci. 2020;35(6):e79.

Lippi G, Lavie CJ, Sanchis-Gomar F. Cardiac troponin I in patients with coronavirus disease 2019 (COVID-19): evidence from a meta-analysis. Prog Cardiovasc Dis. 2020;63(3):390–1.

Liu CX, Wang YL, Yan FY. Understanding novel coronavirus pneumonia, paying attention to research and development of epidemic prevention and control drugs. Chinese Journal of Antibiotics. 2020a;45(2):1-10.

Liu F, Liu F, Wang L. COVID-19 and cardiovascular diseases. J Mol Cell Biol. 2021a;13(3):161–7.

Liu J, Deswal A, Khalid U. COVID-19 myocarditis and long-term heart failure sequelae. Curr Opin Cardiol. 2021b;36(2):234–40.

Liu J, Huang L, Wei W, Bai Y, Chang E, Leng Y. Effects of antihypertensive agents on the clinical outcome of hospitalized COVID-19 patients concomitant with hypertension: a systematic review and meta-analysis. Heart Lung. 2024;63:78–85.

Liu K, Fang YY, Deng Y, Liu W, Wang MF, Ma JP, Xiao W, Wang YN, Zhong MH, Li CH, Li GC, Liu HG. Clinical characteristics of novel coronavirus cases in tertiary hospitals in Hubei Province. Chin Med J (Engl). 2020b;133(9):1025–31.

Loperena R, Van Beusecum JP, Itani HA, Engel N, Laroumanie F, Xiao L, Elijovich F, Laffer CL, Gnecco JS, Noonan J, Maffia P, Jasiewicz-Honkisz B, Czesnikiewicz-Guzik M, Mikolajczyk T, Sliwa T, Dikalov S, Weyand CM, Guzik TJ, Harrison DG. Hypertension and increased endothelial mechanical stretch promote monocyte differentiation and activation: roles of STAT3, interleukin 6 and hydrogen peroxide. Cardiovasc Res. 2018;114(11):1547–63.

Lu H. Drug treatment options for the 2019-new coronavirus (2019-nCoV). Biosci Trends. 2020a;14(1):69–71.

Lu R, Zhao X, Li J, Niu P, Yang B, Wu H, Wang W, Song H, Huang B, Zhu N, Bi Y, Ma X, Zhan F, Wang L, Hu T, Zhou H, Hu Z, Zhou W, Zhao L, Chen J, Meng Y, Wang J, Lin Y, Yuan J, Xie Z, Ma J, Liu WJ, Wang D, Xu W, Holmes EC, Gao GF, Wu G, Chen W, Shi W, Tan W. Genomic characterisation and epidemiology of 2019 novel coronavirus: implications for virus origins and receptor binding. Lancet. 2020b;395(10224):565–74.

Lucey DR. New treatments for Ebola virus disease. BMJ. 2019;366:l5371.

Lui G, Guaraldi G. Drug treatment of COVID-19 infection. Curr Opin Pulm Med. 2023;29(3):174–83.

Luo W, Liu X, Bao K, Huang C. Ischemic stroke associated with COVID-19: a systematic review and meta-analysis. J Neurol. 2022;269(4):1731–40.

Magesh S, John D, Li WT, Li Y, Mattingly-App A, Jain S, Chang EY, Ongkeko WM. Disparities in COVID-19 outcomes by race, ethnicity, and socioeconomic status: a systematic-review and meta-analysis. JAMA Netw Open. 2021;4(11):e2134147.

Majeed H, Gangu K, Shekhar R, Sagheer S, Garg I, Shuja H, Bobba A, Chourasia P, Avula SR, Sheikh AB. Impact of COVID-19 on patients hospitalized with ST-segment elevation myocardial infarction in the United States during the early pandemic: an analysis of outcomes, care delivery, and racial disparities in mortality. Infect Dis Rep. 2023;15(1):55–65.

Mann DL. Innate immunity and the failing heart: the cytokine hypothesis revisited. Circ Res. 2015;116(7):1254–68.

Manolis AS, Manolis AA, Manolis TA, Apostolopoulos EJ, Papatheou D, Melita H. COVID-19 infection and cardiac arrhythmias. Trends Cardiovasc Med. 2020;30(8):451–60.

Marbán E. A mechanistic roadmap for the clinical application of cardiac cell therapies. Nat Biomed Eng. 2018;2(6):353–61.

Marik PE, DePerrior SE, Ahmad Q, Dodani S. Gender-based disparities in COVID-19 patient outcomes. J Investig Med. 2021:jim-2020-001641. doi: 10.1136/jim-2020-001641.

Marvi TK, Stubblefield WB, Tillman BF, Tenforde MW, Patel MM, Lindsell CJ, Self WH, Grijalva CG, Rice TW. Serial thromboelastography and the development of venous thromboembolism in critically ill patients with COVID-19. Crit Care Explor. 2022;4(1):e0618.

Mauer N, Chiecca G, Carioli G, Gianfredi V, Iacoviello L, Bertagnolio S, Guerra R, Odone A, Signorelli C. The first 110,593 COVID-19 patients hospitalised in lombardy: a regionwide analysis of case characteristics, risk factors and clinical outcomes. Int J Public Health. 2022;67:1604427.

Mehta P, McAuley DF, Brown M, Sanchez E, Tattersall RS, Manson JJ; HLH Across Speciality Collaboration, UK. COVID-19: consider cytokine storm syndromes and immunosuppression. Lancet. 2020;395(10229):1033–4.

Melhorn J, Alamoudi A, Mentzer AJ, Fraser E, Fries A, Cassar MP, Kwok A, Knight JC, Raman B, Talbot NP, Petousi N. Persistence of inflammatory and vascular mediators 5 months after hospitalization with COVID-19 infection. Front Med (Lausanne). 2023;10:1056506.

Mietus-Snyder M, Suslovic W, Delaney M, Playford MP, Ballout RA, Barber JR, Otvos JD, DeBiasi RL, Mehta NN, Remaley AT. Changes in HDL cholesterol, particles, and function associate with pediatric COVID-19 severity. Front Cardiovasc Med. 2022;9:1033660.

Mohamed MS, Moulin TC, Schiöth HB. Sex differences in COVID-19: the role of androgens in disease severity and progression. Endocrine. 2021;71(1):3–8.

Montiel V, Lobysheva I, Gérard L, Vermeersch M, Perez-Morga D, Castelein T, Mesland JB, Hantson P, Collienne C, Gruson D, van Dievoet MA, Persu A, Beauloye C, Dechamps M, Belkhir L, Robert A, Derive M, Laterre PF, Danser AHJ, Wittebole X, Balligand JL. Oxidative stress-induced endothelial dysfunction and decreased vascular nitric oxide in COVID-19 patients. EBioMedicine. 2022;77:103893.

Morse JS, Lalonde T, Xu S, Liu WR. Learning from the past: possible urgent prevention and treatment options for severe acute respiratory infections caused by 2019-nCoV. Chembiochem. 2020;21(5):730–8.

Móvio MI, Almeida GWC, Martines IDGL, Barros de Lima G, Sasaki SD, Kihara AH, Poole E, Nevels M, Carlan da Silva MC. SARS-CoV-2 ORF8 as a modulator of cytokine induction: evidence and search for molecular mechanisms. Viruses. 2024;16(1):161.

Musher DM, Abers MS, Corrales-Medina VF. Acute infection and myocardial infarction. N Engl J Med. 2019;380(2):171–6.

Najafi N, Davoudi A, Izadyar H, Alishahi A, Mokhtariani A, Soleimanpourian B, Tabarrayi M, Moosazadeh M, Daftarian Z, Ahangarkani F. The effect of ACE inhibitors and ARBs on outcomes in hospitalized patients with COVID-19. Ir J Med Sci. 2023;192(3):1517–23.

Nakkazi E. Randomised controlled trial begins for Ebola therapeutics. Lancet. 2018;392(10162):2338.

Nanavaty D, Sinha R, Kaul D, Sanghvi A, Kumar V, Vachhani B, Singh S, Devarakonda P, Reddy S, Verghese D. Impact of COVID-19 on acute myocardial infarction: a national inpatient sample analysis. Curr Probl Cardiol. 2024;49(1 Pt A):102030.

Nuzzi V, Del Mestre E, Degrassi A, Bromage DI, Manca P, Piper S, Artico J, Gentile P, Scott PA, Chiatto M, Merlo M, Pareek N, Giacca M, Sinagra G, McDonagh TA, Cannata A. Cardiovascular damage in COVID-19: what we know two years later. Curr Cardiol Rep. 2022;24(9):1085–91.

Okwan-Duodu D, Lim EC, You S, Engman DM. TMPRSS2 activity may mediate sex differences in COVID-19 severity. Signal Transduct Target Ther. 2021;6(1):100.

O'Mahoney LL, Routen A, Gillies C, Ekezie W, Welford A, Zhang A, Karamchandani U, Simms-Williams N, Cassambai S, Ardavani A, Wilkinson TJ, Hawthorne G, Curtis F, Kingsnorth AP, Almaqhawi A, Ward T, Ayoubkhani D, Banerjee A, Calvert M, Shafran R, Stephenson T, Sterne J, Ward H, Evans RA, Zaccardi F, Wright S, Khunti K. The prevalence and long-term health effects of Long Covid among hospitalised and non-hospitalised populations: a systematic review and meta-analysis. EClinicalMedicine. 2022;55:101762.

Ozcan M, Zhu X, Zhang H, Javaheri A. Editorial: Lipids, lipoproteins and COVID-19. Front Cardiovasc Med. 2023;10:1293249.

Parodi JB, Indavere A, Bobadilla Jacob P, Toledo GC, Micali RG, Waisman G, Masson W, Epstein ED, Huerin MS. Impact of COVID-19 vaccination in post-COVID cardiac complications. Vaccine. 2023;41(8):1524–8.

Parvu S, Müller K, Dahdal D, Cosmin I, Christodorescu R, Duda-Seiman D, Man D, Sharma A, Dragoi R, Baneu P, Dragan S. COVID-19 and cardiovascular manifestations. Eur Rev Med Pharmacol Sci. 2022;26(12):4509–19.

Patone M, Mei XW, Handunnetthi L, Dixon S, Zaccardi F, Shankar-Hari M, Watkinson P, Khunti K, Harnden A, Coupland CAC, Channon KM, Mills NL, Sheikh A, Hippisley-Cox J. Risks of myocarditis, pericarditis, and cardiac arrhythmias associated with COVID-19 vaccination or SARS-CoV-2 infection. Nat Med. 2022;28(2):410–22.

Pellicori P, Doolub G, Wong CM, Lee KS, Mangion K, Ahmad M, Berry C, Squire I, Lambiase PD, Lyon A, McConnachie A, Taylor RS, Cleland JG. COVID-19 and its cardiovascular effects: a systematic review of prevalence studies. Cochrane Database Syst Rev. 2021;3(3):CD013879.

Phetsouphanh C, Darley DR, Wilson DB, Howe A, Munier CML, Patel SK, Juno JA, Burrell LM, Kent SJ, Dore GJ, Kelleher AD, Matthews GV. Immunological dysfunction persists for 8 months following initial mild-to-moderate SARS-CoV-2 infection. Nat Immunol. 2022;23(2):210–6.

Pimentel M, Magalhães APA, Novak CV, May BM, Rosa LGBD, Zimerman LI. Cardiac Arrhythmias in Patients with COVID-19. Arq Bras Cardiol. 2021;117(5):1010–5.

Prasad M, Leon M, Lerman LO, Lerman A. Viral endothelial dysfunction: a unifying mechanism for COVID-19. Mayo Clin Proc. 2021;96(12):3099–108.

Qian Y, Lei T, Patel PS, Lee CH, Monaghan-Nichols P, Xin HB, Qiu J, Fu M. Direct Activation of Endothelial Cells by SARS-CoV-2 Nucleocapsid Protein Is Blocked by Simvastatin. J Virol. 2021;95(23):e0139621.

Ren LL, Wang YM, Wu ZQ, Xiang ZC, Guo L, Xu T, Jiang YZ, Xiong Y, Li YJ, Li XW, Li H, Fan GH, Gu XY, Xiao Y, Gao H, Xu JY, Yang F, Wang XM, Wu C, Chen L, Liu YW, Liu B, Yang J, Wang XR, Dong J, Li L, Huang CL, Zhao JP, Hu Y, Cheng ZS, Liu LL, Qian ZH, Qin C, Jin Q, Cao B, Wang JW. Identification of a novel coronavirus causing severe pneumonia in human: A descriptive study. Chin Med J (Engl). 2020;133(9):1015–24.

Rodriguez F, Solomon N, de Lemos JA, Das SR, Morrow DA, Bradley SM, Elkind MSV, Williams JH, Holmes D, Matsouaka RA, Gupta D, Gluckman TJ, Abdalla M, Albert MA, Yancy CW, Wang TY. Racial and Ethnic Differences in Presentation and Outcomes for Patients Hospitalized With COVID-19: Findings From the American Heart Association's COVID-19 Cardiovascular Disease Registry. Circulation. 2021;143(24):2332–42.

Rossouw TM, Anderson R, Manga P, Feldman C. Emerging role of platelet-endothelium interactions in the pathogenesis of severe SARS-CoV-2 infection-associated myocardial injury. Front Immunol. 2022;13:776861.

Rothe C, Schunk M, Sothmann P, Bretzel G, Froeschl G, Wallrauch C, Zimmer T, Thiel V, Janke C, Guggemos W, Seilmaier M, Drosten C, Vollmar P, Zwirglmaier K, Zange S, Wölfel R, Hoelscher M. Transmission of 2019-nCoV infection from an asymptomatic contact in Germany. N Engl J Med. 2020;382(10):970–1.

Ruan Q, Yang K, Wang W, Jiang L, Song J. Clinical predictors of mortality due to COVID-19 based on an analysis of data of 150 patients from Wuhan, China. Intensive Care Med. 2020;46(5):846–8.

Sagris D, Papanikolaou A, Kvernland A, Korompoki E, Frontera JA, Troxel AB, Gavriatopoulou M, Milionis H, Lip GYH, Michel P, Yaghi S, Ntaios G. COVID-19 and ischemic stroke. Eur J Neurol. 2021;28(11):3826–36.

Saha S, Sharma K. Modification of lifestyle to recover from post-COVID symptoms: a short review. J Lifestyle Med. 2022;12(3):113–8.

Sala S, Peretto G, Gramegna M, Palmisano A, Villatore A, Vignale D, De Cobelli F, Tresoldi M, Cappelletti AM, Basso C, Godino C, Esposito A. Acute myocarditis presenting as a reverse Tako-Tsubo syndrome in a patient with SARS-CoV-2 respiratory infection. Eur Heart J. 2020;41(19):1861–2.

Samidurai A, Das A. Cardiovascular complications associated with COVID-19 and potential therapeutic strategies. Int J Mol Sci. 2020;21(18):6790.

Santoso A, Pranata R, Wibowo A, Al-Farabi MJ, Huang I, Antariksa B. Cardiac injury is associated with mortality and critically ill pneumonia in COVID-19: A meta-analysis. Am J Emerg Med. 2021;44:352–7.

Sawalha K, Abozenah M, Kadado AJ, Battisha A, Al-Akchar M, Salerno C, Hernandez-Montfort J, Islam AM. Systematic review of COVID-19 related myocarditis: insights on management and outcome. Cardiovasc Revasc Med. 2021;23:107–13.

Scannell CA, Oronce CIA, Tsugawa Y. Association between county-level racial and ethnic characteristics and COVID-19 cases and deaths in the USA. J Gen Intern Med. 2020;35(10):3126–8.

Schneider J, Jaenigen B, Wagner D, Rieg S, Hornuss D, Biever PM, Kern WV, Walz G. Therapy with lopinavir/ritonavir and hydroxychloroquine is associated with acute kidney injury in COVID-19 patients. PLoS One. 2021;16(5):e0249760.

Schoeman D, Fielding BC. Coronavirus envelope protein: current knowledge. Virol J. 2019;16(1):69.

Schultheiß C, Willscher E, Paschold L, Gottschick C, Klee B, Henkes SS, Bosurgi L, Dutzmann J, Sedding D, Frese T, Girndt M, Höll JI, Gekle M, Mikolajczyk R, Binder M. The IL-1β, IL-6, and TNF cytokine triad is associated with post-acute sequelae of COVID-19. Cell Rep Med. 2022;3(6):100663.

Schumacher SM, Naga Prasad SV. Tumor necrosis factor-alpha in heart failure: an updated review. Curr Cardiol Rep. 2018;20(11):117.

Shen KL, Yang YH. Diagnosis and treatment of 2019 novel coronavirus infection in children: a pressing issue. World J Pediatr. 2020;16(3):219–21.

Shi H, Zuo Y, Navaz S, Harbaugh A, Hoy CK, Gandhi AA, Sule G, Yalavarthi S, Gockman K, Madison JA, Wang J, Zuo M, Shi Y, Maile MD, Knight JS, Kanthi Y. Endothelial cell-activating antibodies in COVID-19. Arthritis Rheumatol. 2022;74(7):1132–8.

Shi S, Qin M, Shen B, Cai Y, Liu T, Yang F, Gong W, Liu X, Liang J, Zhao Q, Huang H, Yang B, Huang C. Association of cardiac injury with mortality in hospitalized patients with COVID-19 in Wuhan, China. JAMA Cardiol. 2020;5(7):802–10.

Shi Z, Fu W. Diagnosis and treatment recommendation for novel coronavirus pneumonia related isolated distal deep vein thrombosis. Shanghai Medical Journal. 2020b;43(4):207–10.

Shu H, Wen Z, Li N, Zhang Z, Ceesay BM, Peng Y, Zhou N, Wang DW. COVID-19 and cardiovascular diseases: from cellular mechanisms to clinical manifestations. Aging Dis. 2023;14(6):2071–88.

Shukla AK, Banerjee M. Angiotensin-converting-enzyme 2 and renin-angiotensin system inhibitors in COVID-19: an update. High Blood Press Cardiovasc Prev. 2021;28(2):129–39.

Siddiq S, Ahmed S, Akram I. Clinical outcomes following COVID-19 infection in ethnic minority groups in the UK: a systematic review and meta-analysis. Public Health. 2023;222:205–14.

Siedlinski M, Jozefczuk E, Xu X, Teumer A, Evangelou E, Schnabel RB, Welsh P, Maffia P, Erdmann J, Tomaszewski M, Caulfield MJ, Sattar N, Holmes MV, Guzik TJ. White blood cells and blood pressure: a Mendelian randomization study. Circulation. 2020;141(16):1307–17.

Silva MG, Corradi GR, Pérez Duhalde JI, Nuñez M, Cela EM, Gonzales Maglio DH, Brizzio A, Salazar MR, Espeche WG, Gironacci MM. Plasmatic renin-angiotensin system in normotensive and hypertensive patients hospitalized with COVID-19. Biomed Pharmacother. 2022;152:113201.

Singh B, Manita B, Suman F, Kumari N, Shiza ST, Samreen I, Shah S, Mokhtar SM, Patel U, Devi J, Reza RR, Mohamed KH, Ahmad S, Nasir H. A systematic review of COVID-19 vaccine-induced Takotsubo cardiomyopathy: a 2023 update. Cureus. 2023;15(12):e50319.

Siripanthong B, Nazarian S, Muser D, Deo R, Santangeli P, Khanji MY, Cooper LT Jr, Chahal CAA. Recognizing COVID-19-related myocarditis: The possible pathophysiology and proposed guideline for diagnosis and management. Heart Rhythm. 2020;17(9):1463–71.

Smer A, Squires RW, Bonikowske AR, Allison TG, Mainville RN, Williams MA. Cardiac complications of COVID-19 infection and the role of physical activity. J Cardiopulm Rehabil Prev. 2023;43(1):8–14

Sommerstein R, Kochen MM, Messerli FH, Gräni C. Coronavirus Disease 2019 (COVID- 19): Do Angiotensin-Converting Enzyme Inhibitors/Angiotensin Receptor Blockers Have a Biphasic Effect? J Am Heart Assoc. 2020;9(7):e016509.

Soriano JB, Murthy S, Marshall JC, Relan P, Diaz JV; WHO Clinical Case Definition Working Group on Post-COVID-19 Condition. A clinical case definition of post-COVID-19 condition by a delphi consensus. Lancet Infect Dis. 2022;22(4):e102–7.

Su S, Chen R, Zhang S, Shu H, Luo J. Immune system changes in those with hypertension when infected with SARS-CoV-2. Cell Immunol. 2022;378:104562.

Su YB, Kuo MJ, Lin TY, Chien CS, Yang YP, Chou SJ, Leu HB. Cardiovascular manifestation and treatment in COVID-19. J Chin Med Assoc. 2020;83(8):704–9.

Sun J, Deng X, Chen X, Huang J, Huang S, Li Y, Feng J, Liu J, He G. Incidence of adverse drug reactions in COVID‐19 patients in China: an active monitoring study by hospital pharmacovigilance system. Clin Pharmacol Ther. 2020;108(4):791–7.

Sze S, Pan D, Nevill CR, Gray LJ, Martin CA, Nazareth J, Minhas JS, Divall P, Khunti K, Abrams KR, Nellums LB, Pareek M. Ethnicity and clinical outcomes in COVID-19: A systematic review and meta-analysis. EClinicalMedicine. 2020;29:100630.

Tajbakhsh A, Gheibi Hayat SM, Taghizadeh H, Akbari A, Inabadi M, Savardashtaki A, Johnston TP, Sahebkar A. COVID-19 and cardiac injury: clinical manifestations, biomarkers, mechanisms, diagnosis, treatment, and follow up. Expert Rev Anti Infect Ther. 2021;19(3):345–57.

Takasu S, Ariizumi M, Matsumoto S, Nakagawa H, Iwadate K. Cerebral venous sinus thrombosis associated with COVID-19: an autopsy case report. Forensic Sci Med Pathol. 2022;18(1):80–5.

Tang N, Li D, Wang X, Sun Z. Abnormal coagulation parameters are associated with poor prognosis in patients with novel coronavirus pneumonia. J Thromb Haemost. 2020;18(4):844–7.

Tang Y, Hu L, Liu Y, Zhou B, Qin X, Ye J, Shen M, Wu Z, Zhang P. Possible mechanisms of cholesterol elevation aggravating COVID-19. Int J Med Sci. 2021;18(15):3533–43.

Tangos M, Jarkas M, Akin I, El-Battrawy I, Hamdani N. Cardiac damage and tropism of severe acute respiratory syndrome coronavirus 2. Curr Opin Microbiol. 2024;78:102437.

Thomas N, Gurvich C, Kulkarni J. Sex Differences and COVID-19. Adv Exp Med Biol. 2021;1327:79–91.

Tian W, Jiang W, Yao J, Nicholson CJ, Li RH, Sigurslid HH, Wooster L, Rotter JI, Guo X, Malhotra R. Predictors of mortality in hospitalized COVID-19 patients: A systematic review and meta-analysis. J Med Virol. 2020;92(10):1875–83.

Tobler DL, Pruzansky AJ, Naderi S, Ambrosy AP, Slade JJ. Long-term cardiovascular effects of COVID-19: emerging data relevant to the cardiovascular clinician. Curr Atheroscler Rep. 2022;24(7):563–70.

Tomasoni D, Italia L, Adamo M, Inciardi RM, Lombardi CM, Solomon SD, Metra M. COVID 19 and heart failure: from infection to inflammation and angiotensin II stimulation. Searching for evidence from a new disease. Eur J Heart Fail. 2020;22(6):957–66.

Torres C, García J, Meslé F, Barbieri M, Bonnet F, Camarda CG, Cambois E, Caporali A, Couppié É, Poniakina S, Robine JM. Identifying age- and sex-specific COVID-19 mortality trends over time in six countries. Int J Infect Dis. 2023;128:32–40.

Tsampasian V, Bäck M, Bernardi M, Cavarretta E, Dębski M, Gati S, Hansen D, Kränkel N, Koskinas K, Niebauer J, Spadafora L, Frias Vargas M, Biondi-Zoccai G, Vassiliou VS. Cardiovascular disease as part of Long COVID: A systematic review. Eur J Prev Cardiol. 2024:zwae070. doi: 10.1093/eurjpc/zwae070.

Tsampasian V, Elghazaly H, Chattopadhyay R, Debski M, Naing TKP, Garg P, Clark A, Ntatsaki E, Vassiliou VS. Risk factors associated with post−COVID-19 condition: a systematic review and meta-analysis. JAMA Intern Med. 2023;183(6):566–80.

Tsaplin S, Schastlivtsev I, Zhuravlev S, Barinov V, Lobastov K, Caprini JA. The original and modified Caprini score equally predicts venous thromboembolism in COVID-19 patients. J Vasc Surg Venous Lymphat Disord. 2021;9(6):1371–81.e4.

Tuvali O, Tshori S, Derazne E, Hannuna RR, Afek A, Haberman D, Sella G, George J. The incidence of myocarditis and pericarditis in post COVID-19 unvaccinated patients-a large population-based study. J Clin Med. 2022;11(8):2219.

Vaduganathan M, Vardeny O, Michel T, McMurray JJV, Pfeffer MA, Solomon SD. Renin-angiotensin-aldosterone system inhibitors in patients with COVID-19. N Engl J Med. 2020;382(17):1653–9.

Varga Z, Flammer AJ, Steiger P, Haberecker M, Andermatt R, Zinkernagel AS, Mehra MR, Schuepbach RA, Ruschitzka F, Moch H. Endothelial cell infection and endotheliitis in COVID-19. Lancet. 2020;395(10234):1417–8.

Varney JA, Dong VS, Tsao T, Sabir MS, Rivera AT, Ghula S, Moriles KE, Cherukuri ML, Fazal R, Azevedo CB, Mohamed RM, Jackson GR, Fleming SE, Rochez DE, Abbas KS, Shah JH, Minh LHN, Osman F, Rafla SM, Huy NT. COVID-19 and arrhythmia: An overview. J Cardiol. 2022;79(4):468–75.

Varshney AS, Omar WA, Goodrich EL, Bhatt AS, Wolley AE, Gong J, Senman BC, Silva D, Levangie MW, Berg DD, Yeh RW, de Lemos JA, Morrow DA, Kazi DS, Bohula EA. Epidemiology of cardiogenic shock in hospitalized adults with COVID-19: a report from the american heart association COVID-19 cardiovascular disease registry. Circ Heart Fail. 2021;14(12):e008477.

Vasichkina E, Alekseeva D, Kudryavtsev I, Glushkova A, Starshinova AY, Malkova A, Kudlay D, Starshinova A. COVID-19 Heart Lesions in Children: Clinical, Diagnostic and Immunological Changes. Int J Mol Sci. 2023;24(2):1147.

Viveiros A, Rasmuson J, Vu J, Mulvagh SL, Yip CYY, Norris CM, Oudit GY. Sex differences in COVID-19: candidate pathways, genetics of ACE2, and sex hormones. Am J Physiol Heart Circ Physiol. 2021;320(1):H296–H304.

Vosko I, Zirlik A, Bugger H. Impact of COVID-19 on cardiovascular disease. Viruses. 2023;15(2):508.

Walls AC, Park YJ, Tortorici MA, Wall A, McGuire AT, Veesler D. Structure, function, and antigenicity of the SARS-CoV-2 spike glycoprotein. Cell. 2020;181(2):281–92.e6.

Wan EYF, Yan VKC, Mok AHY, Wang B, Xu W, Cheng FWT, Lai FTT, Chui CSL, Li X, Wong CKH, Li PH, Cowling BJ, Hung IFN, Lau CS, Wong ICK, Chan EWY. Effectiveness of molnupiravir and nirmatrelvir-ritonavir in hospitalized patients with COVID-19 : a target trial emulation study. Ann Intern Med. 2023;176(4):505–14.

Wan Y, Shang J, Graham R, Baric RS, Li F. Receptor recognition by novel coronavirus from Wuhan: An analysis based on decade-long structural studies of SARS. J Virol. 2020;94(7):e00127-20.

Wang D, Hu B, Hu C, Zhu F, Liu X, Zhang J, Wang B, Xiang H, Cheng Z, Xiong Y, Zhao Y, Li Y, Wang X, Peng Z. Clinical characteristics of 138 hospitalized patients with 2019 novel coronavirus–infected pneumonia in Wuhan, China. JAMA. 2020a;323(11):1061–9.

Wang G, Zhang Q, Zhao X, Dong H, Wu C, Wu F, Yu B, Lv J, Zhang S, Wu G, Wu S, Wang X, Wu Y, Zhong Y. Low high-density lipoprotein level is correlated with the severity of COVID-19 patients: an observational study. Lipids Health Dis. 2020b;19(1):204.

Wang YD, Zhang SP, Wei QZ, Zhao MM, Mei H, Zhang ZL, Hu Y. COVID-19 complicated with DIC: 2 cases report and literatures review. Zhonghua Xue Ye Xue Za Zhi. 2020c;41(3):245–7.

Warchoł I, Dębska-Kozłowska A, Karcz-Socha I, Książczyk M, Szymańska K, Lubiński A. Terra incognita: clinically suspected myocarditis in a patient with severe acute respiratory syndrome coronavirus 2 infection. Pol Arch Intern Med. 2020;130(5):446–8.

Watanabe A, Iwagami M, Yasuhara J, Takagi H, Kuno T. Protective effect of COVID-19 vaccination against Long COVID syndrome: a systematic review and meta-analysis. Vaccine. 2023;41(11):1783–90.

Wehbe Z, Hammoud SH, Yassine HM, Fardoun M, El-Yazbi AF, Eid AH. Molecular and biological mechanisms underlying gender differences in COVID-19 severity and mortality. Front Immunol. 2021;12:659339.

Wichmann D, Sperhake JP, Lütgehetmann M, Steurer S, Edler C, Heinemann A, Heinrich F, Mushumba H, Kniep I, Schröder AS, Burdelski C, de Heer G, Nierhaus A, Frings D, Pfefferle S, Becker H, Bredereke-Wiedling H, de Weerth A, Paschen HR, Sheikhzadeh-Eggers S, Stang A, Schmiedel S, Bokemeyer C, Addo MM, Aepfelbacher M, Püschel K, Kluge S. Autopsy findings and venous thromboembolism in patients with COVID-19: A prospective cohort study. Ann Intern Med. 2020;173(4):268–77.

Wichmann D. Autopsy findings and venous thromboembolism in patients with COVID-19. Ann Intern Med. 2020;173(12):1030.

Widmann M, Gaidai R, Schubert I, Grummt M, Bensen L, Kerling A, Quermann A, Zacher J, Vollrath S, Bizjak DA, Beckendorf C, Egger F, Hasler E, Mellwig KP, Fütterer C, Wimbauer F, Vogel A, Schoenfeld J, Wüstenfeld JC, Kastner T, Barsch F, Friedmann-Bette B, Bloch W, Meyer T, Mayer F, Wolfarth B, Roecker K, Reinsberger C, Haller B, Niess AM; CoSmo-S Consortium. COVID-19 in female and male athletes: symptoms, clinical findings, outcome, and prolonged exercise intolerance-A Prospective, Observational, Multicenter Cohort Study (CoSmo-S). Sports Med. 2024 Jan 11. doi: 10.1007/s40279-023-01976-0.

Woodruff RC, Garg S, George MG, Patel K, Jackson SL, Loustalot F, Wortham JM, Taylor CA, Whitaker M, Reingold A, Alden NB, Meek J, Anderson EJ, Weigel A, Henderson J, Bye E, Davis SS, Barney G, Bennett NM, Shiltz E, Sutton M, Talbot HK, Price A, Sperling LS, Havers FP; COVID-19-Associated Hospitalization Surveillance Network. Acute cardiac events during COVID-19-associated hospitalizations. J Am Coll Cardiol. 2023;81(6):557–69.

Wu JT, Leung K, Bushman M, Kishore N, Niehus R, de Salazar PM, Cowling BJ, Lipsitch M, Leung GM. Estimating clinical severity of COVID-19 from the transmission dynamics in Wuhan, China. Nat Med. 2020;26(4):506–10.

Wu Q, Zhou L, Sun X, Yan Z, Hu C, Wu J, Xu L, Li X, Liu H, Yin P, Li K, Zhao J, Li Y, Wang X, Li Y, Zhang Q, Xu G, Chen H. Altered lipid metabolism in recovered SARS patients twelve years after infection. Sci Rep. 2017;7(1):9110.

Wu T, Zuo Z, Yang D, Luo X, Jiang L, Xia Z, Xiao X, Liu J, Ye M, Deng M. Venous thromboembolic events in patients with COVID-19: a systematic review and meta-analysis. Age Ageing. 2021;50(2):284–93.

Xiao H, Vaidya R, Liu F, Chang X, Xia X, Unger JM. Sex, racial, and ethnic representation in COVID-19 clinical trials: a systematic review and meta-analysis. JAMA Intern Med. 2023;183(1):50–60.

Xie Y, Bowe B, Al-Aly Z. Molnupiravir and risk of hospital admission or death in adults with COVID-19: emulation of a randomized target trial using electronic health records. BMJ. 2023a;380:e072705.

Xie Y, Bowe B, Al-Aly Z. Nirmatrelvir and risk of hospital admission or death in adults with covid-19: emulation of a randomized target trial using electronic health records. BMJ. 2023b;381:e073312.

Xie Y, Choi T, Al-Aly Z. Association of treatment with nirmatrelvir and the risk of post–COVID-19 condition. JAMA Intern Med. 2023c;183(6):554–564.

Xie Y, Choi T, Al-Aly Z. Molnupiravir and risk of post-acute sequelae of covid-19: cohort study. BMJ. 2023d;381:e074572.

Xu X, Chen P, Wang J, Feng J, Zhou H, Li X, Zhong W, Hao P. Evolution of the novel coronavirus from the ongoing Wuhan outbreak and modeling of its spike protein for risk of human transmission. Sci China Life Sci. 2020a;63(3):457–60.

Xu Z, Shi L, Wang Y, Zhang J, Huang L, Zhang C, Liu S, Zhao P, Liu H, Zhu L, Tai Y, Bai C, Gao T, Song J, Xia P, Dong J, Zhao J, Wang FS. Pathological findings of COVID-19 associated with acute respiratory distress syndrome. Lancet Respir Med. 2020b;8(4):420–422.

Yancy CW. COVID-19 and African Americans. JAMA. 2020;323(19):1891–2.

Yao XH, Li TY, He ZC, Ping YF, Liu HW, Yu SC, Mou HM, Wang LH, Zhang HR, Fu WJ, Luo T, Liu F, Guo QN, Chen C, Xiao HL, Guo HT, Lin S, Xiang DF, Shi Y, Pan GQ, Li QR, Huang X, Cui Y, Liu XZ, Tang W, Pan PF, Huang XQ, Ding YQ, Bian XW. A pathological report of three COVID-19 cases by minimally invasive autopsies. Zhonghua Bing Li Xue Za Zhi. 2020;49(5):411–7.

Yeo YH, Wang M, He X, Lv F, Zhang Y, Zu J, Li M, Jiao Y, Ebinger JE, Patel JK, Cheng S, Ji F. Excess risk for acute myocardial infarction mortality during the COVID-19 pandemic. J Med Virol. 2023;95(1):e28187.

Yokoo P, Fonseca EKUN, Sasdelli Neto R, Ishikawa WY, Silva MMA, Yanata E, Chate RC, Nunes Filho ACB, Bettega M, Fernandes JRC, Tarasoutchi F, Szarf G. COVID-19 myocarditis: a case report. Einstein (Sao Paulo). 2020;18:eRC5876.

Youn JC, Yu HT, Lim BJ, Koh MJ, Lee J, Chang DY, Choi YS, Lee SH, Kang SM, Jang Y, Yoo OJ, Shin EC, Park S. Immunosenescent CD8+ T cells and C-X-C chemokine receptor type 3 chemokines are increased in human hypertension. Hypertension. 2013;62(1):126–33.

Yu L, Liu Y, Feng Y. Cardiac arrhythmia in COVID-19 patients. Ann Noninvasive Electrocardiol. 2024;29(2):e13105.

Yu WL, Toh HS, Liao CT, Chang WT. A double-edged sword-cardiovascular concerns of potential anti-COVID-19 drugs. Cardiovasc Drugs Ther. 2021 Apr;35(2):205–14.

Zeng JH, Liu YX, Yuan J, Wang FX, Wu WB, Li JX, Wang LF, Gao H, Wang Y, Dong CF, Li YJ, Xie XJ, Feng C, Liu L. First case of COVID- 19 complicated with fulminant myocarditis: a case report and insights. Infection. 2020;48(5):773–7.

Zhang H, Kang Z, Gong H, Xu D, Wang J, Li Z, Li Z, Cui X, Xiao J, Zhan J, Meng T, Zhou W, Liu J, Xu H. Digestive system is a potential route of COVID-19: an analysis of single-cell coexpression pattern of key proteins in viral entry process. Gut. 2020;69(6): 1010–8.

Zhang H, Shao L, Lin Z, Long QX, Yuan H, Cai L, Jiang G, Guo X, Yang R, Zhang Z, Zhang B, Liu F, Li Z, Ma Q, Zhang YW, Huang AL, Wang Z, Zhao Y, Xu H. APOE interacts with ACE2 inhibiting SARS-CoV-2 cellular entry and inflammation in COVID-19 patients. Signal Transduct Target Ther. 2022;7(1):261.

Zhang S, Zhang J, Wang C, Chen X, Zhao X, Jing H, Liu H, Li Z, Wang L, Shi J. COVID-19 and ischemic stroke: Mechanisms of hypercoagulability (Review). Int J Mol Med. 2021;47(3):21.

Zhang Z, Wu S, Wang Z, Wang Y, Chen H, Wu C, Xiong L. Long-term oral ACEI/ARB therapy is associated with disease severity in elderly COVID-19 omicron BA.2 patients with hypertension. BMC Infect Dis. 2023;23(1):882.

Zhao S, Lin Q, Ran J, Musa SS, Yang G, Wang W, Lou Y, Gao D, Yang L, He D, Wang MH. Preliminary estimation of the basic reproduction number of novel coronavirus (2019-nCoV) in China, from 2019 to 2020: A data-driven analysis in the early phase of the outbreak. Int J Infect Dis. 2020;92:214–7.

Zhao Y, Han X, Li C, Liu Y, Cheng J, Adhikari BK, Wang Y. COVID-19 and the cardiovascular system: a study of pathophysiology and interpopulation variability. Front Microbiol. 2023;14:1213111.

Zheng YY, Ma YT, Zhang JY, Xie X. COVID-19 and the cardiovascular system. Nat Rev Cardiol. 2020;17(5):259–60.

Zhong LL, Wong YP, Leung CY, Peng B, Lin ZX, Wong Taam VC, Luo Y, Chen HY, Chao CD, Wong CF, Tam FS, Chan K, Lee KY, Ho LF, Wong AY, Choy CF, Ng BF, Wong RH, Feng YB, Liong C, Bian ZX; COVID-19 CM Research Working Group. Effects of Chinese medicine for COVID-19 rehabilitation: a multicenter observational study. Chin Med. 2022;17(1):99.

Zhou F, Yu T, Du R, Fan G, Liu Y, Liu Z, Xiang J, Wang Y, Song B, Gu X, Guan L, Wei Y, Li H, Wu X, Xu J, Tu S, Zhang Y, Chen H, Cao B. Clinical course and risk factors for mortality of adult inpatients with COVID-19 in Wuhan, China: a retrospective cohort study. Lancet. 2020a;395(10229):1054–62.

Zhou X, Li Y, Yang Q. Antiplatelet therapy after percutaneous coronary intervention in patients with COVID-19: implications from clinical features to pathologic findings. Circulation. 2020b;141(22):1736–8.

Zuin M, Mazzitelli M, Rigatelli G, Bilato C, Cattelan AM. Risk of ischemic stroke in patients recovered from COVID-19 infection: A systematic review and meta-analysis. Eur Stroke J. 2023;8(4):915–22.

Zuin M, Rigatelli G, Roncon L, Pasquetto G, Bilato C. Risk of incident heart failure after COVID-19 recovery: a systematic review and meta-analysis. Heart Fail Rev. 2023;28(4):859-864.
